# Plasma neurofilament light chain and regional brain atrophy mediate the association of neuropsychiatric symptoms with cognition in Alzheimer’s disease: evidence from two population-based studies

**DOI:** 10.1017/S003329172610484X

**Published:** 2026-06-15

**Authors:** Ya-Yu Wang, Huai-Yuan Zhu, Wei Miao, Zhi-Xin Wang, Jia-Jia Qin, Xiao-Han Song, Yue-Hua Wang, Zhong-Wu Sun, Xia Zhou, Xian-Feng Yu, Xiao-Qun Zhu, for the Alzheimer’s Disease Neuroimaging Initiative

**Affiliations:** 1 Department of Neurology, The First Affiliated Hospital of Anhui Medical University, Hefei, China; 2 Department of Clinical Pharmacy, The First Affiliated Hospital of Henan Medical University, Weihui, China

**Keywords:** Alzheimer’s Disease, brain structures, cognition, neurofilament light, neuropsychiatric symptoms

## Abstract

**Background:**

Neuropsychiatric symptoms (NPSs) are prevalent in Alzheimer’s disease (AD), yet their neurobiological etiology remains elusive. We investigated relationships between NPS subsyndromes, plasma neurofilament light chain (NFL), AD-vulnerable brain atrophy, and cognition.

**Methods:**

We included 146 participants from a Chinese cohort. NPSs were assessed using the Neuropsychiatric Inventory Questionnaire and clustered into four subsyndromes (hyperactivity, psychosis, affective, apathy), each graded by severity (none, mild, severe). Sequential mediation analyses examined whether NPSs influence cognition through NFL and atrophy. Additionally, 1534 ADNI participants were enrolled to (1) replicate mediation effects; (2) examine longitudinal relationships of NPSs with incident cognitive decline; and (3) evaluate cognitive discrimination of NPSs and NFL and onset hazards of NPS subsyndromes.

**Results:**

In the discovery cohort, global NPSs burden and three subsyndromes (hyperactivity, affective, apathy) were associated with elevated plasma NFL, poorer global cognition and memory, and reduced brain volumes (all *P* < 0.05). Sequential mediation revealed that plasma NFL and atrophy mediated the cross-sectional NPSs–cognition relationship, replicated in ADNI. Adding NPSs and NFL improved cognitive discrimination (AUC: 0.6754 to 0.8210, *P* < 0.001). Additionally, apathy and psychosis showed lower onset hazards than affective and hyperactivity (both *P* < 0.001).

**Conclusions:**

Baseline NPSs were cross-sectionally associated with elevated NFL, brain atrophy, and poorer cognition. Sequential mediation models supported a pathway linking NPSs to cognition via NFL and atrophy, though longitudinal evidence did not fully confirm temporal directionality. These hypothesis-generating findings require prospective validation.

## Background

Alzheimer’s disease (AD) is characterized by amyloid-*β* (*Aβ*) and hyperphosphorylated tau accumulation and primarily manifests as cognitive impairment. However, an estimated 80% of AD patients also experience one or more neuropsychiatric symptoms (NPSs) (Lyketsos et al., 2002), which contribute substantially to reduced quality of life, earlier institutionalization (Shin et al., 2005), and greater caregiver burden (Isik et al., 2019). Notably, certain NPSs, such as anxiety and apathy, may even precede cognitive impairments (Becker et al., 2018; Craig et al., 2005), indicating their potential role as early diagnostic markers and promising targets for preventive interventions in AD. Despite their clinical significance, the neurobiological basis of NPSs and AD-related pathologies remains incompletely understood.

NPSs appear intrinsic to AD neuropathology rather than merely secondary to cognitive impairment (Masters et al., 2015). Mild behavioral impairment, characterized by persistent NPSs, has been shown to predict accelerated progression to mild cognitive impairment (MCI) and dementia (Creese & Ismail, 2022). A recent autopsy study further demonstrated that NPSs increased with greater neuropathologic burden (Cholerton et al., 2025). However, findings across studies have been inconsistent (Showraki et al., 2019). Some studies report associations between specific NPSs (e.g. apathy and anxiety) and cerebrospinal fluid (CSF) Aβ/tau abnormalities or brain atrophy (Banning et al., 2020; Krell-Roesch et al., 2023), while others do not (Arenare et al., 2023). This heterogeneity underscores the complex relationship between NPSs and AD. Additionally, NPSs tend to emerge at a distinct stage (Chen et al., 2021). Better understanding of their subsyndrome-specific onset patterns and clinical discriminative utility in AD may help identify stage-specific interventions and personalized management.

NPSs may also promote inflammatory dysregulation, with mechanisms involving glial activation (Schaffer Aguzzoli et al., 2023) and overproduction of inflammatory cytokines (Clark et al., 2022). Neurofilament light chain (NFL), a biomarker of neuroaxonal damage across neurological diseases (Gaetani et al., 2019; Khalil et al., 2018; Lewczuk et al., 2018; Olsson et al., 2019; Zetterberg et al., 2016), is detectable in both CSF and peripheral blood following neuroinflammatory processes (Gaetani et al., 2019; Khalil et al., 2018). Although plasma NFL correlates with cognitive decline and brain atrophy (Lewczuk et al., 2018; Mattsson et al., 2016; Olsson et al., 2019; Zetterberg et al., 2016), its association with NPSs remains inconclusive (Rabl et al., 2024). Importantly, region-specific neurodegeneration, reflected by NFL-related axonal injury, may promote NPSs through structural alterations in AD-vulnerable regions like the hippocampus (Bloniecki et al., 2020). Nevertheless, an integrated framework simultaneously evaluating NPSs, plasma NFL, neurodegeneration in AD-vulnerable brain regions, and cognition has yet to be established. Elucidating these multidimensional relationships is essential for understanding the mechanisms by which NPSs influence cognition in AD.

Given that NPSs clusters (subsyndromes) may better reflect underlying neuropathology than isolated symptoms (Lyketsos et al., 2011), the present study aimed to: (1) examine the associations of baseline NPSs (global burden, and hyperactivity, psychosis, affective, and apathy subsyndromes) with plasma NFL, brain atrophy, and cognitive performance; (2) investigate the sequential mediation effect of plasma NFL and atrophy linking NPSs to cognitive performance; these analyses were performed based on an independent Chinese cohort and the ADNI (Alzheimer’s Disease Neuroimaging Initiative) database; (3) evaluate the discriminative value of NPSs and plasma NFL for concurrent cognitive status; and (4) compare onset hazards of NPS subsyndromes using longitudinal ADNI data. Our findings are expected to facilitate a better understanding of NPSs in AD and inform future studies on early intervention and personalized management.

## Methods

### Study population

**Figure 1.**
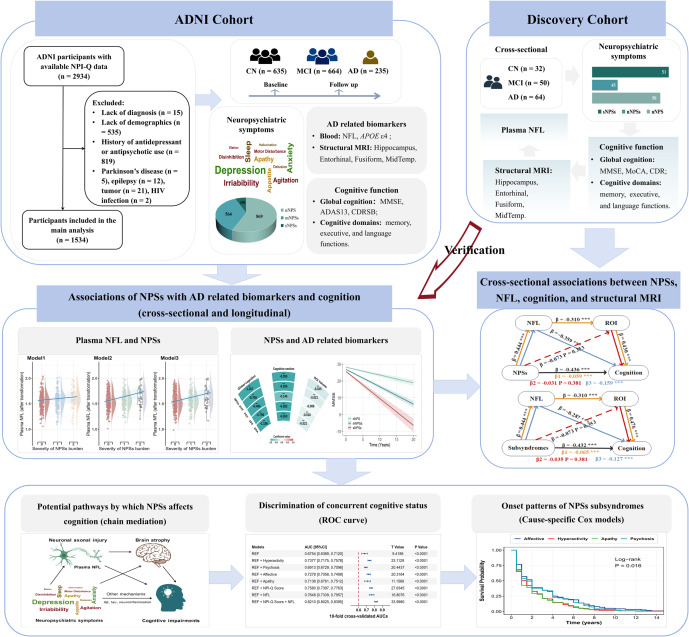
A schematic overview of the study design and analysis pipeline. *Note*: ADNI, ‘Alzheimer’s Disease Neuroimaging Initiative’; NPI-Q, ‘Neuropsychiatric Inventory Questionnaire’; CN, ‘cognitively normal’; MCI, ‘mild cognitive impairment’; AD, ‘Alzheimer’s Disease’; NFL, ‘neurofilament light chain’; *APOE* ε4, ‘apolipoprotein ε4’; MRI, ‘magnetic resonance imaging’; MMSE, ‘Mini-Mental State Examination’; ADAS13, ‘Alzheimer’s Disease Assessment Scale 13’; CDRSB, ‘Clinical Dementia Rating Sum of Boxes’; MEM, ‘Memory function’; LAN, ‘Language’; EF, ‘Executive function’; MoCA, ‘Montreal Cognitive Assessment’; NPSs, ‘neuropsychiatric symptoms’; ROC, ‘receiver operating characteristic’. Figure 1. long description.

As the discovery cohort, an independent cohort was recruited from the First Affiliated Hospital of Anhui Medical University (ethical approval number: Quick-PJ 2023-08-39). Of the 310 individuals initially screened, 146 participants of Chinese Han origin (right-handed, aged 50–85 years) were included in the final analysis, comprising 32 cognitively normal controls (CN), 50 MCI, and 64 AD. All participants completed neuropsychological assessments, demographic interviews, laboratory tests, and sMRI scans (Figure 1). Detailed diagnostic criteria, as well as inclusion and exclusion criteria, are provided in the Supplementary Material eMethods. The study was approved by the institutional ethics committee, and all participants provided written informed consent after receiving a thorough explanation of the procedures.

**Table 1.** Baseline demographic characteristics of participants in the ADNI cohort Table 1. long description.

	NPSs (global burden)			Hyperactivity			Psychosis			Affective			Apathy		
	None	Mild	Severe	None	Mild	Severe	None	Mild	Severe	None	Mild	Severe	None	Mild	Severe
Age (y)	73.40 (7.19)	73.89 (7.31)	72.96 (7.83)	73.42 (7.24)	74.05 (7.55)	73.67 (7.16)	73.61 (7.23)	73.05 (8.12)	73.41 (6.71)	73.66 (7.23)	73.05 (7.28)	73.47 (7.89)	73.38 (7.29)	75.15 (7.19)^a^	73.60 (7.03)
Male (n, %)	454 (52.24)	324 (60.64) ^a^	74 (73.27)^a^	575 (51.20)	166 (69.75)^a^	129 (74.57)^a^	745 (57.35)	74 (54.41)	51 (51.52)	659 (55.29)	154 (61.35)	59 (64.84)	599 (46.04)	36 (27.48)^a^	29 (28.43)^a^
Education (y)	16.22 (2.69)	15.94 (2.83)	15.81 (2.58)	16.20 (2.67)	15.79 (2.84)	15.80 (2.96)	16.16 (2.71)	15.52 (3.16)	15.91 (2.42)	16.19 (2.69)	15.63 (2.91)^a^	15.99 (2.75)	16.15 (2.75)	15.83 (2.72)	15.66 (2.62)
*APOE*ε4 Carriers (n, %)	333 (38.32)	269 (47.70) ^a^	64 (63.37) ^a^^,^^b^	457 (40.69)	118 (49.58)^a^	91 (52.60)^a^	544 (41.88)	71 (52.21)^a^	51 (51.52)	416 (34.90)	140 (55.78)^a^	50 (54.95)^a^	542 (41.66)	68 (51.91)	56 (54.90)^a^
Lifestyles															
Smoking (n, %)	241 (27.73)	176 (31.21)	28 (27.72)	314 (27.96)	76 (31.93)	55 (31.79)	370 (28.48)	49 (36.03)	26 (26.26)	332 (27.85)	87 (34.66)	26 (28.57)	376 (28.90)	42 (32.06)	27 (26.47)
Drinking (n, %)	27 (3.11)	8 (1.42)	7 (6.93)^b^	30 (2.67)	5 (2.10)	7 (4.05)	36 (2.77)	5 (3.68)	1 (1.01)	31 (2.60)	9 (3.59)	2 (2.20)	33 (2.54)	5 (3.82)	4 (3.92)
Medical comorbidities															
Hypertension (n, %)	395 (45.45)	258 (45.74)	56 (55.45)	503 (44.79)	113 (47.48)	93 (53.76)	601 (46.27)	57 (41.91)	51 (51.52)	534 (44.80)	127 (50.60)	48 (52.75)	591 (45.43)	62 (47.33)	56 (54.90)
Stroke (n, %)	10 (1.15)	4 (7.09)	5 (4.95)^a^,^b^	314 (27.96)	76 (31.93)	55 (31.79)	17 (1.31)	0 (0)	2 (2.02)	14 (1.17)	3 (1.20)	2 (2.20)	14 (1.08)	2 (1.53)	3 (2.94)
Cognitive function															
MMSE	28.21 (2.08)	26.80 (2.70) ^a^	25.60 (3.38)^a^^,^^b^	27.88 (2.39)	26.78 (2.62)^a^	26.15 (2.92)^a^	27.65 (2.48)	26.94 (2.73)^a^	26.60 (3.05)^a^	27.80 (2.44)	26.57 (2.74)^a^	26.35 (2.75)^a^	27.83 (2.33)	26.12 (2.77)^a^	25.32 (3.35)^a^
ADAS13	12.87 (7.78)	18.35 (9.80)^a^	22.86 (11.38)^a^^,^^b^	14.03 (8.87)	18.68 (8.99)^a^	21.05 (10.23)^a^	15.02 (9.07)	17.79 (10.21)^a^	19.36 (11.23)^a^	14.30 (8.92)	19.35 (9.66)^a^	21.36 (9.96)^a^	14.32 (8.57)	21.47 (9.76)^a^	23.50 (12.12)^a^
CDRSB	0.73 (1.22)	1.90 (1.78)^a^	3.15 (2.03)^a^^,^^b^	0.95 (1.45)	1.97 (1.64)^a^	2.78 (2.01)^a^^,^^b^	1.20 (1.60)	1.86 (1.99)^a^	2.06 (1.91)^a^	1.04 (1.54)	2.10 (1.70)^a^	2.76 (1.91)^a^^,^^b^	1.04 (1.43)	2.51 (1.99)^a^	3.33 (2.13)^a^^,^^b^
MEM	0.48 (0.67)	0.01 (0.71)^a^	−0.32 (0.70)^a^^,^^b^	0.39 (0.72)	−0.05 (0.65)^a^	−0.20 (0.67)^a^	0.30 (0.72)	0.05 (0.76)^a^	0.01 (0.77)^a^	0.36 (0.72)	−0.07 (0.67)^a^	−0.20 (0.69)^a^	0.35 (0.70)	−0.24 (0.66)^a^	−0.34 (0.76)^a^
LAN	0.57 (0.63)	0.25 (0.65)^a^	0.04 (0.65)^a^^,^^b^	0.50 (0.66)	0.25 (0.62)^a^	0.07 (0.62)^a^^,^^b^	0.44 (0.66)	0.32 (0.71)	0.17 (0.68)^a^	0.48 (0.67)	0.22 (0.62)^a^	0.09 (0.58)^a^	0.48 (0.64)	0.08 (0.61)^a^	−0.08 (0.69)^a^
EF	0.59 (0.66)	0.21 (0.73)^a^	−0.02 (0.75)^a^^,^^b^	0.50 (0.71)	0.22 (0.71)^a^	0.07 (0.69)^a^	0.44 (0.71)	0.28 (0.76)^a^	0.14 (0.77)^a^	0.49 (0.71)	0.16 (0.71)^a^	0.08 (0.69)^a^	0.49 (0.70)	0.02 (0.66)^a^	−0.08 (0.79)^a^
Biomarkers															
NFL (pg/ml)	37.71 (19.08)	41.76 (19.52)^a^	37.53 (8.30)	40.93 (28.05)	45.21 (28.88)	43.00 (19.94)	42.45 (28.79)	37.73 (17.11)	53.93 (22.55)	40.58 (27.18)	46.83 (28.74)^a^	47.19 (27.07)	40.05 (24.47)	55.82 (42.33)^a^	39.89 (16.32)
AD-signature ROI	−0.30 (1.51)	0.36 (1.78)^a^	0.68 (1.77)^a^	−0.15 (1.60)	0.38 (1.77)^a^	0.52 (1.79)^b^	−0.05 (1.64)	0.30 (1.85)	0.27 (1.66)	−0.12 (1.61)	0.30 (1.83)^a^	0.79 (1.64)^a^^,^^b^	−0.14 (1.59)	0.81 (1.84)^a^	0.72 (1.87)^a^
CN/MCI/AD (n)	512/302/55	117/316/131^a^	6/46/49^a^^,^^b^	577/432/114	38/145/55^a^	20/87/66^a^^,^^b^	576/547/176	34/69/33^a^	25/48/26^a^	589/469/134	34/150/67^a^	12/45/34^a^	614/549/138	13/75/43^a^	8/40/54^a^^,^^b^
Follow-up number	798	519	91	1031	219	158	1190	128	90	1102	226	80	1197	121	90
Follow-up time (y)	5.21 (3.83)	4.07 (3.23)^a^	3.26 (2.58)^a^^,^^b^	4.95 (3.73)	4.04 (3.24)^a^	3.69 (3.00)^a^	4.78 (3.66)	4.41 (3.60)	3.50 (2.70)^a^	4.95 (3.71)	3.91 (3.14)^a^	2.93 (2.49)^a^^,^^b^	4.89 (3.70)	3.93 (3.03)^a^	2.64 (2.01)^a^^,^^b^

*Note*: Categorical variables are reported as numbers (percentages); continuous variables are reported as means (SDs). Between-group comparisons were performed using ANOVA or Kruskal–Wallis tests for continuous variables, and chi-square or Fisher’s exact tests for categorical variables, with post hoc analyses and multiple testing corrections as appropriate.

a *P* < 0.05 versus none-NPS.

b *P* < 0.05 versus mild NPSs.

*NPSs, ‘neuropsychiatric symptoms’; APOE ε4, ‘apolipoprotein ε4’; MMSE, ‘Mini-Mental State Examination’; ADAS13, ‘Alzheimer’s Disease Assessment Scale 13’; CDRSB, ‘Clinical Dementia Rating Sum of Boxes’; MEM, ‘Memory function’; LAN, ‘Language’; EF, ‘Executive function’; NFL, ‘neurofilament light chain’; CN, ‘cognitively normal’; MCI, ‘mild cognitive impairment’; AD, ‘Alzheimer’s Disease’; ROI, ‘region of interest’; SD, ‘standard deviation’.*

Data for validation were sourced from the ADNI database (Weiner et al., 2012). ADNI is established to evaluate clinical, imaging, genetic, and biochemical biomarkers for AD. Written informed consent was obtained from all participants or their authorized representatives following approval by the institutional review boards of participating centers. Detailed information is publicly available at https://adni.loni.usc.edu/. After applying exclusion criteria to 2,934 individuals with baseline Neuropsychiatric Inventory (NPI) data, 1,534 participants were included (mean follow-up 4.28 ± 3.68 years; see Figure 1 for details). Participants receiving antidepressant or antipsychotic medications at baseline were excluded. Sex was self-reported by all participants. Based on predefined criteria (Aisen et al., 2010), participants were classified as CN (*n* = 635; Mini-Mental State Examination [MMSE] > 24, Clinical Dementia Rating Sum of Boxes [CDRSB] = 0), MCI (*n* = 664; MMSE > 24, CDR = 0.5), or AD dementia (*n* = 235). Longitudinal data included NPI assessments, plasma biomarkers, a battery of cognitive tests, and structural magnetic resonance imaging (sMRI) scans.

### Assessment of neuropsychiatric symptoms

NPSs were assessed using the informant-based Neuropsychiatric Inventory Questionnaire (NPI-Q), which evaluates 12 common neuropsychiatric and behavioral symptoms occurring over the preceding 4 weeks (Cummings, 1997). Following established categorization, these symptoms cluster into four subsyndromes: hyperactivity (euphoria, agitation, irritability, disinhibition, and aberrant motor behaviors), psychosis (hallucination, delusion, and nighttime behavior disturbances), affective (anxiety and depression), and apathy (apathy and appetite changes) (Lyketsos et al., 2011).

Although a total NPI-Q score > 0 is frequently used as a dichotomous indicator of NPSs’ presence, this cutoff offers high sensitivity but limited specificity for identifying clinically meaningful symptoms in neurodegenerative disease. To enhance the accuracy of identification, we adopted a more stringent classification based on prior work (Cummings et al., 1994; Nunes et al., 2019): no (score = 0), mild (score 1–9), and severe symptoms (score ≥ 10). Similarly, the severity of each subsyndrome was categorized as none (score = 0), mild (score 1–2), or severe (score ≥ 3). The severe threshold (≥ 3) was set as the mean score of symptomatic individuals for that subsyndrome (Arenare et al., 2023), ensuring a clinically meaningful cutoff. This grading strategy facilitates a more nuanced examination of the clinical and biological characteristics of NPSs across different severity levels. In the present study, we focused primarily on the global NPSs burden and the four subsyndromes.

### Measurements of plasma biomarkers

Our team has published a methodology on plasma-derived biomarker assessment (Cao et al., 2024). Briefly, fasting blood samples were collected from participants, processed (centrifugation and aliquoting), and stored at −80 °C until further analysis. Apolipoprotein E (*APOE*) ε4 genotyping was performed using a BigDye™ Direct Cycle Sequencing Kit (Applied Biosystems™, USA) according to the manufacturer’s protocol. Plasma NFL concentrations were quantified using a single-molecule array (Simoa) HD-X analyzer (Quanterix, USA) at Nanjing Simcere Medical Diagnostics Co., Ltd. To ensure consistency, all assays were performed in a single experimental batch by board-certified technicians who were blinded to clinical data. Both intra- and inter-batch coefficients of variation were below 8.45%.

In ADNI, plasma NFL levels were measured on a Simoa platform (Quanterix Corp) at the University of Gothenburg. The assay range (6.7–1620.0 ng/L) encompassed all but one sample, as previously described (Mattsson et al., 2019). Intra- and inter-assay coefficients of variation were 6.2% and 9.0% at 11.0 ng/L, and 4.9% and 7.2% at 173.0 ng/L. All measurements were performed between January and April 2018 by a board-certified laboratory technician using a single reagent batch. Detailed assay protocols and raw data are accessible through the ADNI database.

### Cognitive assessment

In the discovery cohort, global cognition was evaluated using the Chinese version of MMSE, Montreal Cognitive Assessment (MoCA), and CDR. Domain-specific composite scores for episodic memory (MEM), language (LAN), and executive function (EF) were calculated by averaging z-scores from multiple tests within each cognitive domain. Detailed information is provided in the Supplementary material (eMethods).

In ADNI, cognitive functions were evaluated through a battery of scales. Global cognition was assessed with the MMSE, the Alzheimer’s Disease Assessment Scale-Cognitive Subscale 13 (ADAS13), and the Clinical Dementia Rating Sum of Boxes (CDRSB). Domain-specific cognition was assessed using neuropsychological test batteries, with composite scores reflecting MEM, LAN, and EF (Crane et al., 2012; Gibbons et al., 2012).

### Structural magnetic resonance imaging measures

High-resolution 3D T1-weighted structural images were acquired on a 3.0-Tesla MR scanner (Discovery MR750w, General Electric, Milwaukee, WI, USA) equipped with a 24-channel head coil in our discovery cohort. To minimize motion artifacts, participants’ heads were stabilized with foam padding, and they were instructed to remain still throughout the scan. A brain volume (BRAVO) sequence was used with the following parameters: repetition time (TR) = 8.5 ms, echo time (TE) = 3.2 ms, inversion time (TI) = 450 ms, flip angle = 12°, field of view (FOV) = 256 × 256 mm^2^, matrix size = 256 × 256, slice thickness = 1 mm (no gap), and 188 sagittal slices. To ensure data quality, all images were visually checked, and any with apparent artifacts were excluded from further analysis. For the ADNI cohort, T1-weighted images were acquired on Siemens Verio 1.5 or 3-Tesla scanners using a standardized protocol. Cortical reconstruction and volumetric segmentation were conducted using FreeSurfer software on T1-weighted images (Jack et al., 2008). Several AD-vulnerable brain regions (including hippocampus, middle temporal lobe, entorhinal cortex, and fusiform gyrus) (Jack et al., 2017; Jack et al., 2018) were selected as regions of interest (ROI) for analysis in both cohorts.

### Statistical analyses

Outliers exceeding ±3 standard deviations (SD) from the mean were removed. Descriptive statistics are presented as mean (SD) or frequency (percentages). Group comparisons were performed using ANOVA or Kruskal–Wallis tests for continuous variables, and chi-square or Fisher’s exact tests for categorical variables, as appropriate. Non-normally distributed biomarker data were log_10_-transformed prior to analysis. All primary analyses were adjusted for age, sex, education, and *APOE* ε4 status, with intracranial volume (ICV) additionally included for brain structure analyses. The overall study design is illustrated in Figure 1.

Firstly, we examined cross-sectional associations of baseline NPSs (global burden and four subsyndromes, as independent variables) with plasma NFL, brain structures, and cognition (dependent variables) using multiple linear regression. Three models were constructed: Model 1 (primary model) included the core covariates described above; Model 2 further adjusted for smoking, alcohol use, hypertension, and stroke (and diabetes in the discovery cohort); Model 3 additionally adjusted for cognitive status (discovery cohort) or ATN framework (ADNI, based on CSF Aβ_42_ and p-tau) (Hansson et al., 2018).

Next, we performed multiple mediation models utilizing sequential mediation analyses to examine whether plasma NFL and neurodegeneration mediated the relationship between NPSs and cognition in both cohorts. To support the temporal ordering of the proposed pathway, we first conducted two preliminary analyses using ADNI data (which have a larger sample size and longitudinal follow-up): Locally Estimated Scatterplot Smoothing (LOESS) regression to explore the order of biomarker abnormality emergence, and Cross-Lagged Panel Models (CLPM) to formally test temporal directionality. Neurodegeneration was operationalized as a composite index of AD-signature ROIs, derived by principal component analysis (PCA) of four predefined AD-vulnerable regions (Moscoso et al., 2021). This composite approach reduced the multiple-testing burden and captured NPSs-related cognitive decline from a network-degeneration perspective, thereby facilitating subsequent mediation analyses. We evaluated the significance of mediation effects using bootstrapping with 5,000 iterations, defining a significant indirect effect as a 95% confidence interval that does not contain zero.

Thirdly, longitudinal relationships between NPSs and plasma NFL, brain structures, and cognition were assessed using linear mixed-effects models in ADNI. These models included fixed effects for time (continuous), baseline NPS severity, and their interaction, with random intercepts and slopes for time and an unstructured covariance matrix for the random effects.

To further validate the clinical relevance of the key variables in our mediation model, we conducted receiver operating characteristic (ROC) analysis to assess their ability of NPSs and NFL in discriminating cognitively unimpaired (CU) and cognitively impaired (CI) individuals beyond a reference model comprising age, sex, education, and *APOE* ε4 status. To obtain optimism-corrected estimates, we performed 10-fold cross-validation and report the cross-validated area under the curve (AUC) as the primary result. The apparent AUC from the full dataset is provided for comparison. Sensitivity and specificity were determined using the Youden index.

Finally, we explored the heterogeneity in the onset patterns of NPS subsyndromes after excluding participants with any baseline NPSs. Onset was defined as the first follow-up visit with a subsyndrome score > 0. Kaplan–Meier curves with log-rank tests compared cumulative incidence across subsyndromes. Cause-specific Cox models (reference: affective; other subsyndromes censored) compared onset hazards. Sensitivity analyses included Fine–Gray models for competing risks and time-dependent Cox models for time-varying effects.

The ‘lm,’ ‘nlme,’ ‘pROC,’ ‘ggplot2,’ ‘FactoMineR,’ ‘lavaan,’ ‘survival,’ ‘cmprsk’ and ‘survminer’ packages in R (version 4.4.3) were used for statistical analyses and figure preparation. Statistical significance was defined as a two-tailed *P* < 0.05 after false discovery rate (FDR) correction for multiple comparisons, unless otherwise specified.

## Results

### Characteristics of participants

Baseline clinical and demographic characteristics of the discovery (*n* = 146) and ADNI cohort (*n* = 1,534) are presented in Supplementary Table S1 and Table 1, respectively. In the discovery cohort, participants had a mean age of 66.66 (8.41) years, 60.27% were male, and the mean education was 8.64 (4.37) years. Individuals with NPSs exhibited higher plasma NFL and poorer cognition. In the ADNI cohort, the sample comprised 870 males (56.71%) and 666 *APOE* ε4 carriers (43.42%), with mean age and education of 73.55 (7.27) and 16.09 (2.74) years, respectively. NPI-Q total scores increased across diagnostic groups (CN: 0.66 [2.00], MCI: 2.43 [4.67], AD: 5.61 [6.90]). Similarly, participants with NPSs exhibited elevated plasma NFL levels and poorer cognitive performance. In both cohorts, AD patients had a higher prevalence of all four NPS subsyndromes than CN and MCI individuals.

### Associations between NPSs and plasma NFL

**Figure 2.**
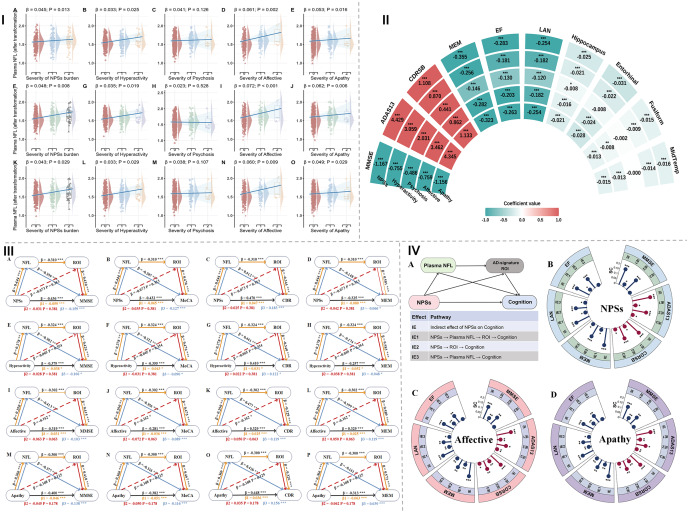
Associations of NPSs with plasma NFL, brain structures, and cognition. (I) Cross-sectional associations between NPSs and plasma NFL in ADNI. Associations were analyzed using multiple linear regression, with Model 1 adjusted for age, sex, education, and *APOE* ε4 status; Model 2 additionally adjusted for smoking, alcohol use, and history of hypertension and stroke; and Model 3 further adjusted for biological status using the ATN framework (based on CSF Aβ_42_ and p-tau). Multiple testing significance was corrected using FDR. (II) Cross-sectional associations between baseline NPSs and cognition and brain structures in ADNI. Associations were analyzed using multiple linear regression, with adjustments for age, sex, education, and *APOE* ε4 status. Intracranial volume was additionally included as a covariate when brain structure measures were the dependent variables. Multiple testing significance was corrected using FDR. (III) Sequential mediation of plasma NFL and brain structures linking NPSs to cognition in the discovery cohort. Three mediation pathways were examined: (1) NPSs → plasma NFL → AD-signature brain atrophy → cognition; (2) NPSs → AD-signature brain atrophy → cognition; and (3) NPSs → plasma NFL → cognition. Mediation pathways were assessed using a bootstrap test with 5,000 resamples. All models were adjusted for age, sex, education, and *APOE* ε4 status, with intracranial volume further adjusted for brain structure analyses. Non-significant indirect pathways (*P* ≥ 0.05) are depicted with dotted lines, whereas significant pathways (*P* < 0.05) are shown with solid lines. (IV) Sequential mediation of plasma NFL and brain structures in the associations between baseline NPSs and cognition in ADNI. Mediation pathways were assessed using a bootstrap test with 5,000 resamples. All models were adjusted for age, sex, education, and *APOE* ε4 status, with intracranial volume further adjusted for brain structure analyses. Lollipop charts illustrate the standardized coefficient (SC) for each pathway. The vertical line represents the absolute SC value, and the circle color indicates the direction of association (red: positive; blue: negative). Asterisks denote raw *P* values (presented without multiple comparison adjustment to preserve exploratory insights); FDR-corrected significance is reported in the main text. *P < 0.05, **P < 0.01, and ***P < 0.001. *Note*: NPSs, ‘neuropsychiatric symptoms’; NFL, ‘Neurofilament Light chain’; MMSE, ‘Mini-Mental State Examination’; ADAS13, ‘Alzheimer’s Disease Assessment Scale 13’; CDRSB, ‘Clinical Dementia Rating Sum of Boxes’; MEM, ‘Memory function’; LAN, ‘Language’; EF, ‘Executive function’; ADNI, ‘Alzheimer’s Disease Neuroimaging Initiative’; *APOE* ε4, ‘apolipoprotein ε4’; MoCA, ‘Montreal Cognitive Assessment’; FDR, ‘false discovery rate’. Figure 2. long description.

In the discovery cohort, individuals with NPSs had higher plasma NFL levels (*β* = 0.12, *P* < 0.001; Supplementary Table S2). Specifically, the hyperactivity, affective, and apathy subsyndromes were each positively associated with elevated plasma NFL (*β* = 0.09, *P* = 0.002; *β* = 0.09, *P* = 0.007; *β* = 0.133, *P* < 0.001; respectively), whereas the association between psychosis and NFL disappeared after FDR correction (*β* = 0.08, *P* = 0.135; Supplementary Table S2). These findings were replicated in the ADNI cohort, where global NPSs burden was positively associated with elevated plasma NFL (*β* = 0.05, *P* = 0.013, Figure 2I.A). Consistent with the discovery cohort, significant associations were observed for hyperactivity (*β* = 0.03, *P* = 0.025), affective (*β* = 0.06, *P* = 0.002), and apathy (*β* = 0.05, *P* = 0.016), but not for psychosis (*β* = 0.04, *P* = 0.126; Figure 2I.B–E, Supplementary Table S3). Almost all these associations persisted in sensitivity analyses adjusting for additional confounders (Figure 2I.F–O; Supplementary Tables S4–S7).

### Associations between NPSs and cognitive performance

Cross-sectional analyses in the discovery cohort revealed that NPSs were associated with worse global cognition (MMSE, *β* = −3.68, *P* < 0.001; MoCA, *β* = −3.96, *P* < 0.001; CDR, *β* = 0.30, *P* < 0.001) and MEM (*β* = −0.38, *P* < 0.001; Supplementary Figure S1). Similar associations emerged for hyperactivity, affective, and apathy subsyndromes, whereas no significant associations emerged for the psychosis subsyndrome (Supplementary Table S2). Notably, the significant association between apathy and LAN disappeared after adjusting for additional confounders (Supplementary Tables S4 and S6).

These associations were validated and extended in the ADNI cohort. Global NPSs burden and all four subsyndromes were significantly associated with poorer cognition across global (MMSE, ADAS13, and CDRSB) and domain-specific measures (MEM, LAN, and EF) (all *P* < 0.001; Figure 2II). Virtually all associations remained robust in fully adjusted models (Supplementary Tables S5 and S7).

### Associations between NPSs and brain structures

In the discovery cohort, individuals with NPSs exhibited lower volumes in several AD-vulnerable regions, including the hippocampus (*β* = −0.35, *P* = 0.021), fusiform (*β* = −0.53, *P* = 0.020), and middle temporal (*β* = −1.04, *P* = 0.022). Subsyndrome-specific analyses revealed that hyperactivity was associated with hippocampal volume reduction (*β* = −0.64, *P* = 0.032); the affective subsyndrome was associated with entorhinal cortex atrophy (*β* = −0.43, *P* = 0.017); and apathy was associated with volume loss in both hippocampus (*β* = −0.93, *P* < 0.001) and middle temporal (*β* = −1.95, *P* = 0.012) (Supplementary Figure S1 and Supplementary Table S2). These associations remained significant after additional adjustments (Model 2; Supplementary Table S4) but were attenuated in Model 3 (Supplementary Table S6). In ADNI, greater global NPS burden, as well as the hyperactivity, affective, and apathy subsyndromes, were consistently associated with lower volumes in the four AD-vulnerable brain regions (Figure 2II). In contrast, the psychosis subsyndrome showed no significant associations with any brain structure in either cohort.

### Mediation among NPSs, plasma NFL, neurodegeneration, and cognitive performance

LOESS regression revealed that NPSs’ abnormality emerged at the earliest stage along the AD continuum, followed by NFL and atrophy (Supplementary Figure S2). CLPM further showed that baseline NPSs significantly predicted subsequent NFL (*P* = 0.027), and NFL over time predicted subsequent atrophy (*P* = 0.037), supporting the first two steps of the proposed sequence (Table S8). PCA identified the first principal component (PC1) as capturing the shared variance across the four AD-vulnerable brain regions and was subsequently employed as a composite neurodegeneration index in mediation analyses.

In the discovery cohort, sequential mediation models were constructed to test three specific pathways: (1) *NPSs → plasma NFL→ AD-signature ROI → cognition*, (2) *NPSs→ AD-signature ROI → cognition*, and (3) *NPSs → plasma NFL → cognition.* Cross-sectional analyses revealed that the associations of baseline NPSs burden with global cognition (MMSE, MoCA, CDR) and MEM were mediated by both the sequential NFL-ROI pathway (Pathway 1) and plasma NFL alone (Pathway 3) (total indirect effect: all *P* < 0.05; Figure 2III.A–D). In contrast, no significant mediation was observed for the AD-signature ROI alone (Pathway 2). These sequential mediation effects were consistently observed for hyperactivity, affective, and apathy (Figure 2III.E–P) subsyndromes (total indirect effect: all *P* < 0.05).

In addition, these sequential mediation effects were largely replicated in ADNI (Figure 2IV). Cross-sectionally, the associations of baseline global NPSs burden with global (MMSE, ADAS13, CDRSB) and domain-specific cognitive performance (MEM, LAN, EF) were mediated by both the sequential NFL-ROI pathway (Pathway 1) and the AD-signature ROI alone (Pathway 2), with all effects surviving FDR correction (all *P* < 0.05). Plasma NFL alone (Pathway 3) showed nominally significant mediation effects for ADAS13, CDRSB, MEM, and LAN (total indirect effect: all uncorrected *P* < 0.05) (Figure 2IV.A, B; Table S9); however, after FDR correction, only the mediation effect on MEM remained significant (*P* < 0.05). In contrast to the discovery cohort, these sequential mediation effects were significant only for the affective and apathy subsyndromes (total indirect effect: *P* < 0.05; Figure 2IV.C, D) even after FDR correction.

Besides, sex-stratified analyses further revealed that the sequential mediation effects linking baseline NPSs to cognition were specific to females and remained significant after FDR correction (total indirect effect: global NPSs burden, *P* < 0.001; affective, *P* < 0.05; apathy, *P* < 0.05) (Supplementary Tables S10 and S11).

### Longitudinal associations between NPSs and plasma NFL, cognitive performance, and brain structures

**Figure 3.**
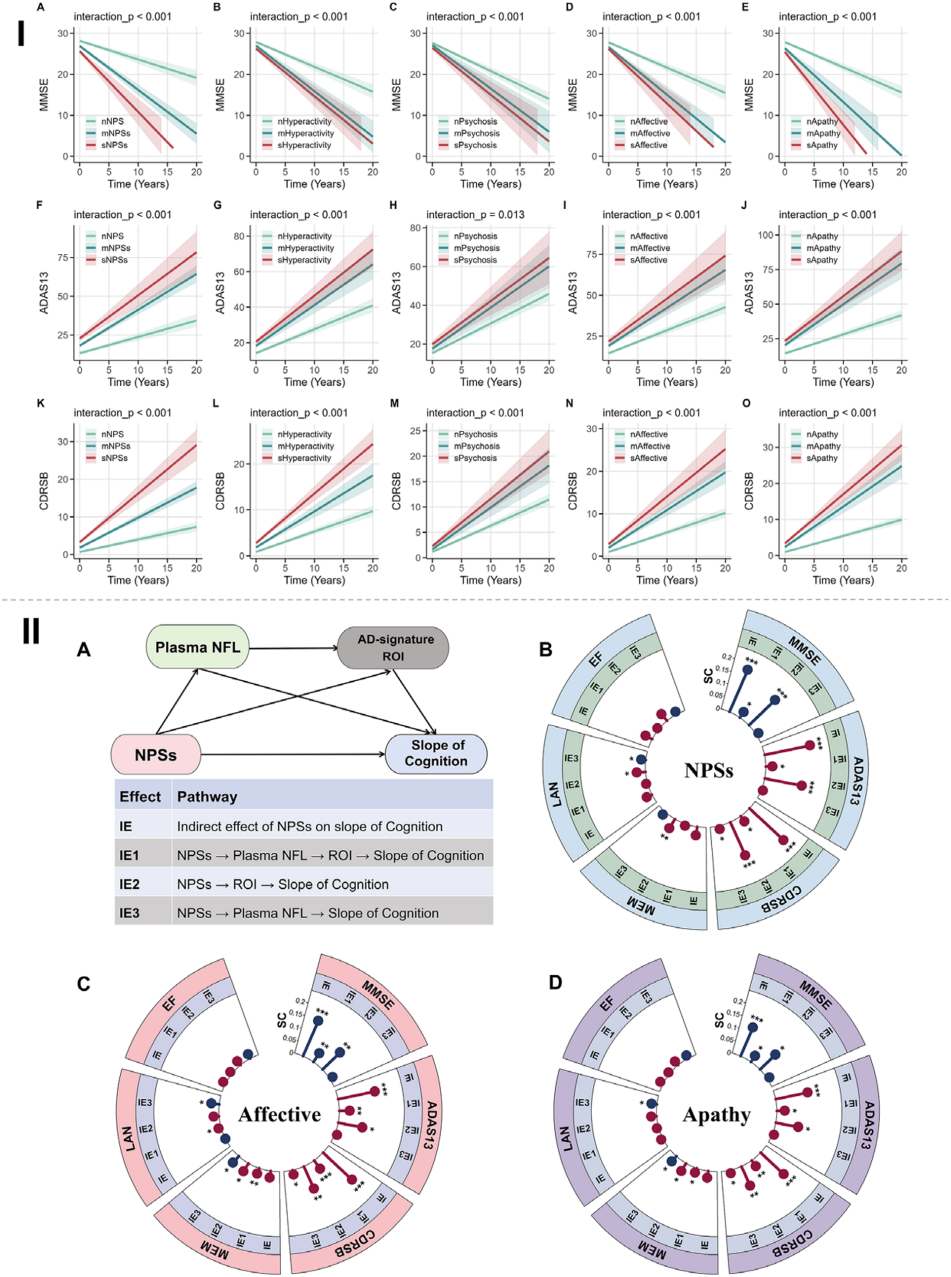
Relationships of baseline NPSs with plasma NFL, brain structures, and cognitive decline in ADNI. (I) Longitudinal associations between baseline NPSs and cognitive trajectories. Associations were analyzed using mixed-effects models, with adjustments for age, sex, education, and *APOE* ε4 status. Multiple testing correction was performed using FDR. (II) Sequential mediation of plasma NFL and brain structures linking NPSs to the slope of cognition. Mediation pathways were assessed using a bootstrap test with 5,000 resamples. All models were adjusted by age, sex, education, and *APOE* ε4 status, with intracranial volume further adjusted for brain structure analyses. Lollipop charts illustrate the standardized coefficient (SC) for each pathway. The vertical line represents the absolute SC value, and the circle color indicates the direction of association (red: positive; blue: negative). Asterisks denote raw *P* values (presented without multiple comparison adjustment to preserve exploratory insights); FDR-corrected significance is reported in the main text. *P < 0.05, **P < 0.01, and ***P < 0.001. NPSs, ‘neuropsychiatric symptoms’; MMSE, ‘Mini-Mental State Examination’; ADAS13, ‘Alzheimer’s Disease Assessment Scale 13’; CDRSB, ‘Clinical Dementia Rating Sum of Boxes’; MEM, ‘Memory function’; LAN, ‘Language’; EF, ‘Executive function’; ADNI, ‘Alzheimer’s Disease Neuroimaging Initiative’; *APOE* ε4, ‘apolipoprotein ε4’; FDR, ‘false discovery rate’. Figure 3. long description.

Longitudinal analyses of ADNI data showed that baseline NPSs were not significantly associated with the rate of change in plasma NFL or brain atrophy (data not shown). In contrast, greater baseline NPSs severity predicted faster global cognitive decline in a dose–response manner (severe > mild > none; all *P* for interaction <0.05), with similar trajectories observed across all four subsyndromes (Figure 3I).

Longitudinal mediation analyses revealed several nominally significant mediation effects before FDR correction (Figure 3II.A). For global NPSs burden and the hyperactivity, affective, and apathy subsyndromes, both the sequential NFL-ROI pathway (Pathway 1) and the AD-signature ROI alone (Pathway 2) showed nominally significant mediation effects on the slope of global cognition (MMSE, ADAS13, CDRSB) (total indirect effect: all *P* < 0.05). Plasma NFL alone (Pathway 3) nominally mediated the associations of global NPS burden, as well as the affective and apathy subsyndromes, with the slope of CDRSB specifically (Figure 3II.B–D).

After FDR correction, the mediation effects differed across pathways and subsyndromes. For Pathway 1, significant mediation effects on cognitive decline persisted for the affective subsyndrome across all global cognitive measures (MMSE, ADAS13, and CDRSB) and MEM, whereas for the apathy subsyndrome, the mediation effect was retained only for the slope of CDRSB (all corrected *P* < 0.05). For Pathway 2, atrophy significantly mediated the link between NPSs (global burden, hyperactivity, affective, and apathy) and global cognitive decline (all corrected *P* < 0.05). In contrast, none of the mediation effects via plasma NFL alone (Pathway 3) survived FDR correction (all corrected *P* > 0.05; Supplementary Table S12). Taken together, these longitudinal mediation results provide partial and subsyndrome-specific support for the proposed pathways.

Sex-stratified analyses revealed distinct patterns. In females, significant sequential mediation effects (Pathway 1 and Pathway 2) were observed exclusively for the affective subsyndrome (total indirect effect: corrected *P* < 0.05). In males, by contrast, these mediation effects (Pathway 2) were significant only for the hyperactivity subsyndrome (total indirect effect: corrected *P* < 0.05; Supplementary Tables S13 and S14).

### Clinical validation of NPSs and NFL as discriminative markers

**Figure 4.**
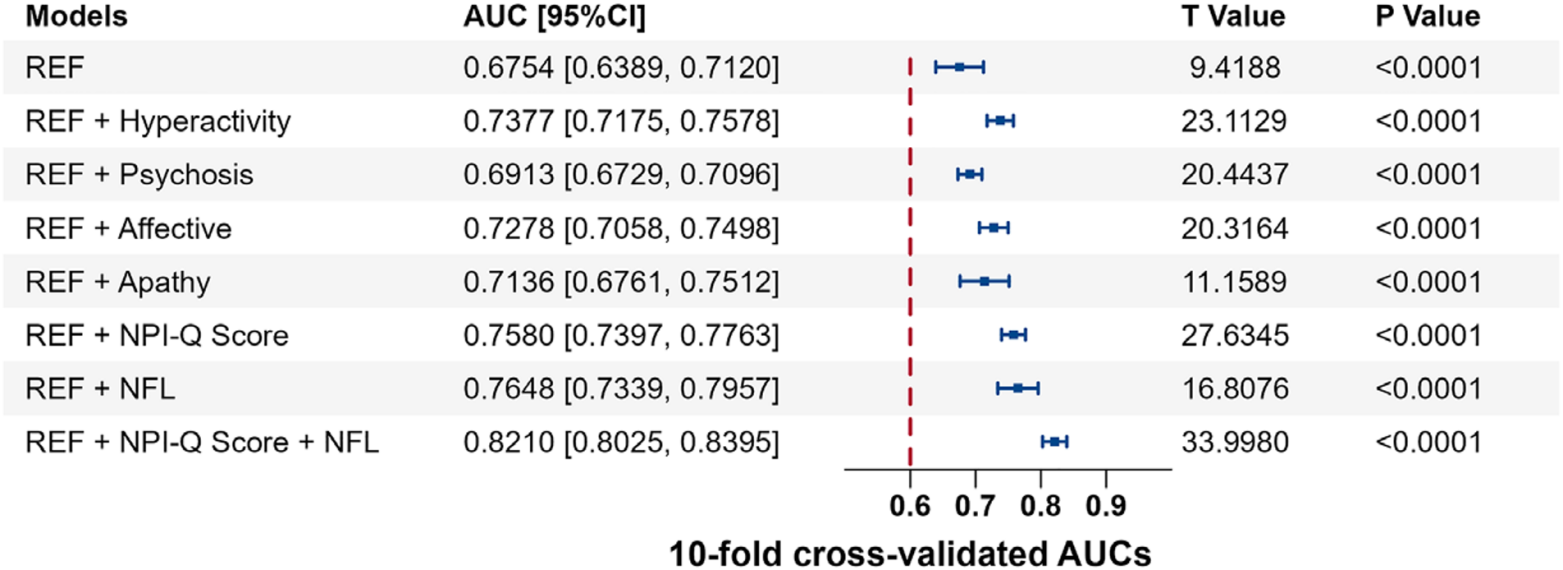
Cross-validated AUC estimates for discrimination of concurrent cognitive status. Forest plots displaying the 10-fold cross-validated AUC for each prediction model. Squares represent the point estimates of AUC, and error bars indicate the corresponding 95% CIs. One-sample t-tests were performed against AUC = 0.5 (no discriminative ability). Corresponding t-statistics and *P* values are reported. The REF model included age, sex, education, and *APOE* ε4 status. Adding NPSs (NPI-Q total score) and plasma NFL progressively improved discriminative performance. REF, reference model; NPI-Q, Neuropsychiatric Inventory Questionnaire; NFL, neurofilament light chain; *APOE* ε4, apolipoprotein ε4; ROC, receiver operating characteristic; AUC, area under the curve; CI, confidence interval. Figure 4. long description.

To validate the clinical relevance of the key variables in our mediation model, we tested whether NPSs and NFL could discriminate concurrent cognitive status. ROC analysis showed that adding NPSs and NFL progressively improved the ability to discriminate between CU and CI individuals, with AUC increasing from 0.6754 (reference model alone) to 0.8210 (reference + NPSs + NFL; Figure 4). For the optimal model, 10-fold cross-validation yielded a mean AUC of 0.8210 (95% CI: 0.8025–0.8395), with minimal optimism (0.0017), indicating good generalizability. Sensitivity and specificity improved from 58.55% and 75.16% to 76.42% and 76.09%, respectively (Supplementary Table S15).

### Heterogeneity of NPS subsyndromes in onset risk

**Figure 5.**
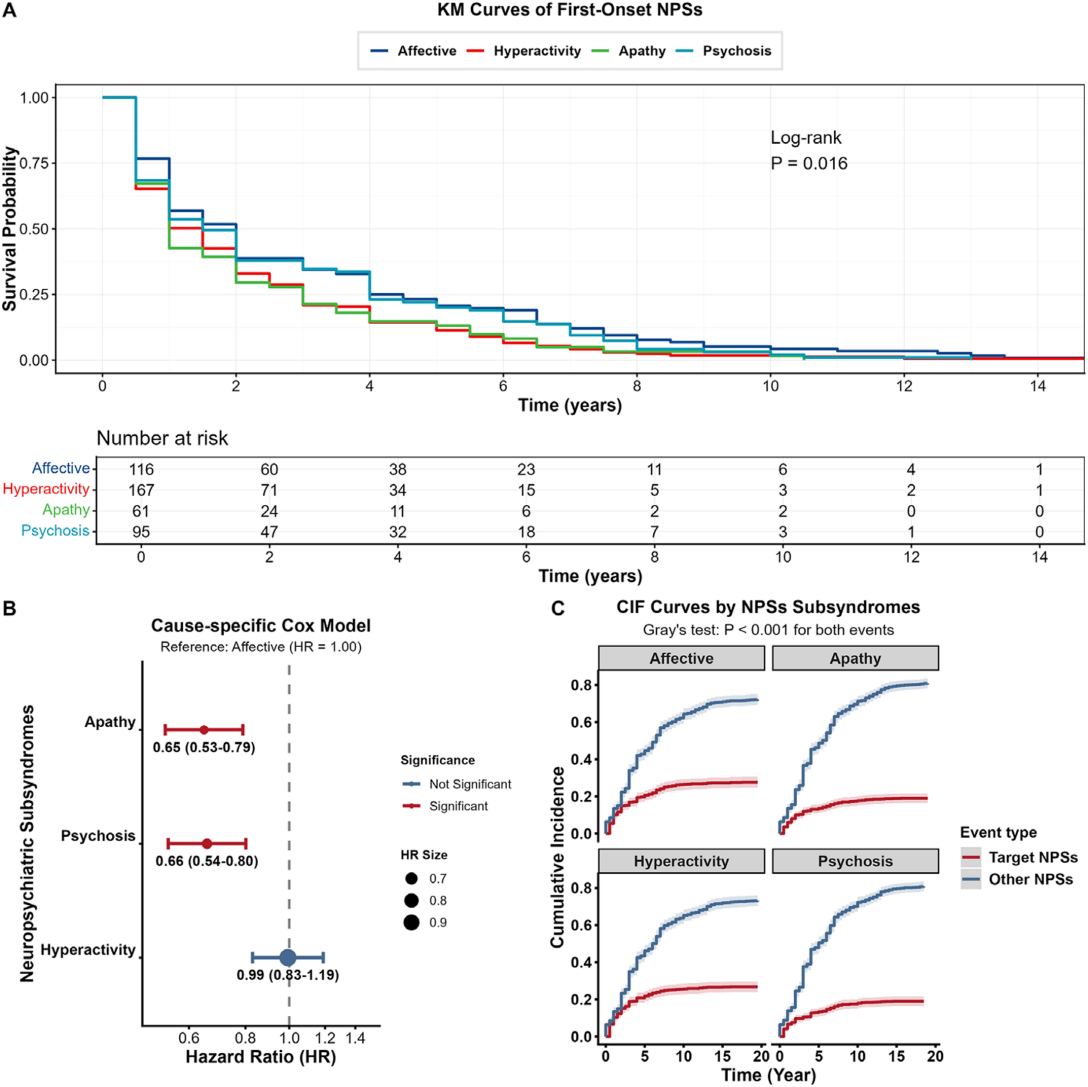
Temporal patterns of NPS subsyndrome onset. (A) Kaplan–Meier curves of first-onset NPS subsyndromes. The curves show the cumulative survival probability for each subsyndrome. Each participant contributed only their first-onset subsyndrome. The log-rank test indicated a significant overall difference among the four subsyndromes (*P* = 0.016). (B) Forest plot of the cause-specific Cox model for NPS subsyndrome onset. HRs and 95% CIs were estimated with adjustment for age, sex, education, and *APOE* ε4; the affective subsyndrome served as the reference (HR = 1.00). Squares represent HRs; error bars indicate 95% CIs. (C) Cumulative incidence function (CIF) curves for NPS subsyndromes with competing risks. Gray’s test for competing risks confirmed significant overall differences among subsyndromes (*P* < 0.001). The curves account for the fact that developing one subsyndrome may preclude or alter the risk of developing another. *Note*: NPSs, ‘neuropsychiatric symptoms’; CI, ‘confidence interval’; *APOE*ε4, ‘apolipoprotein ε4’. Figure 5. long description.

Given the heterogeneous effects of different NPSs’ subsyndromes observed above, we further compared their onset hazards. After excluding participants with any baseline NPSs (*n* = 870; mean follow-up: 5.4 years), Kaplan–Meier analysis showed significant differences among subsyndromes (log-rank *P* = 0.016; Figure 5A). Cause-specific Cox models revealed that apathy (HR = 0.65, 95% CI: 0.53–0.79, *P* < 0.001) and psychosis (HR = 0.66, 95% CI: 0.54–0.80, *P* < 0.001) had significantly lower onset hazards than affective, whereas hyperactivity showed no difference (HR = 0.99, *P* = 0.94) (Figure 5B). Fine–Gray competing risk models confirmed the robustness of these findings (Gray’s test: *P* < 0.001; Figure 5C). Time-dependent Cox models showed no significant time-varying effects for any covariate (all *P* > 0.05), supporting the proportional hazards assumption (Supplementary Table S16).

## Discussion

The present study demonstrated that baseline NPSs (both global NPSs burden and specific subsyndromes) were cross-sectionally associated with elevated plasma NFL, AD-vulnerable regional atrophy, and poorer cognitive performance across the AD continuum in an independent Chinese cohort, and these associations were verified in ADNI. Sequential mediation analyses were consistent with a pathway linking NPSs to cognition via NFL and atrophy, particularly evident for apathy and affective subsyndromes. Longitudinally, baseline NPSs predicted accelerated cognitive decline. A model combining NPSs and plasma NFL improved the discrimination of concurrent cognitive impairment status (cross-validated AUC = 0.8210). Moreover, apathy and psychosis exhibited lower onset hazards compared to affective and hyperactivity, with age, sex, education, and *APOE* ε4 status independently predicting NPSs onset.

The relationship between NPSs and cognition in AD is multifaceted, and prior evidence on whether NPSs accelerate cognitive decline has been inconsistent (Becker et al., 2018; Craig et al., 2005; Creese & Ismail, 2022; Rabl et al., 2024). To address this, our study extends earlier work by assessing global and domain-specific cognition together with a comprehensive, severity-graded assessment of the 12-item NPI grouped into four subsyndromes. In our discovery cohort characterized by lower education and more advanced disease, baseline NPSs burden was significantly associated with global cognition and memory, with these associations observed specifically for the hyperactive, affective, and apathy subsyndromes. Notably, the ADNI cohort extended these findings, demonstrating that NPSs burden and all four subsyndromes were associated with a broader range of cognitive domains, including global cognition, memory, language, and executive functions. This suggests that cohort factors, particularly cognitive reserve, may attenuate detectable links between NPSs and complex cognitive domains such as executive and language functions, underscoring the importance of accounting for population heterogeneity in study design and interpretation.

Our findings indicate that NPSs, particularly hyperactivity, affective, and apathy subsyndromes, are associated with elevated plasma NFL levels, an association that persisted after multivariable adjustment. This reinforces an independent association between NPSs and axonal injury. Our findings align with prior cross-sectional studies linking plasma NFL to specific neuropsychiatric domains, including aberrant motor behavior, anxiety, depression, sleep disturbance, disinhibition, and euphoria (Huang et al., 2024; Ikanga et al., 2025). In addition, longitudinal evidence also indicates that baseline apathy, anxiety, or depression predicts increasing NFL over time (Kang et al., 2025). However, we did not observe such associations in the present study, warranting further investigation. Furthermore, a recent investigation identified that plasma NFL partially mediated the association between sleep disturbances (assessed via the NPI sleep subscale) and cognition (Guo et al., 2025). Similarly, we also observed that plasma NFL mediated the associations between NPSs and cognition. Together, these observations strengthen the evidence that plasma NFL reflects NPS-related axonal injury in AD.

MRI-evident gray-matter atrophy is a hallmark of AD and predicts earlier, steeper cognitive decline. While prior studies have frequently described NPSs in relation to frontal, cingulate, insular, and temporal areas (Ramirez et al., 2025), we instead focused on early AD-vulnerable brain regions, including the hippocampus, middle temporal, entorhinal cortex, and fusiform gyrus, to directly investigate whether NPSs are linked to AD-specific neurodegenerative topography. Existing evidence supports such neuroanatomical associations, for instance, temporal lobe atrophy with apathy (Huey et al., 2017; Mohamed Nour, Jiao, & Teng, 2021), affective (Hu et al., 2015), depression (Mohamed Nour et al., 2021), and delusions; fusiform gyrus atrophy with affective (Hu et al., 2015) and agitation (Huey et al., 2017); hippocampal atrophy with apathy, anxiety, depression (Mohamed Nour et al., 2021), agitation (Trzepacz et al., 2013), and delusions (Serra et al., 2010); and entorhinal cortex atrophy with affective symptoms (Hu et al., 2015). Our neuroimaging analyses demonstrated that global NPSs burden and hyperactivity, apathy, and affective subsyndromes showed several suggestive or significant associations with these AD-vulnerable regional atrophy in both cohorts. These findings suggest that region-specific atrophy may serve as a useful imaging marker for understanding the neurobiology of NPSs in AD, warranting further investigation.

The mechanisms linking NPSs to cognitive impairment are multifactorial. NPSs may exacerbate amyloid plaque deposition, as evidenced by greater amyloid burden in symptomatic individuals (Marshall et al., 2013; Mori et al., 2014) and by preclinical models linking depressive-like behavior to increased plaque formation (Liu et al., 2025). Impairment of glymphatic clearance, a key pathway for metabolic waste removal observed in generalized anxiety disorder, could further contribute to amyloid accumulation (Qian et al., 2025). Second, NPSs may amplify oxidative stress (Cecerska-Heryć et al., 2022), contributing to neuronal damage that could be mitigated by targeted intervention. Furthermore, sustained NPSs might promote a chronic pro-inflammatory state, thereby accelerating AD progression. This hypothesis is consistent with multiple studies connecting NPSs to dysregulation of inflammatory markers and immune cell profiles (Clark et al., 2022; Liu et al., 2025; Schaffer Aguzzoli et al., 2023).

Based on the above findings, we hypothesize that NPSs may be associated with neuroinflammation (Schaffer Aguzzoli et al., 2023), neuronal injury, progressive atrophy, and cognitive decline (Zetterberg et al., 2016). Our cross-sectional sequential mediation analyses in both cohorts were consistent with a pathway linking NPSs to cognition via plasma NFL and brain atrophy, particularly evident for the apathy and affective subsyndromes. This aligns with the view that NPSs amplify neuroaxonal injury (Huang et al., 2024; Kang et al., 2025) and brain atrophy (Zetterberg et al., 2016). Furthermore, LOESS and CLPM analyses suggested early NPSs emergence (Wise et al., 2019) and supported the first two steps of the proposed pathway. Longitudinally, the proposed pathway remained significant for apathy and affective subsyndromes, providing partial, subsyndrome-specific support. Although baseline NPSs did not predict changes in NFL or atrophy, these analyses address a different question from our longitudinal mediation model, which examined whether baseline levels of NPSs, NFL, and atrophy predict cognitive decline slope. Therefore, these two findings are not contradictory. Nevertheless, larger studies are needed to further validate these pathways. Importantly, cross-sectional findings should be interpreted as consistent with a hypothetical sequence, not as proof of causality or temporal precedence.

Having observed the sequential mediation pathway, we next examined the clinical utility of its key components. Adding NPSs and NFL significantly outperformed the reference model in identifying concurrent cognitive status, further supporting the clinical relevance of these variables. Among subsyndromes, hyperactivity contributed most strongly to discrimination, followed by affective, apathy, and psychosis. These findings suggest that clinical symptoms and fluid biomarkers may capture distinct dimensions of disease heterogeneity, such as neurobehavioral vulnerability and neuroaxonal injury, beyond demographic and genetic factors. External validation is warranted, but the minimal overfitting (optimism = 0.0018) supports the robustness of these findings.

Extending our main findings on subsyndrome heterogeneity, we explored onset patterns after excluding baseline cases. Apathy and psychosis showed significantly lower onset hazards than affective and hyperactivity, consistent with their later emergence in AD. This aligns with postmortem evidence linking earlier Braak stages to hyperactivity and affective subsyndromes (e.g. agitation, anxiety, and depression) and later stages to psychosis (e.g. delusion) (Ehrenberg et al., 2018). Our study also identified that old age, female sex, lower education (Chang et al., 2020), and *APOE* ε4 carriage (Vattathil et al., 2025) were independently associated with higher NPS onset, with *APOE* ε4 carriers conferring the strongest effect. These subsyndrome-specific onset patterns may inform future research on stage-targeted risk stratification.

This study advances prior research through stepwise dual-cohort validation, longitudinal design, multi-biomarker integration, and sequential mediation analysis. By concurrently assessing global NPSs burden, distinct subsyndromes, and graded severity, we confirmed broad NPS–AD associations and delineated severity-specific profiles. Nevertheless, several limitations should be noted. First, the NPI-Q relies on caregiver reports, and NPSs clustering varies across studies due to differences in disease stage, population characteristics, and cultural context, underscoring the need for more standardized approaches in future research. Second, while our sequential mediation models were consistent with the proposed directional pathway, the primary evidence remains cross-sectional, and longitudinal analyses provided only partial support. Bidirectional relationships cannot be fully excluded, although our LOESS analyses and reverse pathway (Supplementary Tables S17 and S18) suggested that the forward direction is more plausible. Third, the relatively low proportion of participants with severe NPSs may constrain generalizability to such populations, necessitating validation in cohorts with a broader severity spectrum. Fourth, although we excluded antidepressant/antipsychotic users at baseline, residual confounding from cognitive-enhancing medications (e.g. cholinesterase inhibitors, memantine) cannot be fully ruled out, warranting future studies with detailed medication data.

## Conclusion

In summary, NPSs are strongly associated with cognitive performance in AD, with plasma NFL and brain atrophy potentially linking NPSs to cognition. NPSs and NFL showed good discriminative ability for cognitive impairment, whereas apathy and psychosis exhibited lower onset hazards than affective and hyperactivity. These findings suggest that NPSs and neuroaxonal injury may be promising targets for future mechanistic and interventional studies in AD, warranting further prospective validation.

## Supporting information

10.1017/S003329172610484X.sm001 Wang et al. supplementary material Wang et al. supplementary material

## Data Availability

The ADNI data used here were from https://adni.loni.usc.edu/. The data from the discovery cohort are available from the corresponding author upon reasonable request.

## Supplementary material

The supplementary material for this article can be found at http://doi.org/10.1017/S003329172610484X.

## Figure 1. Long description

At the top left, the ADNI Cohort panel details participant selection, exclusions, and final sample size, with a breakdown by cognitive status (C N, M C I, A D) and follow-up. Neuropsychiatric symptoms are visualized as a word cloud, with depression and irritability prominent. Biomarkers listed include plasma N F L and A P O E epsilon 4, and structural M R I regions. Cognitive function is assessed by M M S E, A D A S 13, C D R S B, and domain-specific tests. The top right Discovery Cohort panel shows smaller groups (C N, M C I, A D), neuropsychiatric symptom prevalence, and similar biomarker and cognitive measures. A red arrow labeled Verification connects the Discovery Cohort to the central right panel. The middle row contains three panels: left, violin plots show plasma N F L versus neuropsychiatric symptom burden across models; center, a schematic links neuropsychiatric symptoms to A D biomarkers and cognitive decline over time; right, a path diagram illustrates cross-sectional associations among neuropsychiatric symptoms, N F L, regions of interest, and cognition, with standardized coefficients. The bottom row has three panels: left, a mediation diagram shows neuropsychiatric symptoms affecting cognition via plasma N F L and brain atrophy; center, a table and R O C curve display model discrimination of cognitive status; right, a survival plot shows onset patterns of neuropsychiatric symptom subsyndromes over time, with log-rank P equals 0.016.

Navigate back to Figure 1..

## Table 1. Long description

The table presents rows for age, sex, education, A P O E epsilon 4 carrier status, lifestyle factors, comorbidities, cognitive scores, biomarkers, and diagnostic categories. Columns are grouped by N P S domains: global burden, hyperactivity, psychosis, affective, and apathy, each subdivided into none, mild, and severe. For age, means range from 72.96 to 75.15 years. Male percentage is highest in severe global N P S at 73.27 percent. A P O E epsilon 4 carriers increase with N P S severity, up to 63.37 percent in severe global N P S. Smoking rates are similar across groups, while drinking is low overall. Hypertension prevalence ranges from 41.91 to 55.45 percent. Stroke is rare, highest in severe global N P S at 4.95 percent. Cognitive scores (M M S E, A D A S 13, C D R S B, M E M, L A N, E F) worsen with increasing N P S severity. N F L levels and A D signature R O I values are also provided. Diagnostic breakdowns (C N, M C I, A D) and follow-up numbers and times are listed for each group. Superscript a and b indicate significant differences versus none or mild N P S.

Navigate back to Table 1..

## Figure 2. Long description

Top-left panel I contains twelve violin plots in a 3 by 4 grid. Each plot shows plasma N F L (log-transformed) on the y-axis and severity of neuropsychiatric symptoms, hyperactivity, psychosis, affective symptoms, or apathy on the x-axis. Regression lines and p-values are displayed above each plot, with significant associations highlighted. Top-right panel II is a semi-circular heatmap with coefficient values mapped by color from red (positive) to blue (negative). The inner arc lists cognitive and brain structure measures: C D R S B, A D A S 13, M M S E, MEM, E F, L A N, hippocampus, entorhinal, putamen, and mid-temporal. Each segment shows the standardized coefficient for the association with baseline neuropsychiatric symptoms. Bottom-left panel III contains fifteen triangular mediation diagrams labeled A to O. Each triangle connects N P S s, N F L, region of interest (R O I), and cognition (M M S E, Mo C A, C D R, MEM). Solid and dotted arrows indicate significant and non-significant indirect pathways, with standardized coefficients and p-values annotated. Bottom-right panel IV has four subpanels: A is a schematic of mediation pathways linking N P S s, plasma N F L, A D-signature R O I, and cognition; B, C, and D are circular lollipop charts for N P S s, affective, and apathy domains, respectively. Each chart displays standardized coefficients for mediation pathways, with vertical lines representing effect size and circle color indicating direction (red for positive, blue for negative). Asterisks denote raw p-value significance.

Navigate back to Figure 2..

## Figure 3. Long description

At the top, section I displays fifteen line graphs in three rows and five columns. Each graph plots cognitive scores (MMSE, ADAS13, or CDRSB) on the y-axis against time in years on the x-axis. The first row (A–E) shows MMSE decline over time for different symptom groups: hyperactivity, psychosis, affective, and apathy, with interaction p-values less than 0.001. The second row (F–J) shows ADAS13 increase over time for the same symptom groups. The third row (K–O) shows CDRSB increase over time for the same groups. Each graph includes three colored lines (red, black, blue) representing subgroups, with shaded confidence intervals. Section II, below, starts at the top left with a flowchart (A) showing N P S s affecting plasma N F L and A D-signature R O I, both leading to slope of cognition. Below, a table lists indirect effect pathways: I E, I E 1, I E 2, I E 3, with corresponding mediation routes. To the right, three radial lollipop charts (B, C, D) display standardized coefficients (S C) for N P S s, affective, and apathy domains, respectively. Each chart is divided into four quadrants labeled E F, L A N, M E M, and MMSE/ADAS13/CDRSB. Red circles indicate positive associations, blue circles negative, with S C values on the radius. Asterisks mark significance levels.

Navigate back to Figure 3..

## Figure 4. Long description

The table lists eight models in the leftmost column: R E F, R E F plus Hyperactivity, R E F plus Psychosis, R E F plus Affective, R E F plus Apathy, R E F plus N P I- Q Score, R E F plus N F L, and R E F plus N P I -Q Score plus N F L. The next column shows A U C values with 95 percent confidence intervals: R E F 0.6754 [0.6389, 0.7120], R E F plus Hyperactivity 0.7377 [0.7175, 0.7578], R E F plus Psychosis 0.6913 [0.6729, 0.7096], R E F plus Affective 0.7278 [0.7058, 0.7498], R E F plus Apathy 0.7136 [0.6761, 0.7512], R E F plus N P I -Q Score 0.7580 [0.7397, 0.7763], R E F plus N F L 0.7648 [0.7339, 0.7957], R E F plus N P I -Q Score plus N F L 0.8210 [0.8025, 0.8395]. The T Value column lists corresponding values: 9.4188, 23.1129, 20.4437, 20.3164, 11.1589, 27.6345, 16.8076, 33.9980. The P Value column shows all values as less than 0.0001. To the right, a horizontal forest plot aligns with each model row, showing blue squares for A U C point estimates and horizontal error bars for 95 percent confidence intervals. A vertical dashed line marks A U C equals 0.6. The rightmost model, R E F plus N P I -Q Score plus N F L, shows the highest A U C and the furthest rightward square. All models have A U C values and confidence intervals above 0.5, indicating discriminative ability.

Navigate back to Figure 4..

## Figure 5. Long description

Panel A at top left is a Kaplan-Meier line graph with x-axis labeled Time (years) from 0 to 14 and y-axis labeled Survival Probability from 0 to 1. Four colored lines represent Affective (blue), Hyperactivity (red), Apathy (green), and Psychosis (teal). The Affective line remains highest, indicating later onset, while Psychosis and Apathy decline more rapidly. Log-rank P equals 0.016. Below, a table shows the number at risk for each subsyndrome at intervals. Panel B at bottom left is a forest plot with y-axis listing Neuropsychiatric Subsyndromes (Apathy, Psychosis, Hyperactivity) and x-axis labeled Hazard Ratio (H R) from 0.4 to 1.4. Affective is the reference (H R equals 1.00). Apathy (H R 0.65, 95 percent C I 0.53 to 0.79) and Psychosis (H R 0.66, 95 percent C I 0.54 to 0.80) show significantly lower risk compared to Affective, while Hyperactivity (H R 0.99, 95 percent C I 0.83 to 1.19) is not significant. Panel C at bottom right contains four cumulative incidence function plots, one for each subsyndrome. Each plot has x-axis Time (Year) from 0 to 20 and y-axis Cumulative Incidence from 0 to 0.8. Blue lines (Target N P S s) rise more steeply than red lines (Other N P S s) for all subsyndromes. Gray's test P is less than 0.001 for both events.

Navigate back to Figure 5..
