# Supplementary material for: Plasma neurofilament light chain and regional brain atrophy mediate the association of neuropsychiatric symptoms with cognition in Alzheimer’s disease: evidence from two population-based studies
Source: Psychol Med. 2026 Jun 15;56:e192. doi: 10.1017/S003329172610484X (PMC13280697; doi:10.1017/S003329172610484X)
Supplement: Wang et al. supplementary material [file S003329172610484Xsup001.docx]

**Supplementary material**

| Content | page |
| --- | --- |
| eMethods | 2 |
| Table S1. Baseline demographic characteristics of participants in discovery cohort | 3 |
| Table S2. Cross-sectional associations of NPSs with plasma NFL, cognition, and brain MRI in discovery cohort with model 1 | 4 |
| Table S3. Cross-sectional associations of NPSs with plasma biomarkers, cognition and brain MRI in ADNI with model 1 | 5 |
| Table S4. Cross-sectional associations of NPSs with plasma NFL, cognition, and brain MRI in discovery cohort with model 2 | 6 |
| Table S5. Cross-sectional associations of NPSs with plasma biomarkers, cognition and brain MRI in ADNI with model 2 | 7 |
| Table S6. Cross-sectional associations of NPSs with plasma NFL, cognition, and brain MRI in discovery cohort with model 3 | 8 |
| Table S7. Cross-sectional associations of NPSs with plasma biomarkers, cognition and brain MRI in ADNI with model 3 | 9 |
| Table S8. Cross-lagged panel model results examining temporal relationships from NPSs, plasma NFL, brain atrophy, and cognitive decline | 10 |
| Table S9. Sequential mediation models linking NPSs to cognition in the overall ADNI cohort | 11 |
| Table S10. Sequential mediation models linking NPSs to cognition in the ADNI female subsample | 12 |
| Table S11. Sequential mediation models linking NPSs to cognition in the ADNI male subsample | 13 |
| Table S12. Sequential mediation models linking NPSs to the slope of cognition in the overall ADNI cohort | 14 |
| Table S13. Sequential mediation models linking NPSs to the slope of cognition in the ADNI female subsample | 15 |
| Table S14. Sequential mediation models linking NPSs to the slope of cognition in the ADNI male subsample | 16 |
| Table S15. Discriminative performance of models for concurrent cognitive status | 17 |
| Table S16. Time-dependent Cox proportional hazards model for NPSs subsyndrome onset | 18 |
| Table S17. Sequential mediation models for the reverse direction (atrophy → NPSs → NFL → cognition) in the ADNI cohort | 19 |
| Table S17. Sequential mediation models for the reverse direction (atrophy → NPSs → NFL → slope of cognition) in the ADNI cohort | 20 |
| Figure S1. Mediation effects of brain structures on the associations between NPSs and cognition in the validation cohort | 21 |
| Figure S2. LOESS trajectories of NPSs, plasma NFL, and brain atrophy across AD clinical stages | 22 |
| References | 23 |

**eMethods**

**Inclusion and exclusion criteria in verification cohort**

Participants were diagnosed with mild cognitive impairment (MCI) if they met the following criteria: (1) self-reported cognitive complaints by the participant or an informant; (2) Montreal Cognitive Assessment (MoCA) scores (Lu et al., 2011) below established education-stratified cutoffs (illiterate: ≤13; 1–6 years of education: ≤19; ≥7 years: ≤24); (3) a Clinical Dementia Rating (CDR) score of 0.5; (4) a Hachinski Ischemic Scale score < 4; and (5) preserved activities of daily living. Probable Alzheimer’s disease dementia (AD) was diagnosed according to the *Diagnostic and Statistical Manual of Mental Disorders*, *Fourth Edition* (DSM-IV) criteria (Gmitrowicz & Kucharska, 1994), requiring a CDR score of 1 or 2.

Cognitively normal persons (CNs) were required to meet the following criteria: (1) no self- or informant-reported cognitive complaints and a MoCA score within the education-adjusted normal range; and (2) no history of major systemic diseases (including significant cardiac, hepatic, renal, or pulmonary disorders) or tumor-related illnesses.

Exclusion criteria included: 1) history of tumors, brain trauma, or intracranial surgery; 2) cognitive impairment secondary to other neuropsychiatric disorders (e.g., frontotemporal dementia, dementia with Lewy bodies, Parkinson’s disease, encephalitis, epilepsy and other neurological illness); 3) systemic diseases (e.g., thyroid dysfunction, syphilis, and HIV); 4) severe dementia preventing cooperation with MRI; 5) large acute infarction area (diameter > 2 cm); 6) WMHs (Fazekas grade ≥ 2), lacunes, CMBs or PVS; 7) inadequate MRI quality; 8) severe visuospatial deficits, hearing impairments, or language disorders; in vivo dentures or metallic stents; and 9) use of antidepressant or antipsychotic medications.

**Cognitive assessment**

ADNI_MEM was obtained by recording some items. These items included RVLT (Trial 1, Trial 2, Trial 3, Trial 4, Trial 5, Interference, Immediate recall, 30 minutes delay and Recognition), ADAS-Cog (Trial 1, Trial2, Trial 3, Recall, Recognition present and Recognition absent), Logical Memory (Immediate and Delay) and MMSE (Ball recall, Flag recall and Tree recall).

ADNI_LAN measures encompass relevant items of Neuropsychological Battery (Category Fluency-Animals, Category Fluency-Vegetables and Boston Naming), ADAS-Cognitive Behavior (Following Commands, Object Naming and Ideational Practice), MMSE (Naming an Object-Watch, Naming an Object-Pencil, Repeating a Sentence, Reading a Sentence, Writing a Sentence, Following a Series of Instructions) and MoCA (Letter F Fluency, Animal Naming-Lion, Camel, Rhino and Sentence Repetition).

ADNI_EF measures encompassed Category Fluency-animals, Category Fluency-vegetables, Trails A and B, Digit span backwards, WAIS-R Digit Symbol Substitution, and 5 Clock Drawing items (circle, symbol, numbers, hands, time).

In the discovery cohort, all neuropsychological assessments were administered by trained technicians within a single day per participant. Memory (MEM) was evaluated with the Auditory Verbal Learning Test (AVLT). Language function was assessed using the animal category of the Verbal Fluency Test (AFT) and Boston Naming (BNT). Executive functions (EF) were measured with the Trail Making Test‐Part B (TMT‐B) and the Stroop Color‐Word Test‐C (SCWT‐C). As each cognitive domain was evaluated by multiple tests, a composite z‑score was derived for each domain by averaging the z‑scores of the tests for that domain.

**Table S1. Baseline demographic characteristics of participants in the discovery cohort.**

|  | NPSs (Global burden) | | |  | Hyperactivity | | |  | Psychosis | | |  | Affective | | |  | Apathy | | |
| --- | --- | --- | --- | --- | --- | --- | --- | --- | --- | --- | --- | --- | --- | --- | --- | --- | --- | --- | --- |
|  | none | mild | severe |  | none | mild | severe |  | none | mild | severe |  | none | mild | severe |  | none | mild | severe |
| Age (y) | 64.54(8.82) | 66.62(8.78) | 68.78(7.32) **^a^** |  | 66.14(8.51) | 69.71(8.30) | 65.50(7.53) |  | 65.63(9.13) | 68.08(7.33) | 69.50(4.81) |  | 65.77(8.55) | 67.95(8.25) | 69.17(7.77) |  | 65.93(8.52) | 69.62(8.16) | 65.92(7.53) |
| Male ( n, %) | 36(72.00) | 20(44.44) **^a^** | 32(62.75) |  | 64(59.26) | 12(50.00) | 12(85.71) |  | 56(62.92) | 25(51.02) | 7(87.50) |  | 57(61.29) | 22(53.66) | 9(75.00) |  | 64(61.54) | 15(51.72) | 9(69.23) |
| Education (y) | 8.80(4.38) | 9.44(4.33) | 7.76(4.37) |  | 8.83(4.35) | 7.88(4.74) | 8.43(4.18) |  | 8.78(4.46) | 8.61(4.22) | 7.25(4.83) |  | 8.74(4.44) | 8.20(4.37) | 9.33(4.27) |  | 8.53(4.50) | 9.00(4.27) | 8.69(3.99) |
| *APOE*ε4 Carriers (n, %) | 16(32.00) | 17(37.78) | 22(43.14) |  | 38(35.19) | 11(45.83) | 6(42.86) |  | 28(31.46) | 24(48.98) | 3(37.50) |  | 34(36.56) | 16(39.02) | 5(41.67) |  | 40(38.46) | 10(34.48) | 5(38.46) |
| Lifestyles |  |  |  |  |  |  |  |  |  |  |  |  |  |  |  |  |  |  |  |
| Smoking (n, %) | 5(10.00) | 13(28.89) | 10(19.71) |  | 22(20.37) | 4(16.67) | 2(14.29) |  | 16(17.98) | 12(24.49) | 0(0) |  | 18(19.35) | 8(19.51) | 2(16.67) |  | 18(17.31) | 7(24.14) | 3(23.08) |
| Drinking (n, %) | 6(12.00) | 10(22.22) | 15(29.41) |  | 22(20.37) | 5(20.83) | 5(35.71) |  | 18(20.22) | 11(22.45) | 2(25.00) |  | 16(17.20) | 11(26.83) | 4(33.33) |  | 18(17.31) | 7(24.14) | 6(46.15) |
| Medical comorbidities |  |  |  |  |  |  |  |  |  |  |  |  |  |  |  |  |  |  |  |
| Hypertension (n, %) | 22(44.00) | 13(28.89) | 23(45.10) |  | 42(38.89) | 11(45.83) | 5(35.71) |  | 37(41.57) | 16(32.65) | 5(62.50) |  | 39(41.94) | 13(31.71) | 6(50.00) |  | 39(37.50) | 14(48.28) | 5(38.46) |
| DM (n, %) | 10(20.00) | 8(17.78) | 7(13.73) |  | 20(18.52) | 2(8.33) | 3(21.43) |  | 17(19.10) | 5(0) | 3(37.50) |  | 18(19.35) | 6(14.63) | 1(8.33) |  | 16(15.38) | 6(20.69) | 3(23.08) |
| Cognitive function |  |  |  |  |  |  |  |  |  |  |  |  |  |  |  |  |  |  |  |
| MMSE | 23.96(6.12) | 23.16(5.26) | 15.94(6.62) **^a,b^** |  | 22.57(6.35) | 17.88(6.69) **^a^** | 13.36(6.49) **^a^** |  | 21.60(7.09) | 21.10(6.20) **^a^** | 12.12(6.18) **^a^** |  | 22.36(6.38) | 19.76(7.00) **^a^** | 13.67(7.45) **^a,b^** |  | 22.61(6.54) | 17.52(6.58) **^a^** | 14.85(6.24) **^a^** |
| MoCA | 19.06(6.72) | 18.36(6.41) | 10.50(5.72) **^a,b^** |  | 17.63(7.06) | 12.04(6.33) **^a^** | 8.75(4.63) **^a^** |  | 16.62(7.59) | 16.12(6.27) **^a^** | 6.00(4.86) **^a^** |  | 17.39(6.87) | 14.22(7.72) **^a^** | 10.09(6.38) **^a^** |  | 17.62(7.15) | 12.36(6.34) **^a^** | 9.92(5.52) **^a^** |
| CDR | 0.46(0.36) | 0.54(0.40) | 1.08(0.56) **^a,b^** |  | 0.58(0.48) | 0.83(0.38) **^a^** | 1.39(0.56) **^a,b^** |  | 0.66(0.53) | 0.64(0.42) **^a^** | 1.50(0.54) **^a^** |  | 0.60(0.45) | 0.74(0.54) | 1.33(0.62) **^a,b^** |  | 0.56(0.45) | 0.95(0.49) **^a^** | 1.27(0.63) **^a^** |
| MEM | 0.24(0.79) | 0.12(0.98) | -0.70(0.46) **^a,b^** |  | 0.05(0.89) | -0.59(0.61) **^a^** | -0.69(0.42) **^a^** |  | -0.05(0.87) | -0.09(0.89) **^a^** | -0.87(0.41) **^a^** |  | 0.04(0.86) | -0.28(0.89) **^a^** | -0.76(0.51) **^a^** |  | 0.06(0.90) | -0.53(0.56) **^a^** | -0.75(0.56) **^a^** |
| LAN | 0.10(0.86) | 0.03(0.82) | -0.33(1.01) |  | 0.01(0.84) | -0.39(1.13) | -0.16(1.02) |  | -0.04(0.92) | -0.09(0.85) | -0.19(1.21) |  | -0.09(0.93) | 0.01(0.91) | -0.19(0.76) |  | 0.03(0.93) | -0.32(0.89) | -0.33(0.96) |
| EF | -0.24(0.91) | -0.17(0.82) | -0.35(0.68) |  | -0.21(0.86) | -0.43(0.51) | -0.30(0.91) |  | -0.24(0.71) | -0.33(0.79) | 0.28(1.08) |  | -0.28(0.81) | -0.18(0.86) | -0.04(1.10) |  | -0.25(0.84) | -0.08(0.83) | -0.59(0.56) |
| AD-signature ROI volume | 0.34(1.65) | 0.54(1.60) | -1.05(1.61) **^a,b^** |  | 0.27(1.68) | -0.79(1.89) **^a^** | -1.40(0.86) **^a^** |  | 0.07(1.79) | 0.01(1.67) | -1.36(1.62) |  | 0.25(1.53) | 0.03(1.83) **^a^** | -2.29(1.68) **^a^** |  | 0.25(1.71) | -0.60(1.65) | -1.37(1.71) |
| NFL (pg/ml) | 16.58(9.99) | 22.63(12.23) | 36.07(27.97) **^a^** |  | 25.66(24.09) | 25.55(19.60) | 35.77(27.45) |  | 25.92(20.51) | 21.22(11.15) | 51.97(38.13) **^a,b^** |  | 25.44(21.35) | 27.93(20.66) | 35.26(31.24) |  | 23.40(21.83) | 29.21(20.54) | 39.13(26.18) **^a^** |
| CN/MCI/AD (n) | 21/17/12 | 11/21/13 **^a^** | 0/12/39 **^b^** |  | 30/42/36 | 2/7/15 | 0/1/13 **^a^** |  | 25/29/35 | 7/21/21 **^a^** | 0/0/8 **^a^** |  | 36/36/31 | 6/12/23 | 0/2/10**^a^** |  | 31/39/34 | 1/9/19 **^a^** | 0/2/11 **^a^** |

Categorical variables are reported as numbers (percentages); continuous variables are reported as means (SDs). Between-group comparisons were performed using ANOVA or Kruskal-Wallis tests for continuous variables, and chi-square or Fisher's exact tests for categorical variables, with appropriate post-hoc analyses and multiple testing corrections applied. **^a^** p < 0.05 versus none-NPS; **^b^** p < 0.05 versus mild-NPSs. NPSs neuropsychiatric symptoms; NFL neurofilament light chain; *APOE* ε4 apolipoprotein ε4; DM diabetes; MMSE Mini-Mental State Examination; ADAS Alzheimer’s disease Assessment Scale; CDR Clinical Dementia Rating; MEM Memory function; LAN Language; EF Executive function; CN cognitively normal; MCI mild cognitive impairment; AD Alzheimer’s Disease; SD standard deviation.

**Table S2. Cross-sectional associations of NPSs with plasma NFL, cognition, and brain MRI in discovery cohort with model 1.**

|  |  | NPSs | | |  | Hyperactivity | | |  | Psychosis | | |  | Affective | | |  | Apathy | | |  |
| --- | --- | --- | --- | --- | --- | --- | --- | --- | --- | --- | --- | --- | --- | --- | --- | --- | --- | --- | --- | --- | --- |
|  |  | β (95%CI) | P | P_FDR |  | β (95%CI) | P | P_FDR |  | β (95%CI) | P | P_FDR |  | β (95%CI) | P | P_FDR |  | β (95%CI) | P | P_FDR |  |
| NFL |  | 0.1173 (0.0778, 0.1570) | **3.17E-08** | **8.72E-08** |  | 0.0925 (0.0398, 0.1452) | **0.0007** | **0.0015** |  | 0.0758 (0.0167,0.1349) | **0.0123** | 0.1353 |  | 0.0859(0.0322,0.1397) | **0.0019** | **0.0070** |  | 0.1255(0.0079,0.2848) | **3.09E-06** | **8.50E-06** |  |
| MMSE |  | -3.6804 (-4.7953, -2.5655) | **1.15E-09** | **6.33E-06** |  | -5.4766 (-7.6373, -3.3159) | **1.61E-06** | **8.86E-06** |  | -0.9408 (-3.0792 ,1.1976) | 0.3859 | 0.8602 |  | -3.6227 (-5.6773, -1.5681) | **0.0007** | **0.0077** |  | -6.2548 (-8.2745, -4.2351) | **8.75E-09** | **9.63E-08** |  |
| MoCA |  | -3.9617 (-5.1366, -2.7868) | **6.06E-10** | **6.67E-09** |  | -5.8318 (-8.1125, -3.5512) | **1.36E-06** | **1.50E-05** |  | -0.8608 (-3.1245 ,1.4030) | 0.4534 | 0.8602 |  | -3.6000 (-5.7839, -1.4161) | **0.0014** | **0.0077** |  | -6.4091 (-8.5664, -4.2518) | **3.12E-08** | **1.14E-07** |  |
| CDR |  | 0.3011 (0.2098,0.3924) | **1.18E-09** | **4.33E-09** |  | 0.4153 (0.2362 ,0.5945) | **1.00E-05** | **3.67E-05** |  | 0.0643 (-0.1109 ,0.2395) | 0.4692 | 0.8602 |  | 0.2546 (0.0845 ,0.4247) | **0.0036** | **0.0099** |  | 0.4962 (0.3295 ,0.6629) | **2.82E-08** | **1.55E-07** |  |
| MEM |  | -0.3779 (-0.5352, -0.2206) | **5.45E-06** | **1.20E-05** |  | -0.5869 (-0.8823, -0.2915) | **1.39E-04** | **3.82E-04** |  | 0.0572 (-0.2222 ,0.3366) | 0.6859 | 0.8602 |  | -0.3109 (-0.5841, -0.0377) | **0.0261** | **0.0479** |  | -0.6458 (-0.9252, -0.3663) | **1.15E-05** | **2.53E-05** |  |
| LAN |  | -0.1927 (-0.3807, -0.0047) | **0.0446** | 0.0545 |  | -0.2780 (-0.6296 ,0.0737) | 0.1203 | 0.1470 |  | 0.0256 (-0.2951 ,0.3463) | 0.8748 | 0.8602 |  | 0.0870 (-0.2337 ,0.4077) | 0.5924 | 0.6516 |  | -0.3567 (-0.6927, -0.0207) | **0.0376** | **0.0460** |  |
| EF |  | -0.0824 (-0.3665,0.2017) | 0.5634 | 0.5634 |  | -0.1752 (-0.7245 ,0.3741) | 0.5252 | 0.5252 |  | -0.1106 (-0.6094 ,0.3881) | 0.6582 | 0.8602 |  | 0.0862 (-0.4136 ,0.5861) | 0.7307 | 0.7307 |  | 0.0648 (-0.5400 ,0.6696) | 0.8306 | 0.8306 |  |
| Hippocampus |  | -0.3482 (-0.6272, -0.0692) | **0.0149** | **0.0205** |  | -0.6374 (-1.1604, -0.1144) | **0.0173** | **0.0317** |  | 0.0504 (-0.4001 ,0.5009) | 0.8250 | 0.8602 |  | -0.4242 (-0.8748 ,0.0265) | 0.0648 | 0.1018 |  | -0.9300 (-1.4052, -0.4548) | **1.76E-04** | **3.23E-04** |  |
| Entorhinal |  | -0.1619 (-0.3609,0.0371) | 0.1099 | 0.1209 |  | -0.2763 (-0.6496 ,0.0971) | 0.1455 | 0.1601 |  | 0.3052 (-0.0066 ,0.6169) | 0.0550 | 0.3025 |  | -0.4277 (-0.7395, -0.1159) | **0.0076** | **0.0167** |  | -0.2837 (-0.6349 ,0.0675) | 0.1123 | 0.1235 |  |
| Fusiform |  | -0.5312 (-0.9481, -0.1142) | **0.0130** | **0.0204** |  | -0.8205 (-1.6081, -0.0329) | **0.0413** | 0.0649 |  | 0.3182 (-0.3534 ,0.9898) | 0.3499 | 0.8602 |  | -0.4564 (-1.1355 ,0.2227) | 0.1858 | 0.2555 |  | -0.8301 (-1.5700, -0.0901) | **0.0282** | **0.0388** |  |
| Mid temporal |  | -1.0373 (-1.8439, -0.2307) | **0.0122** | **0.0224** |  | -1.4740 (-3.0021 ,0.0542) | 0.0585 | 0.0804 |  | 0.0211 (-1.2836 ,1.3258) | 0.9745 | 0.8602 |  | -0.5707 (-1.8908 ,0.7495) | 0.3937 | 0.4812 |  | -1.9454 (-3.3633, -0.5275) | **0.0076** | **0.0119** |  |

P values were obtained by multiple linear regressions models adjusted for age, sex, years of education, *APOE* ɛ4 status, with further adjustment for intracranial volume for brain structure outcomes. Multiple testing significance was corrected using FDR. Significant at the level of *P* < 0.05 were shown in bold. Abbreviations: NPSs neuropsychiatric symptoms; NFL neurofilament light chain; CI Confidence Interval; FDR false discovery rate; MMSE Mini-Mental State Examination; MoCA Montreal Cognitive Assessment; CDR Clinical Dementia Rating; MEM Memory function; LAN Language; EF Executive function; *APOE* ε4 apolipoprotein ε4.

**Table S3. Cross-sectional associations of NPSs with plasma biomarkers, cognition and brain MRI in ADNI with model 1.**

|  |  | NPSs | | |  | Hyperactivity | | |  | Psychosis | | |  | Affective | | |  | Apathy | | |  |
| --- | --- | --- | --- | --- | --- | --- | --- | --- | --- | --- | --- | --- | --- | --- | --- | --- | --- | --- | --- | --- | --- |
|  |  | β (95%CI) | P | P_FDR |  | β (95%CI) | P | P_FDR |  | β (95%CI) | P | P_FDR |  | β (95%CI) | P | P_FDR |  | β (95%CI) | P | P_FDR |  |
| NFL |  | 0.0446 (0.0101,0.0792) | **0.0115** | **0.0125** |  | 0.0334 (0.0047,0.062) | **0.0225** | **0.0245** |  | 0.0411 (-0.0056,0.0877) | 0.0841 | 0.1262 |  | 0.0609 (0.0228,0.099) | **0.0018** | **0.0024** |  | 0.0527 (0.0103,0.0951) | **0.0151** | **0.0164** |  |
| MMSE |  | -1.1674 (-1.3569, -0.9779) | **3.68E-32** | **1.10E-31** |  | -0.755 (-0.9327, -0.5774) | **1.71E-16** | **5.13E-16** |  | -0.4856 (-0.7056, -0.2656) | **1.59E-05** | **4.77E-05** |  | -0.7591 (-0.9709, -0.5473) | **3.08E-12** | **9.24E-12** |  | -1.156 (-1.3694, -0.9426) | **1.74E-25** | **5.22E-25** |  |
| ADAS13 |  | 4.4292 (3.7421,5.1163) | **6.71E-35** | **2.68E-34** |  | 3.0591 (2.4162,3.7019) | **3.52E-20** | **1.41E-19** |  | 2.0309 (1.2325,2.8293) | **6.76E-07** | **4.06E-06** |  | 3.4618 (2.6973,4.2263) | **1.83E-18** | **7.32E-18** |  | 4.3445 (3.5685,5.1205) | **4.86E-27** | **2.92E-26** |  |
| CDRSB |  | 1.1076 (0.9866,1.2287) | **1.87E-65** | **2.24E-64** |  | 0.8704 (0.7565,0.9843) | **1.85E-47** | **2.22E-46** |  | 0.4412 (0.2942,0.5883) | **4.87E-09** | **5.84E-08** |  | 0.8623 (0.7244,1.0002) | **4.75E-33** | **5.70E-32** |  | 1.1327 (0.9955,1.2699) | **1.64E-54** | **1.97E-53** |  |
| MEM |  | -0.3548 (-0.4067, -0.303) | **5.97E-39** | **3.58E-38** |  | -0.2563 (-0.3048, -0.2078) | **2.26E-24** | **1.36E-23** |  | -0.1458 (-0.2065, -0.0851) | **2.67E-06** | **1.07E-05** |  | -0.282 (-0.3398, -0.2243) | **3.64E-21** | **2.18E-20** |  | -0.323 (-0.3819, -0.2641) | **4.69E-26** | **1.88E-25** |  |
| LAN |  | -0.2539 (-0.3034, -0.2044) | **4.06E-23** | **8.12E-23** |  | -0.1814 (-0.2308, -0.132) | **9.17E-13** | **1.57E-12** |  | -0.1304 (-0.1912, -0.0695) | **2.78E-05** | **6.67E-05** |  | -0.2025 (-0.2611, -0.1439) | **1.72E-11** | **4.13E-11** |  | -0.2633 (-0.323, -0.2036) | **1.25E-17** | **2.50E-17** |  |
| EF |  | -0.2831 (-0.3360, -0.2301) | **7.07E-25** | **1.70E-24** |  | -0.182 (-0.2279, -0.1361) | **1.34E-14** | **3.22E-14** |  | -0.1201 (-0.1767, -0.0634) | **3.45E-05** | **6.90E-05** |  | -0.1819 (-0.2366, -0.1273) | **8.99E-11** | **1.80E-10** |  | -0.2539 (-0.3095, -0.1984) | **8.36E-19** | **2.01E-18** |  |
| Hippocampus |  | -0.0248 (-0.0307, -0.0189) | **3.10E-16** | **5.31E-16** |  | -0.0213 (-0.0268, -0.0158) | **5.92E-14** | **1.18E-13** |  | -0.0082 (-0.0149, -0.0016) | 0.0154 | 0.0264 |  | -0.0156 (-0.0222, -0.009) | **3.79E-06** | **5.69E-06** |  | -0.0213 (-0.0278, -0.0148) | **2.04E-10** | **3.50E-10** |  |
| Entorhinal |  | -0.031 (-0.0396, -0.0224) | **2.37E-12** | **3.56E-12** |  | -0.0219 (-0.0299, -0.0139) | **9.35E-08** | **1.25E-07** |  | -0.0082 (-0.0181,0.0016) | 0.0996 | 0.1328 |  | -0.0243 (-0.0337, -0.0148) | **5.37E-07** | **9.21E-07** |  | -0.0279 (-0.0375, -0.0183) | **1.40E-08** | **2.10E-08** |  |
| Fusiform |  | -0.0147 (-0.0199, -0.0094) | **5.33E-08** | **6.40E-08** |  | -0.009 (-0.0139, -0.0041) | **0.0003** | **0.0004** |  | -0.0021 (-0.008,0.0039) | 0.5027 | 0.6032 |  | -0.0084 (-0.0142, -0.0026) | **0.0046** | **0.0055** |  | -0.0133 (-0.0191, -0.0074) | **9.46E-06** | **1.14E-05** |  |
| Mid temporal |  | -0.016 (-0.0214, -0.0107) | **5.06E-09** | **6.75E-09** |  | -0.0138 (-0.0187, -0.0089) | **4.85E-08** | **7.28E-08** |  | -0.0003 (-0.0064,0.0058) | 0.9215 | 0.9215 |  | 0.0132 (-0.0046,0.031) | 0.1459 | 0.1592 |  | -0.0154 (-0.0213, -0.0094) | **4.65E-07** | **6.20E-07** |  |

P values were obtained by multiple linear regressions models adjusted for age, sex, years of education, *APOE* ɛ4 status, with further adjustment for intracranial volume for brain structure outcomes. Multiple testing significance was corrected using FDR. Significant at the level of *P* < 0.05 were shown in bold. NPSs neuropsychiatric symptoms; NFL neurofilament light chain; ADNI Alzheimer’s Disease Neuroimaging Initiative; CI Confidence Interval; FDR false discovery rate; MMSE Mini-Mental State Examination; ADAS Alzheimer’s disease Assessment Scale; CDRSB Clinical Dementia Rating Sum of Boxes; MEM Memory function; LAN Language; EF Executive function; *APOE* ε4 apolipoprotein ε4.

**Table S4. Cross-sectional associations of NPSs with plasma NFL, cognition, and brain MRI in discovery cohort with model 2.**

|  |  | NPSs | | |  | Hyperactivity | | |  | Psychosis | | |  | Affective | | |  | Apathy | | |  |
| --- | --- | --- | --- | --- | --- | --- | --- | --- | --- | --- | --- | --- | --- | --- | --- | --- | --- | --- | --- | --- | --- |
|  |  | β (95%CI) | P | P_FDR |  | β (95%CI) | P | P_FDR |  | β (95%CI) | P | P_FDR |  | β (95%CI) | P | P_FDR |  | β (95%CI) | P | P_FDR |  |
| NFL |  | 0.1233 (0.0827, 0.1638) | **1.60E-08** | **4.40E-08** |  | 0.0958 (0.0419, 0.1497) | **5.92E-04** | **0.0013** |  | 0.0776(0.0176,0.1376) | **0.0116** | 0.1276 |  | 0.0927(0.0372,0.1481) | **0.0012** | **0.0066** |  | 0.1283(0.0760,0.1805) | **3.29E-06** | **3.62E-06** |  |
| MMSE |  | -3.6402 (-4.7884, -2.4920) | **4.48E-09** | **2.46E-08** |  | -5.4911 (-7.6913, -3.2910) | **2.30E-06** | **1.27E-05** |  | -0.7993 (-2.9699, 1.3713) | 0.4677 | 0.8338 |  | -3.5885 (-5.7104, -1.4665) | **0.0011** | **0.0121** |  | -6.3206 (-8.3534, -4.2877) | **8.15E-09** | **8.97E-08** |  |
| MoCA |  | -3.9532 (-5.1589, -2.7476) | **1.65E-09** | **1.82E-08** |  | -5.8271 (-8.1369, -3.5173) | **1.88E-06** | **2.07E-05** |  | -0.7716 (-3.0714, 1.5282) | 0.5080 | 0.8338 |  | -3.5902 (-5.8419, -1.3384) | **0.0020** | **0.0073** |  | -6.4380 (-8.6059, -4.2702) | **3.30E-08** | **3.63E-07** |  |
| CDR |  | 0.2975 (0.2032,0.3918) | **5.20E-09** | **1.91E-08** |  | 0.4095 (0.2263 ,0.5928) | **2.01E-05** | **7.37E-05** |  | 0.0541 (-0.1240, 0.2323) | 0.5489 | 0.8338 |  | 0.2445 (0.0682 ,0.4207) | **0.0069** | **0.0190** |  | 0.4996 (0.3312 ,0.6680) | **3.21E-08** | **3.53E-07** |  |
| MEM |  | -0.3740 (-0.5368, -0.2111) | **1.31E-05** | **2.88E-05** |  | -0.5803 (-0.8827, -0.2780) | **2.29E-04** | **6.30E-04** |  | 0.0589 (-0.2252, 0.3430) | 0.6822 | 0.8338 |  | -0.3147 (-0.5969, -0.0326) | **0.0291** | 0.0534 |  | -0.6356 (-0.9192, -0.3521) | **2.03E-05** | **2.23E-04** |  |
| LAN |  | -0.2276 (-0.4167, -0.0385) | **0.0187** | **0.0294** |  | -0.3166 (-0.6688 ,0.0355) | 0.0776 | 0.1067 |  | -0.1429 (-0.6602, 0.3744) | 0.5815 | 0.8338 |  | 0.0232 (-0.3027 ,0.3491) | 0.8882 | 0.8882 |  | -0.3311 (-0.6686 ,0.0065) | 0.0545 | 0.0749 |  |
| EF |  | -0.1320 (-0.4259 ,0.1619) | 0.3713 | 0.3713 |  | -0.2443 (-0.8120 ,0.3233) | 0.3914 | 0.3914 |  | -0.0088 (-0.3312, 0.3137) | 0.9572 | 0.9572 |  | 0.0747 (-0.4467 ,0.5961) | 0.7747 | 0.8522 |  | 0.0418 (-0.5797 ,0.6634) | 0.8930 | 0.8930 |  |
| Hippocampus |  | -0.3276 (-0.6146, -0.0407) | **0.0256** | **0.0313** |  | -0.6356 (-1.1651, -0.1061) | **0.0191** | **0.0350** |  | 0.1044 (-0.3531, 0.5620) | 0.6520 | 0.8338 |  | -0.4030 (-0.8699 ,0.0640) | 0.0900 | 0.1414 |  | -0.9335 (-1.4137, -0.4533) | **1.96E-04** | **0.0022** |  |
| Entorhinal |  | -0.1549 (-0.3612 ,0.0514) | 0.1397 | 0.1537 |  | -0.2647 (-0.6468 ,0.1174) | 0.1726 | 0.1899 |  | 0.3212 (0.0017, 0.6408) | **0.0488** | 0.5368 |  | -0.4272 (-0.7534, -0.1011) | **0.0107** | **0.0235** |  | -0.2918 (-0.6505 ,0.0669) | 0.1098 | 0.1208 |  |
| Fusiform |  | -0.5134 (-0.9388, -0.0881) | **0.0184** | **0.0337** |  | -0.8008 (-1.5932, -0.0084) | **0.0477** | 0.0750 |  | 0.3458 (-0.3317, 1.023) | 0.3140 | 0.8338 |  | -0.5091 (-1.2055 ,0.1873) | 0.1502 | 0.2065 |  | -0.7776 (-1.5229, -0.0322) | **0.0410** | 0.0564 |  |
| Mid temporal |  | -0.9835 (-1.8028, -0.1643) | **0.0191** | **0.0263** |  | -1.3746 (-2.9060 ,0.1569) | 0.0781 | 0.0955 |  | 0.0651 (-1.2454, 1.3756) | 0.9217 | 0.9572 |  | -0.5964 (-1.9453 ,0.7525) | 0.3829 | 0.4680 |  | -1.8732 (-3.2929, -0.4534) | **0.0102** | **0.0160** |  |

P values were obtained by multiple linear regressions models adjusted for age, sex, years of education, *APOE* ɛ4 status, smoking, alcohol use, and history of hypertension and diabetes, with further adjustment for intracranial volume for brain structure outcomes. Multiple testing significance was corrected using FDR. Significant at the level of *P* < 0.05 were shown in bold. NPSs neuropsychiatric symptoms; NFL neurofilament light chain; CI Confidence Interval; FDR false discovery rate; MMSE Mini-Mental State Examination; MoCA Montreal Cognitive Assessment; CDR Clinical Dementia Rating; MEM Memory function; LAN Language; EF Executive function; *APOE* ε4 apolipoprotein ε4.

**Table S5. Cross-sectional associations of NPSs with plasma biomarkers, cognition and brain MRI in ADNI with model 2.**

|  |  | NPSs | | |  | Hyperactivity | | |  | Psychosis | | |  | Affective | | |  | Apathy | | |  |
| --- | --- | --- | --- | --- | --- | --- | --- | --- | --- | --- | --- | --- | --- | --- | --- | --- | --- | --- | --- | --- | --- |
|  |  | β (95%CI) | P | P_FDR |  | β (95%CI) | P | P_FDR |  | β (95%CI) | P | P_FDR |  | β (95%CI) | P | P_FDR |  | β (95%CI) | P | P_FDR |  |
| NFL |  | 0.0478 (0.0128,0.0827) | **0.0075** | **0.0082** |  | 0.0350 (0.0065,0.0635) | **0.0162** | **0.0194** |  | 0.0229 (-0.0254,0.0711) | 0.3519 | 0.5279 |  | 0.0717 (0.0327,0.1107) | **0.0003** | **0.0005** |  | 0.0616 (0.0183,0.1049) | **0.0054** | **0.0059** |  |
| MMSE |  | -1.1131 (-1.3379, -0.8884) | **1.71E-21** | **5.13E-21** |  | -0.7178 (-0.9224, -0.5132) | **9.70E-12** | **2.91E-11** |  | -0.5027 (-0.7608, -0.2446) | **0.0001** | **0.0004** |  | -0.7619 (-1.0075, -0.5162) | **1.59E-09** | **4.77E-09** |  | -1.1336 (-1.3902, -0.8769) | **1.53E-17** | **4.59E-17** |  |
| ADAS13 |  | 4.3717 (3.5516,5.1918) | **1.70E-24** | **6.80E-24** |  | 2.9452 (2.1987,3.6916) | **2.18E-14** | **8.72E-14** |  | 2.1256 (1.1813,3.0699) | **1.10E-05** | **6.60E-05** |  | 3.458 (2.5626,4.3534) | **7.33E-14** | **2.93E-13** |  | 4.589 (3.6512,5.5267) | **4.93E-21** | **2.96E-20** |  |
| CDRSB |  | 1.0728 (0.9274,1.2182) | **1.09E-43** | **1.31E-42** |  | 0.8135 (0.6804,0.9467) | **2.92E-31** | **3.50E-30** |  | 0.4824 (0.3089,0.6559) | **6.02E-08** | **7.22E-07** |  | 0.8506 (0.6891,1.0121) | **5.52E-24** | **6.62E-23** |  | 1.1253 (0.9584,1.2923) | **3.04E-37** | **3.65E-36** |  |
| MEM |  | -0.3485 (-0.4088, -0.2881) | **2.94E-28** | **1.76E-27** |  | -0.2423 (-0.2974, -0.1873) | **1.99E-17** | **1.19E-16** |  | -0.1441 (-0.2143, -0.0739) | **0.0001** | **0.0003** |  | -0.2666 (-0.3327, -0.2004) | **6.28E-15** | **3.77E-14** |  | -0.3359 (-0.4054, -0.2665) | **1.29E-20** | **5.16E-20** |  |
| LAN |  | -0.2387 (-0.2969, -0.1805) | **2.06E-15** | **4.12E-15** |  | -0.1718 (-0.2242, -0.1193) | **1.93E-10** | **4.63E-10** |  | -0.1191 (-0.1852, -0.0531) | **0.0004** | **0.0008** |  | -0.1649 (-0.2280, -0.1018) | **3.47E-07** | **6.94E-06** |  | -0.2403 (-0.3066, -0.174) | **2.04E-12** | **4.90E-12** |  |
| EF |  | -0.2608 (-0.3226, -0.199) | **3.49E-16** | **8.38E-16** |  | -0.158 (-0.2141, -0.102) | **3.99E-08** | **6.84E-08** |  | -0.1315 (-0.2018, -0.0613) | **0.0003** | **0.0007** |  | -0.1787 (-0.2458, -0.1116) | **2.08E-07** | **4.99E-07** |  | -0.2345 (-0.3053, -0.1637) | **1.21E-10** | **2.42E-10** |  |
| Hippocampus |  | -0.0222 (-0.0292, -0.0153) | **4.99E-10** | **8.55E-10** |  | -0.0192 (-0.0255, -0.013) | **2.40E-09** | **4.80E-09** |  | -0.0076 (-0.0153,0.0001) | 0.0530 | 0.0909 |  | -0.0124 (-0.0200, -0.0048) | **0.0014** | **0.0017** |  | -0.0197 (-0.0275, -0.0118) | **9.43E-07** | **1.41E-06** |  |
| Entorhinal |  | -0.0252 (-0.0351, -0.0153) | **6.45E-07** | **9.68E-07** |  | -0.0172 (-0.026, -0.0084) | **0.0001** | **1.50E-04** |  | -0.0039 (-0.0152,0.0073) | 0.4945 | 0.6593 |  | -0.0209 (-0.0316, -0.0102) | **0.0001** | **1.71E-4** |  | -0.0286 (-0.0398, -0.0174) | **6.10E-07** | **1.05E-06** |  |
| Fusiform |  | -0.0108 (-0.0171, -0.0045) | **0.0007** | **8.40E-04** |  | -0.0056 (-0.0112,0) | **0.0496** | 0.0541 |  | 0.0004 (-0.0067,0.0075) | 0.9145 | 0.9976 |  | -0.007 (-0.0137, -0.0002) | **0.0440** | **0.048** |  | -0.0109 (-0.0180, -0.0039) | **0.0025** | **0.0030** |  |
| Mid temporal |  | -0.0123 (-0.0187, -0.006) | **0.0001** | **1.33E-04** |  | -0.0111 (-0.0168, -0.0055) | **0.0001** | **1.33E-04** |  | 0.0022 (-0.005,0.0094) | 0.5537 | 0.6644 |  | -0.0121 (-0.0189, -0.0052) | **0.0006** | **0.0008** |  | -0.0127 (-0.0199, -0.0055) | **0.0006** | **0.0008** |  |

P values were obtained by multiple linear regressions models adjusted for age, sex, years of education, *APOE* ɛ4 status, smoking, alcohol use, and history of hypertension and stroke, with further adjustment for intracranial volume for brain structure outcomes. Multiple testing significance was corrected using FDR. Significant at the level of *P* < 0.05 were shown in bold. NPSs neuropsychiatric symptoms; NFL neurofilament light chain; ADNI Alzheimer’s Disease Neuroimaging Initiative; CI Confidence Interval; FDR false discovery rate; MMSE Mini-Mental State Examination; ADAS Alzheimer’s disease Assessment Scale; CDRSB Clinical Dementia Rating Sum of Boxes; MEM Memory function; LAN Language; EF Executive function; *APOE* ε4 apolipoprotein ε4.

**Table S6. Cross-sectional associations of NPSs with plasma NFL, cognition, and brain MRI in discovery cohort with model 3.**

|  |  | NPSs | | |  | Hyperactivity | | |  | Psychosis | | |  | Affective | | |  | Apathy | | |  |
| --- | --- | --- | --- | --- | --- | --- | --- | --- | --- | --- | --- | --- | --- | --- | --- | --- | --- | --- | --- | --- | --- |
|  |  | β (95%CI) | P | P_FDR |  | β (95%CI) | P | P_FDR |  | β (95%CI) | P | P_FDR |  | β (95%CI) | P | P_FDR |  | β (95%CI) | P | P_FDR |  |
| NFL |  | 0.0919 (0.0500, 0.1337) | **2.73E-05** | **0.000137** |  | 0.0834 (0.0075, 0.1593) | **0.0315** | 0.0524 |  | 0.0198 (-0.0497, 0.0894) | 0.5741 | 0.5741 |  | 0.0674 (-0.0017, 0.1365) | 0.0557 | 0.0696 |  | 0.1256 (0.0515, 0.1998) | **0.0010** | **0.0026** |  |
| MMSE |  | -2.4250 (-3.5192, -1.3309) | **0.000023** | **5.75E-05** |  | -4.0956 (-5.9943, -2.1969) | **3.67E-05** | **6.12E-05** |  | -0.0183 (-1.8409, 1.8043) | 0.9842 | 0.9842 |  | -2.5002 (-4.2834, -0.7169) | **0.0063** | **0.0079** |  | -4.3705 (-6.2497, -2.4914) | **9.48E-06** | **4.74E-05** |  |
| MoCA |  | -2.3972 (-3.5118, -1.2825) | **3.93E-05** | **0.000075** |  | -4.1748 (-6.0801, -2.2694) | **2.86E-05** | **0.000075** |  | 0.2138 (-1.6170, 2.0446) | 0.8177 | 0.8177 |  | -2.2699 (-4.0713, -0.4685) | **0.0139** | **0.0174** |  | -4.0892 (-6.0065, -2.1720) | **0.000045** | **0.000075** |  |
| CDR |  | 0.2058 (0.1147, 0.2969) | **1.63E-05** | **5.61E-05** |  | 0.3071 (0.1467, 0.4675) | **0.0002** | **0.0004** |  | -0.0078 (-0.1599, 0.1443) | 0.9195 | 0.9195 |  | 0.1662 (0.0159, 0.3165) | **0.0305** | **0.0381** |  | 0.3501 (0.1924, 0.5078) | **2.24E-05** | **5.61E-05** |  |
| MEM |  | -0.1534 (-0.3015, -0.0053) | **0.0425** | 0.0708 |  | -0.3704 (-0.6166, -0.1242) | **0.0035** | **0.0126** |  | 0.2139 (-0.0076, 0.4355) | 0.0583 | 0.0728 |  | -0.1361 (-0.3602, 0.0879) | 0.2315 | 0.2315 |  | -0.3535 (-0.5984, -0.1086) | **0.005** | **0.0126** |  |
| LAN |  | -0.0600 (-0.2579, 0.1380) | 0.5499 | 0.5499 |  | -0.1492 (-0.4931, 0.1947) | 0.3922 | 0.5499 |  | 0.1013 (-0.2057, 0.4083) | 0.5150 | 0.5499 |  | 0.1920 (-0.1160, 0.5000) | 0.2197 | 0.5499 |  | -0.1752 (-0.5159, 0.1655) | 0.3109 | 0.5499 |  |
| EF |  | -0.1256 (-0.4434, 0.1922) | 0.4315 | 0.909 |  | -0.1980 (-0.7617, 0.3657) | 0.4841 | 0.909 |  | -0.1512 (-0.6821, 0.3797) | 0.5702 | 0.909 |  | 0.0747 (-0.4381, 0.5876) | 0.7712 | 0.909 |  | 0.0372 (-0.6125, 0.6869) | 0.9090 | 0.9090 |  |
| Hippocampus |  | -0.0868 (-0.3678, 0.1941) | 0.5417 | 0.5417 |  | -0.4171 (-0.9021, 0.0679) | 0.0912 | 0.2279 |  | 0.2442 (-0.1654, 0.6539) | 0.2400 | 0.3656 |  | -0.2230 (-0.6408, 0.1947) | 0.2925 | 0.3656 |  | -0.6211 (-1.0842, -0.1580) | **0.0090** | **0.0450** |  |
| Entorhinal |  | -0.0786 (-0.2933, 0.1361) | 0.4698 | 0.4698 |  | -0.2014 (-0.5751, 0.1723) | 0.2879 | 0.4376 |  | 0.3815 (0.0743, 0.6886) | **0.0154** | 0.0546 |  | -0.3682 (-0.6819, -0.0545) | **0.0218** | 0.0546 |  | -0.1722 (-0.5356, 0.1913) | 0.3501 | 0.4376 |  |
| Fusiform |  | -0.1549 (-0.5777, 0.2678) | 0.4693 | 0.5867 |  | -0.4958 (-1.2295, 0.2378) | 0.1833 | 0.4583 |  | 0.6081 (-0.0022, 1.2183) | 0.0508 | 0.254 |  | -0.1573 (-0.7886, 0.4740) | 0.6225 | 0.6225 |  | -0.3280 (-1.0437, 0.3878) | 0.3659 | 0.5867 |  |
| Mid temporal |  | -0.1610 (-0.9471, 0.6250) | 0.6856 | 0.8409 |  | -0.7311 (-2.0971, 0.6350) | 0.2913 | 0.4856 |  | 0.6518 (-0.4939, 1.7975) | 0.2621 | 0.4856 |  | 0.1192 (-1.0539, 1.2923) | 0.8409 | 0.8409 |  | -0.8238 (-2.1487, 0.5011) | 0.2206 | 0.4856 |  |

P values were obtained by multiple linear regressions models adjusted for age, sex, years of education, *APOE* ɛ4 status, and cognitive status, with further adjustment for intracranial volume for brain structure outcomes. Multiple testing significance was corrected using FDR. Significant at the level of *P* < 0.05 were shown in bold. NPSs neuropsychiatric symptoms; NFL neurofilament light chain; CI Confidence Interval; FDR false discovery rate; MMSE Mini-Mental State Examination; MoCA Montreal Cognitive Assessment; CDR Clinical Dementia Rating; MEM Memory function; LAN Language; EF Executive function; *APOE* ε4 apolipoprotein ε4.

**Table S7. Cross-sectional associations of NPSs with plasma biomarkers, cognition and brain MRI in ADNI with model 3.**

|  |  | NPSs | | |  | Hyperactivity | | |  | Psychosis | | |  | Affective | | |  | Apathy | | |  |
| --- | --- | --- | --- | --- | --- | --- | --- | --- | --- | --- | --- | --- | --- | --- | --- | --- | --- | --- | --- | --- | --- |
|  |  | β (95%CI) | P | P_FDR |  | β (95%CI) | P | P_FDR |  | β (95%CI) | P | P_FDR |  | β (95%CI) | P | P_FDR |  | β (95%CI) | P | P_FDR |  |
| NFL |  | 0.0432 (0.0088, 0.0776) | **0.0141** | **0.0293** |  | 0.0330 (0.0045, 0.0615) | **0.0235** | **0.0293** |  | 0.0382 (-0.0082, 0.0847) | 0.1066 | 0.1066 |  | 0.0603 (0.0224, 0.0982) | **0.0019** | **0.0094** |  | 0.0492 (0.0068, 0.0917) | **0.0230** | **0.0293** |  |
| MMSE |  | -1.1692 (-1.3587, -0.9797) | **2.95E-32** | **1.47E-31** |  | -0.7549 (-0.9326, -0.5773) | **1.71E-16** | **2.85E-16** |  | -0.4888 (-0.7088, -0.2687) | **1.41E-05** | **1.41E-05** |  | -0.7591 (-0.9709, -0.5474) | **3.06E-12** | **3.83E-12** |  | -1.1536 (-1.3671, -0.9401) | **2.29E-25** | **5.74E-25** |  |
| ADAS13 |  | 4.4373 (3.7504, 5.1242) | **4.94E-35** | **2.47E-34** |  | 3.0597 (2.4170, 3.7024) | **3.40E-20** | **5.67E-20** |  | 2.0438 (1.2454, 2.8423) | **5.75E-07** | **5.75E-07** |  | 3.4621 (2.6977, 4.2264) | **1.79E-18** | **2.24E-18** |  | 4.3338 (3.5575, 5.1102) | **6.71E-27** | **1.68E-26** |  |
| CDRSB |  | 1.1078 (0.9867, 1.2289) | **2.00E-65** | **9.99E-65** |  | 0.8704 (0.7565, 0.9844) | **1.99E-47** | **3.31E-47** |  | 0.4415 (0.2943, 0.5886) | **4.88E-09** | **4.88E-09** |  | 0.8623 (0.7244, 1.0002) | **4.98E-33** | **6.23E-33** |  | 1.1335 (0.9962, 1.2708) | **1.62E-54** | **4.04E-54** |  |
| MEM |  | -0.2837 (-0.3367, -0.2308) | **5.19E-25** | **2.59E-24** |  | -0.1813 (-0.2307, -0.1320) | **9.01E-13** | **1.50E-12** |  | -0.1316 (-0.1924, -0.0708) | **2.32E-05** | **2.32E-05** |  | -0.2025 (-0.2611, -0.1440) | **1.68E-11** | **2.10E-11** |  | -0.2622 (-0.3219, -0.2025) | **1.71E-17** | **4.28E-17** |  |
| LAN |  | -0.2546 (-0.3040, -0.2051) | **2.93E-23** | **1.47E-22** |  | -0.1820 (-0.2278, -0.1361) | **1.30E-14** | **2.17E-14** |  | -0.1213 (-0.1779, -0.0646) | **2.84E-05** | **2.84E-05** |  | -0.1819 (-0.2366, -0.1273) | **8.73E-11** | **1.09E-10** |  | -0.2528 (-0.3083, -0.1973) | **1.17E-18** | **2.94E-18** |  |
| EF |  | -0.3554 (-0.4072, -0.3036) | **4.56E-39** | **2.28E-38** |  | -0.2563 (-0.3048, -0.2078) | **2.25E-24** | **3.75E-24** |  | -0.1468 (-0.2075, -0.0861) | **2.31E-06** | **2.31E-06** |  | -0.2820 (-0.3398, -0.2243) | **3.60E-21** | **4.50E-21** |  | -0.3223 (-0.3812, -0.2634) | **6.26E-26** | **1.57E-25** |  |
| Hippocampus |  | -0.0250 (-0.0309, -0.0191) | **1.74E-16** | **8.70E-16** |  | -0.0213 (-0.0268, -0.0158) | **5.22E-14** | **1.30E-13** |  | -0.0085 (-0.0152, -0.0019) | **0.0119** | **0.0119** |  | -0.0156 (-0.0222, -0.0090) | **3.72E-06** | **4.64E-06** |  | -0.0211 (-0.0276, -0.0146) | **3.12E-10** | **5.20E-10** |  |
| Entorhinal |  | -0.0310 (-0.0396, -0.0225) | **2.04E-12** | **1.02E-11** |  | -0.0219 (-0.0298, -0.0139) | **9.28E-08** | **1.55E-07** |  | -0.0085 (-0.0183, 0.0013) | 0.0903 | 0.0903 |  | -0.0242 (-0.0337, -0.0148) | **5.43E-07** | **6.79E-07** |  | -0.0277 (-0.0373, -0.0181) | **1.77E-08** | **4.43E-08** |  |
| Fusiform |  | -0.0148 (-0.0200, -0.0096) | **3.46E-08** | **1.73E-07** |  | -0.0089 (-0.0138, -0.0041) | **0.0003** | **0.0005** |  | -0.0025 (-0.0084, 0.0035) | 0.4186 | 0.4186 |  | -0.0084 (-0.0141, -0.0026) | **0.0045** | **0.0057** |  | -0.0129 (-0.0187, -0.0071) | **1.43E-05** | **3.57E-05** |  |
| Mid temporal |  | -0.0162 (-0.0215, -0.0108) | **3.37E-09** | **1.68E-08** |  | -0.0138 (-0.0187, -0.0089) | **4.41E-08** | **1.10E-07** |  | -0.0006 (-0.0067, 0.0055) | 0.8421 | 0.8421 |  | -0.0130 (-0.0188, -0.0071) | **1.48E-05** | **1.85E-05** |  | -0.0151 (-0.0210, -0.0092) | **6.72E-07** | **1.12E-06** |  |

P values were obtained by multiple linear regressions models adjusted for age, sex, years of education, *APOE* ɛ4 status, and biological status, with further adjustment for intracranial volume for brain structure outcomes. Multiple testing significance was corrected using FDR. Significant at the level of *P* < 0.05 were shown in bold. NPSs neuropsychiatric symptoms; NFL neurofilament light chain; ADNI Alzheimer’s Disease Neuroimaging Initiative; CI Confidence Interval; FDR false discovery rate; MMSE Mini-Mental State Examination; ADAS Alzheimer’s disease Assessment Scale; CDRSB Clinical Dementia Rating Sum of Boxes; MEM Memory function; LAN Language; EF Executive function; *APOE* ε4 apolipoprotein ε4.

**Table S8. Cross-lagged panel model results examining temporal relationships from NPSs, plasma NFL, brain atrophy, and cognitive decline.**

| Predictor |  | Outcome |  | β (95%CI) |  | SE |  | t |  | P |
| --- | --- | --- | --- | --- | --- | --- | --- | --- | --- | --- |
| NPSs_BL |  | NFL_M12 |  | 0.070 (0.010, 0.131) |  | 0.027 |  | 2.57 |  | **0.027** |
| NFL_BL |  | atrophy_M12 |  | -0.054 (-0.280, 0.173) |  | 0.089 |  | -0.60 |  | 0.573 |
| atrophy_BL |  | MMSE_M12 |  | 0.003 (-0.121, 0.127) |  | 0.051 |  | 0.06 |  | 0.953 |
| NPSs_M12 |  | NFL_M24 |  | 0.000 (-0.074, 0.074) |  | 0.032 |  | -0.01 |  | 0.995 |
| NFL_M12 |  | atrophy_M24 |  | -0.193 (-0.372, -0.016) |  | 0.072 |  | -2.68 |  | **0.037** |
| atrophy_M12 |  | MMSE_M24 |  | 0.010 (-0.096, 0.117) |  | 0.042 |  | 0.24 |  | 0.818 |

Multiple imputation with 5 imputed datasets was performed to handle missing data. Pooled estimates, standard errors, and P values were calculated according to Rubin’s rules. All variables were standardized prior to analysis. Significant at the level of P < 0.05 were shown in bold. NPSs neuropsychiatric symptoms; NFL plasma neurofilament light chain; MMSE Mini-Mental State Examination; CI Confidence Interval; SE standard error; BL baseline; M12 12-month follow-up; M24 24-month follow-up.

**Table S9. Sequential mediation models linking NPSs to cognition in the overall ADNI cohort.**

| Models | Total Effect | | |  | Direct Effect | | |  | Total Indirect Effect | | |  | Pathway 1 | | |  | Pathway 2 | | |  | Pathway 3 | | |  |
| --- | --- | --- | --- | --- | --- | --- | --- | --- | --- | --- | --- | --- | --- | --- | --- | --- | --- | --- | --- | --- | --- | --- | --- | --- |
|  | β (95%CI) | P | P_FDR |  | β (95%CI) | P | P_FDR |  | β (95%CI) | P | P_FDR |  | β (95%CI) | P | P_FDR |  | β (95%CI) | P | P_FDR |  | β (95%CI) | P | P_FDR |  |
| NPSs-NFL-ROI-MMSE | -1.5167 (-2.3143, -1.2242) | **9.96E-09** | **3.32E-08** |  | -0.6799(-1.1386, -0.2311) | **0.0018** | **0.0046** |  | -0.8377 (-1.1956, -0.4962) | **<0.001** | **<0.001** |  | -0.1321 (-0.2633, -0.0324) | **0.0060** | **0.0106** |  | -0.6719 (-1.0156, -0.3977) | **<0.001** | **<0.001** |  | -0.0337 (-0.1248, 0.0161) | 0.2660 | 0.4433 |  |
| NPSs-NFL-ROI-ADAS13 | 6.5165 (5.3947 ,9.2428) | **8.68E-12** | **8.68E-11** |  | 3.1905 (1.5998, 4.4624) | **8.01E-06** | **8.01E-05** |  | 3.3294 (2.1128, 4.9341) | **<0.001** | **<0.001** |  | 0.4936 (0.1143, 0.9945) | **0.0140** | **0.0233** |  | 2.5443 (1.4350 ,3.7541) | **<0.001** | **<0.001** |  | 0.2915 (0.0732, 0.7357) | **0.0140** | 0.0700 |  |
| NPSs-NFL-ROI-CDRSB | 1.2466 (1.0140 ,1.7403) | **3.81E-12** | **5.72E-11** |  | 0.6822 (0.3520, 1.0132) | **3.28E-06** | **4.92E-05** |  | 0.5652 (0.3280 ,0.8219 | **<0.001** | **<0.001** |  | 0.0857 (0.0204, 0.1722) | **0.0060** | **0.0106** |  | 0.4357 (0.2510 ,0.6731) | **<0.001** | **<0.001** |  | 0.0438 (0.0065, 0.1088) | **0.0340** | 0.1020 |  |
| NPSs-NFL-ROI-MEM | -0.4997 (-0.7134, -0.4405) | **1.70E-12** | **5.11E-11** |  | -0.2651 (-0.3701, - 0.1608) | **1.84E-06** | **4.92E-05** |  | -0.2349 (-0.3300, -0.1333) | **<0.001** | **<0.001** |  | -0.0346 (-0.0699, -0.0086) | **0.0060** | **0.0106** |  | -0.1759 (-0.2611, -0.1023) | **<0.001** | **<0.001** |  | -0.0244 (-0.0595, -0.0061) | **0.0060** | **0.0360** |  |
| NPSs-NFL-ROI-LAN | -0.3111 (-0.4758, -0.2038) | **4.59E-06** | **7.65E-06** |  | -0.1313 (-0.2442, -0.0090) | **0.0309** | **0.0488** |  | -0.1801 (-0.2635, -0.1099) | **<0.001** | **<0.001** |  | -0.0267 (-0.0566, -0.0069) | **0.0060** | **0.0106** |  | -0.1356 (-0.2094, -0.0814) | **<0.001** | **<0.001** |  | -0.0178 (-0.0522, -0.0026) | **0.0240** | 0.0933 |  |
| NPSs-NFL-ROI-EF | -0.3561 (-0.5610, -0.2536) | **1.31E-06** | **2.62E-06** |  | -0.1652 (-0.2918, -0.0243) | **0.0130** | **0.0244** |  | -0.1910 (-0.2782, -0.1140) | **<0.001** | **<0.001** |  | -0.0306 (-0.0657, -0.0082) | **0.0060** | **0.0106** |  | -0.1555 (-0.2417, -0.0923) | **<0.001** | **<0.001** |  | -0.0050 (-0.0291 ,0.0106) | 0.5700 | 0.7108 |  |
| HYP-NFL-ROI-MMSE | -0.9956 (-1.6392, -0.7493) | **8.86E-06** | **1.40E-05** |  | -0.3716 (-0.7100, -0.0171) | **0.0407** | 0.0611 |  | -0.6250 (-0.8723, -0.3698) | **<0.001** | **<0.001** |  | -0.0900 (-0.2080, -0.0042) | **0.0460** | 0.0600 |  | -0.5123 (-0.7498, -0.3132) | **<0.001** | **<0.001** |  | -0.0227 (-0.0890 ,0.0089) | 0.2540 | 0.4433 |  |
| HYP-NFL-ROIADAS13 | 4.4898 (3.6912 ,6.8403) | **2.87E-08** | **7.82E-08** |  | 2.0456 (0.8590, 3.2368) | **5.86E-04** | **0.0016** |  | 2.4482 (1.4568, 3.4913) | **<0.001** | **<0.001** |  | 0.3307 (0.0069, 0.7669) | **0.0480** | 0.0600 |  | 1.9280 (1.1220, 2.6937) | **<0.001** | **<0.001** |  | 0.1895 (0.0055 ,0.5111) | **0.0480** | 0.1108 |  |
| HYP-NFL-ROI-CDRSB | 0.9102 (0.7358 ,1.2971) | **2.07E-09** | **1.24E-08** |  | 0.4929 (0.2240, 0.7765) | **5.30E-05** | **3.18E-04** |  | 0.4182 (0.2512, 0.6081) | **<0.001** | **<0.001** |  | 0.0581 (0.0049, 0.1361) | **0.0460** | 0.0600 |  | 0.3308 (0.2019 ,0.4920) | **<0.001** | **<0.001** |  | 0.0292 (0.0010 ,0.0861) | 0.0660 | 0.1238 |  |
| HYP-NFL-ROI-MEM | -0.3481 (-0.5189, -0.3235) | **6.96E-09** | **2.95E-08** |  | -0.1744 (-0.2507, -0.0997) | **1.69E-04** | **7.22E-04** |  | -0.1741 (-0.2506, -0.1055) | **<0.001** | **<0.001** |  | -0.0236 (-0.0530, -0.0011) | **0.0460** | 0.0600 |  | -0.1344 (-0.1965, -0.0839) | **<0.001** | **<0.001** |  | -0.0161 (-0.0437, -0.0016) | 0.0460 | 0.1108 |  |
| HYP-NFL-ROI-LAN | -0.2253 (-0.3644, -0.1693) | **8.05E-05** | **1.15E-04** |  | -0.0939 (-0.1828 ,0.0028) | 0.0624 | 0.0891 |  | -0.1316 (-0.1908, -0.0814) | **<0.001** | **<0.001** |  | -0.0179 (-0.0411, -0.0011) | **0.0460** | 0.0600 |  | -0.1021 (-0.1526, -0.0659) | **<0.001** | **<0.001** |  | -0.0116 (-0.0383, -0.0005) | 0.0620 | 0.1238 |  |
| HYP-NFL-ROI-EF | -0.2225 (-0.3787, -0.1751) | **3.46E-04** | **4.52E-04** |  | -0.0790 (-0.1795 ,0.0282) | 0.1531 | 0.1827 |  | -0.1438 (-0.2108, -0.0905) | **<0.001** | **<0.001** |  | -0.0210 (-0.0477, -0.0015) | **0.0460** | 0.0600 |  | -0.1193 (-0.1767, -0.0712) | **<0.001** | **<0.001** |  | -0.0035 (-0.0234 ,0.0061) | 0.5620 | 0.7108 |  |
| PSYNFL-ROI-MMSE | -0.8175 (-1.8938, -0.2926) | **0.0269** | **0.0310** |  | -0.4064 (-1.0237 ,0.1053) | 0.1583 | 0.1827 |  | -0.4129 (-0.8846 ,0.1484) | 0.1460 | 0.1593 |  | -0.0439 (-0.2251 ,0.1201) | 0.6020 | 0.6228 |  | -0.3586 (-0.7658, 0.0884) | 0.1040 | 0.1076 |  | -0.0104 (-0.1235 ,0.0178) | 0.7480 | 0.7738 |  |
| PSY-NFL-ROI-ADAS13 | 2.7849 (0.4525 ,6.7050) | **0.0411** | **0.0457** |  | 1.2598 (-0.3475 ,3.0230) | 0.1904 | 0.2115 |  | 1.5320 (-0.7140, 3.6233) | 0.1960 | 0.1960 |  | 0.1412 (-0.4783 ,0.8714) | 0.7040 | 0.7040 |  | 1.3170 (-0.3351, 3.0160) | 0.1220 | 0.1220 |  | 0.0738 (-0.2382 ,0.4649) | 0.7040 | 0.7543 |  |
| PSY-NFL-ROI-CDRSB | 0.7695 (0.5091 ,1.5685) | **0.0023** | **0.0028** |  | 0.4894 (0.1414 ,0.9247) | 0.0119 | 0.0239 |  | 0.2815 (-0.1188, 0.6281) | 0.1520 | 0.1593 |  | 0.0293 (-0.0779 ,0.1628) | 0.6020 | 0.6228 |  | 0.2389 (-0.0578, 0.5303) | 0.1040 | 0.1076 |  | 0.0134 (-0.0298 ,0.0935) | 0.6160 | 0.7108 |  |
| PSY-NFL-ROI-MEM | -0.2028 (-0.4680, -0.0394) | **0.0431** | **0.0462** |  | -0.0871 (-0.2141 ,0.0387) | 0.2404 | 0.2575 |  | -0.1164 (-0.2603, 0.0494) | 0.1540 | 0.1593 |  | -0.0119 (-0.0589 ,0.0329) | 0.6020 | 0.6228 |  | -0.0972 (-0.2083, 0.0227) | 0.1040 | 0.1076 |  | -0.0073 (-0.0381 ,0.0203) | 0.6020 | 0.7108 |  |
| PSY-NFL-ROI-LAN | -0.1198 (-0.3296 ,0.0653) | 0.2030 | 0.2100 |  | -0.0335 (-0.1585 ,0.1065) | 0.6754 | 0.6987 |  | -0.0868 (-0.1992 ,0.0337) | 0.1540 | 0.1593 |  | -0.0089 (-0.0459 ,0.0231) | 0.6020 | 0.6228 |  | -0.0726 (-0.1634 ,0.0128) | 0.1040 | 0.1076 |  | -0.0053 (-0.0352 ,0.0116) | 0.6160 | 0.7108 |  |
| PSY-NFL-ROI-EF | -0.0856 (-0.3602 ,0.0797) | 0.4030 | 0.4030 |  | 0.0102 (-0.1612 ,0.1793) | 0.9075 | 0.9075 |  | -0.0961 (-0.2040 ,0.0346) | 0.1400 | 0.1593 |  | -0.0103 (-0.0553 ,0.0274) | 0.6020 | 0.6228 |  | -0.0843 (-0.1847, 0.0154) | 0.1040 | 0.1076 |  | -0.0016 (-0.0244 ,0.0057) | 0.8720 | 0.8720 |  |
| AFF-NFL-ROI-MMSE | -1.4155 (-2.3782, -1.1093) | **2.81E-06** | **5.19E-06** |  | -0.6814 (-1.1561, -0.1647) | **0.0051** | **0.0108** |  | -0.7333 (-1.1273, -0.3321) | **<0.001** | **<0.001** |  | -0.1841 (-0.3495, -0.0680) | **0.0020** | **0.0055** |  | -0.5111 (-0.8770, -0.1483) | **0.0040** | **0.0052** |  | -0.0381 (-0.1707 ,0.0289) | 0.3600 | 0.5400 |  |
| AFF-NFL-ROI-ADAS13 | 5.7636 (4.8097,8.7715) | **1.56E-07** | **3.60E-07** |  | 2.7653 (1.2782 ,4.2343) | **5.53E-04** | **0.0016** |  | 2.9948 (1.4817 ,4.9085) | **<0.001** | **<0.001** |  | 0.6921 (0.2636 ,1.3117) | **0.0040** | **0.0100** |  | 1.9433 (0.5684 ,3.5515) | **0.0100** | **0.0125** |  | 0.3594 (0.0985 ,0.7619) | **0.0040** | **0.0300** |  |
| AFF-NFL-ROI-CDRSB | 1.1246 (0.9156,1.7374) | **4.95E-08** | **1.24E-07** |  | 0.6105 (0.2615 ,1.0368) | **1.98E-04** | **7.43E-04** |  | 0.5135 (0.2380 ,0.7980) | **<0.001** | **<0.001** |  | 0.1221 (0.0487 ,0.2474) | **0.0020** | **0.0055** |  | 0.3390 (0.1059 ,0.5883) | **0.0040** | **0.0052** |  | 0.0524 (0.0049 ,0.1430) | **0.0440** | 0.1108 |  |
| AFF-NFL-ROI-MEM | -0.4687 (-0.7013, -0.4289) | **7.85E-09** | **2.95E-08** |  | -0.2534 (-0.3609, -0.1504) | **4.50E-05** | **3.18E-04** |  | -0.2151 (-0.3280, -0.0951) | **<0.001** | **<0.001** |  | -0.0490 (-0.0880, -0.0171) | **0.0020** | **0.0055** |  | -0.1361 (-0.2377, -0.0394) | **0.0040** | **0.0052** |  | -0.0299 (-0.0694, -0.0082) | **0.0020** | **0.0200** |  |
| AFF-NFL-ROI-LAN | -0.2844 (-0.4726, -0.1937) | **2.35E-04** | **3.20E-04** |  | -0.1211 (-0.2297, -0.0087) | 0.0735 | 0.1002 |  | -0.1631 (-0.2542, -0.0783) | **<0.001** | **<0.001** |  | -0.0372 (-0.0704, -0.0123) | **0.0020** | **0.0055** |  | -0.1034 (-0.1794, -0.0334) | **0.0040** | **0.0052** |  | -0.0225 (-0.0599, -0.0047) | **0.0280** | 0.0933 |  |
| AFF-NFL-ROI-EF | -0.2881 (-0.5298, -0.1753) | **6.12E-04** | **7.64E-04** |  | -0.1194 (-0.2641 ,0.0363) | 0.1076 | 0.1345 |  | -0.1686 (-0.2618, -0.0758) | **<0.001** | **<0.001** |  | -0.0432 (-0.0819, -0.0163) | **0.0020** | **0.0055** |  | -0.1198 (-0.2051, -0.0368) | **0.0040** | **0.0052** |  | -0.0056 (-0.0366 ,0.0190) | 0.6080 | 0.7108 |  |
| APA-NFL-ROI-MMSE | -1.6028 (-2.5856, -1.1409) | **8.43E-07** | **1.81E-06** |  | -0.6389 (-1.2908 ,0.0799) | **0.0163** | **0.0272** |  | -0.9635 (-1.5372, -0.5152) | **<0.001** | **<0.001** |  | -0.2143 (-0.4186, -0.0802) | **0.0020** | **0.0055** |  | -0.7017 (-1.1576, -0.3058) | **<0.001** | **<0.001** |  | -0.0475 (-0.1844 ,0.0366) | 0.3480 | 0.5400 |  |
| APA-NFL-ROI-ADAS13 | 6.7054 (5.3916,10.1412) | **1.19E-08** | **3.58E-08** |  | 2.6574 (1.0977, 4.2196) | **0.0024** | **0.0056** |  | 4.0466 (2.2643 ,6.4067) | **<0.001** | **<0.001** |  | 0.8239 (0.3075, 1.6127) | **0.0020** | **0.0055** |  | 2.7716 (1.1751, 4.4269) | **<0.001** | **<0.001** |  | 0.4512 (0.1214 ,1.0533) | **0.0020** | **0.0200** |  |
| APA-NFL-ROI-CDRSB | 1.2918 (0.9926,1.9467) | **5.71E-09** | **2.85E-08** |  | 0.6243 (0.1007, 1.1754) | **5.05E-04** | **0.0016** |  | 0.6671 (0.3606, 1.0830) | **<0.001** | **<0.001** |  | 0.1411 (0.0554, 0.2791) | **0.0020** | **0.0055** |  | 0.4622 (0.1870, 0.7683) | **<0.001** | **<0.001** |  | 0.0638 (0.0068 ,0.1710) | 0.0560 | 0.1200 |  |
| APA-NFL-ROI-MEM | -0.5351(-0.7588, -0.4809) | **8.90E-10** | **6.68E-09** |  | -0.2563 (-0.3832, -0.1362) | **1.64E-04** | **7.22E-04** |  | -0.2787 (-0.4323, -0.1477) | **<0.001** | **<0.001** |  | -0.0567 (-0.1099, -0.0220) | **0.0020** | **0.0055** |  | -0.1857 (-0.2993, -0.0813) | **<0.001** | **<0.001** |  | -0.0363 (-0.0896, -0.0111) | **0.0020** | **0.0200** |  |
| APA-NFL-ROI-LAN | -0.3874 (-0.5929, -0.2837) | **2.94E-06** | **5.19E-06** |  | -0.1797 (-0.3100, -0.0539) | **0.0149** | **0.0262** |  | -0.2076 (-0.3344, -0.1194) | **<0.001** | **<0.001** |  | -0.0424 (-0.0836, -0.0160) | **0.0020** | **0.0055** |  | -0.1388 (-0.2372, -0.0639) | **<0.001** | **<0.001** |  | -0.0264 (-0.0744, -0.0031) | **0.0260** | 0.0933 |  |
| APA-NFL-ROI-EF | -0.3584 (-0.6131, -0.2285) | **7.39E-05** | **1.11E-04** |  | -0.1384 (-0.3362 ,0.0552) | 0.0878 | 0.1145 |  | -0.2200 (-0.3615, -0.1225) | **<0.001** | **<0.001** |  | -0.0499 (-0.1029, -0.0198) | **0.0020** | **0.0055** |  | -0.1634 (-0.2812, -0.0699) | **<0.001** | **<0.001** |  | -0.0067 (-0.0420, 0.0205) | 0.6400 | 0.7111 |  |

Mediation analysis based on structural equation modeling identified three potential mediation pathways: (1) *NPSs → plasma NFL→ AD-signature ROI → cognition*, (2) *NPSs→ AD-signature ROI → cognition*, and (3) *NPSs → plasma NFL → cognition*. **All analyses were adjusted for age, sex, education, and APOE ε4 status, with intracranial volume additionally included as a covariate in models involving brain structure measures.** Multiple testing significance was corrected using FDR. Significant at the level of *P* < 0.05 were shown in bold. NPSs neuropsychiatric symptoms; HYP hyperactivity; PSY psychosis; AFF affective; APA apathy; MMSE Mini-Mental State Examination; ADAS Alzheimer’s disease Assessment Scale; CDRSB Clinical Dementia Rating Sum of Boxes; MEM Memory function; LAN Language; EF Executive function; NFL neurofilament light chain; ROI region of interest; FDR false discovery rate; CI Confidence Interval.

**Table S10. Sequential mediation models linking NPSs to cognition in the ADNI female subsample.**

| Models |  | Total Effect | | |  | Direct Effect | | |  | Total Indirect Effect | | |  | Pathway 1 | | |  | Pathway 2 | | |  | Pathway 3 | | |
| --- | --- | --- | --- | --- | --- | --- | --- | --- | --- | --- | --- | --- | --- | --- | --- | --- | --- | --- | --- | --- | --- | --- | --- | --- |
|  |  | β (95%CI) | P | P_FDR |  | β (95%CI) | P | P_FDR |  | β (95%CI) | P | P_FDR |  | β (95%CI) | P | P_FDR |  | β (95%CI) | P | P_FDR |  | β (95%CI) | P | P_FDR |
| NPSs-NFL-ROI-MMSE |  | -2.5189 (-3.6196, -1.6960) | **4.18E-07** | **7.08E-06** |  | -1.2446 (-2.1755, -0.3702) | **0.0007** | **0.0096** |  | -1.2750 (-2.1333, -0.6155) | **<0.001** | **<0.001** |  | -0.3345 (-0.7040, -0.0715) | **0.0160** | **0.0200** |  | -0.8684 (-1.7594, -0.3097) | **0.0020** | **0.0120** |  | -0.0721 (-0.3781, 0.0756 | 0.4640 | 0.6574 |
| NPSs-NFL-ROI-ADAS13 |  | 9.2796 (6.3565, 13.6740) | **3.27E-06** | **3.27E-05** |  | 3.8261 (0.7258, 6.7386) | **0.0058** | **0.0250** |  | 5.4567 (2.4065, 9.1376) | **<0.001** | **<0.001** |  | 1.4135 (0.2633, 2.9346) | **0.0140** | **0.0200** |  | 3.6423 (1.0786, 7.1491) | **0.0040** | **0.0200** |  | 0.4008 (-0.1640, 1.4622) | 0.2660 | 0.6574 |
| NPSs-NFL-ROI-CDRSB |  | 1.3845 (0.7622, 2.2213) | **0.0002** | **3.84E-04** |  | 0.5318 (-0.2711, 1.3128) | 0.0792 | 0.1244 |  | 0.8532 (0.4207, 1.4812) | **<0.001** | **<0.001** |  | 0.2167 (0.0510, 0.4894) | **0.0160** | **0.0200** |  | 0.5625 (0.1930, 1.1686) | **0.0020** | **0.0120** |  | 0.0740 (-0.0445, 0.4025) | 0.3300 | 0.6574 |
| NPSs-NFL-RO-MEM |  | -0.7251 (-1.0570, -0.5171) | **0.0001** | **7.08E-06** |  | -0.3514 (-0.6036, -0.1306) | **0.0009** | **0.0096** |  | -0.3739 (-0.6227, -0.1800) | **<0.001** | **<0.001** |  | -0.0912 (-0.1834, -0.0219) | **0.0160** | **0.0200** |  | -0.2368 (-0.4760, -0.0799) | **0.0020** | **0.0120** |  | -0.0459 (-0.1377, -0.0038) | 0.0600 | 0.3450 |
| NPSs-NFL-ROI-LAN |  | -0.5051 (-0.7692, -0.2746) | **0.0001** | **1.76E-04** |  | -0.2365 (-0.4021, 0.0382) | **0.0275** | 0.0669 |  | -0.2688 (-0.4676, -0.1403) | **<0.001** | **<0.001** |  | -0.0642 (-0.1431, -0.0193) | **0.0160** | **0.0200** |  | -0.1666 (-0.3519, -0.0613) | **0.0020** | **0.0120** |  | -0.0379 (-0.1229, -0.0027) | 0.0740 | 0.3450 |
| NPSs-NFL-ROI-EF |  | -0.5359 (-0.8671, -0.2415) | **0.0001** | **2.88E-04** |  | -0.2682 (-0.5506, 0.0469) | **0.0278** | 0.0669 |  | -0.2678 (-0.4965, -0.1226) | **<0.001** | **<0.001** |  | -0.0812 (-0.1837, -0.0225) | **0.0160** | **0.0200** |  | -0.2107 (-0.4356, -0.0765) | **0.0020** | **0.0120** |  | 0.0241 (-0.0303, 0.1192) | 0.4200 | 0.6574 |
| HYP-NFL-ROI-MMSE |  | -1.6077 (-2.6656, -1.0010) | **0.0004** | **7.85E-04** |  | -0.5947 (-1.4113, 0.0071) | 0.0666 | 0.1175 |  | -1.0125 (-1.5597, -0.4876) | **<0.001** | **<0.001** |  | -0.3159 (-0.6725, -0.0892) | **0.0140** | **0.0200** |  | -0.6305 (-1.1836, -0.1993) | **0.0120** | **0.0300** |  | -0.0661 (-0.3164, 0.0546) | 0.4060 | 0.6574 |
| HYP-NFL-ROIADAS13 |  | 7.3154 (5.6091, 10.5921) | **3.58E-05** | **1.36E-04** |  | 3.1856 (0.5821, 5.5085) | **0.0079** | **0.0274** |  | 4.1283 (2.0479, 6.8771) | **<0.001** | **<0.001** |  | 1.2636 (0.2814, 2.6657) | **0.0120** | **0.0200** |  | 2.5397 (0.7045, 4.9533) | **0.0100** | **0.0300** |  | 0.3250 (-0.1143, 1.3516) | 0.2580 | 0.6574 |
| HYP-NFL-ROI-CDRSB |  | 1.1147 (0.7080, 1.7498) | **0.0006** | **0.0012** |  | 0.4677 (-0.1566, 1.0806) | 0.0744 | 0.1241 |  | 0.6467 (0.3166, 1.0642) | **<0.001** | **<0.001** |  | 0.1955 (0.0611, 0.4311) | **0.0140** | **0.0200** |  | 0.3903 (0.1078, 0.7846) | **0.0120** | **0.0300** |  | 0.0608 (-0.0378, 0.3036) | 0.3480 | 0.6574 |
| HYP-NFL-ROI-MEM |  | -0.5361 (-0.7677, -0.4354) | **3.27E-05** | **1.36E-04** |  | -0.2436 (-0.4230, -0.0828) | **0.0082** | 0.0274 |  | -0.2924 (-0.4550, -0.1445) | **<0.001** | **<0.001** |  | -0.0846 (-0.1788, -0.0250) | **0.0140** | **0.0200** |  | -0.1688 (-0.3197, -0.0483) | **0.0120** | **0.0300** |  | -0.0391 (-0.1187, -0.0018) | 0.0560 | 0.3450 |
| HYP-NFL-ROI-LAN |  | -0.3150 (-0.5438, -0.1545) | **0.0050** | **0.0069** |  | -0.0986 (-0.2414, 0.1199) | 0.2925 | 0.3510 |  | -0.2163 (-0.3704, -0.1179) | **<0.001** | **<0.001** |  | -0.0609 (-0.1391, -0.0204) | **0.0140** | **0.0200** |  | -0.1216 (-0.2260, -0.0445) | **0.0120** | **0.0300** |  | -0.0338 (-0.1065, -0.0023) | 0.0700 | 0.3450 |
| HYP-NFL-ROI-EF |  | -0.2821 (-0.5620, -0.1325) | **0.0234** | **0.0293** |  | -0.0684 (-0.2817, 0.1984) | 0.5215 | 0.5587 |  | -0.2137 (-0.3664, -0.0987) | **<0.001** | **<0.001** |  | -0.0775 (-0.1736, -0.0217) | **0.0140** | **0.0200** |  | -0.1547 (-0.3066, -0.0525) | **0.0120** | **0.0300** |  | 0.0186 (-0.0414, 0.0890) | 0.4920 | 0.6574 |
| PSYNFL-ROI-MMSE |  | -1.0740 (-3.3754, 0.7274) | 0.1719 | 0.2063 |  | -1.1860 (-2.7504, -0.3226) | **0.0235** | 0.0669 |  | 0.1037 (-0.9255, 1.2734) | 0.9020 | 0.9260 |  | -0.0608 (-0.6823, 0.3554) | 0.8120 | 0.8120 |  | 0.1768 (-0.5837, 1.3822) | 0.7680 | 0.7680 |  | -0.0123 (-0.3660, 0.0710) | 0.9100 | 0.9100 |
| PSY-NFL-ROI-ADAS13 |  | 1.5405 (-4.5096, 8.6080) | 0.6318 | 0.6769 |  | 2.0033 (-1.4232, 5.4076) | 0.3233 | 0.3677 |  | -0.4271 (-5.2575, 3.7436) | 0.9180 | 0.9260 |  | 0.3026 (-1.7321, 2.6736) | 0.7520 | 0.8120 |  | -0.8085 (-5.4788, 2.4319) | 0.7400 | 0.7680 |  | 0.0788 (-0.3843, 1.2110) | 0.8240 | 0.9100 |
| PSY-NFL-ROI-CDRSB |  | 0.6108 (-0.3756, 1.8670) | 0.2815 | 0.3248 |  | 0.6776 (-0.0183, 1.4072) | 0.1117 | 0.1595 |  | -0.0612 (-0.8087, 0.5871) | 0.9020 | 0.9260 |  | 0.0381 (-0.2294, 0.4389) | 0.8120 | 0.8120 |  | -0.1106 (-0.9343, 0.3661) | 0.7680 | 0.7680 |  | 0.0113 (-0.0701, 0.2558) | 0.8820 | 0.9100 |
| PSY-NFL-ROI-MEM |  | -0.1204 (-0.6624, 0.3577) | 0.5975 | 0.6639 |  | -0.1474 (-0.3595, 0.1223) | 0.3309 | 0.3677 |  | 0.0243 (-0.2635, 0.3743) | 0.9200 | 0.9260 |  | -0.0166 (-0.1749, 0.0977) | 0.8120 | 0.8120 |  | 0.0482 (-0.1566, 0.3583) | 0.7680 | 0.7680 |  | -0.0073 (-0.1154, 0.0458) | 0.8240 | 0.9100 |
| PSY-NFL-ROI-LAN |  | 0.0715 (-0.3935, 0.6380) | 0.7129 | 0.7375 |  | 0.0535 (-0.1586, 0.4242) | 0.7253 | 0.7503 |  | 0.0160 (-0.1808, 0.2608) | 0.9260 | 0.9260 |  | -0.0115 (-0.1226, 0.0706) | 0.8120 | 0.8120 |  | 0.0336 (-0.1035, 0.2547) | 0.7680 | 0.7680 |  | -0.0060 (-0.0806, 0.0341) | 0.8140 | 0.9100 |
| PSY-NFL-ROI-EF |  | -0.0026 (-0.5548, 0.4888) | 0.9902 | 0.9902 |  | -0.0345 (-0.4737, 0.2132) | 0.8422 | 0.8422 |  | 0.0305 (-0.1738, 0.3153) | 0.8460 | 0.9260 |  | -0.0145 (-0.1458, 0.0914) | 0.8120 | 0.8120 |  | 0.0421 (-0.1341, 0.3331) | 0.7680 | 0.7680 |  | 0.0029 (-0.0251, 0.0938) | 0.8980 | 0.9100 |
| AFF-NFL-ROI-MMSE |  | -2.4012 (-3.7247, -1.6653) | **8.79E-06** | **5.27E-05** |  | -1.2180 (-2.2160, -0.3646) | **0.0017** | **0.0109** |  | -1.1756 (-2.2354, -0.4588) | **0.0040** | **0.0071** |  | -0.3784 (-0.7804, -0.1466) | **0.0040** | **0.0100** |  | -0.7292 (-1.7379, 0.0079) | 0.0840 | 0.1400 |  | -0.0681 (-0.3431, 0.1039) | 0.5040 | 0.6574 |
| AFF-NFL-ROI-ADAS13 |  | 8.2749 (5.5428, 13.8050) | **0.0001** | **3.31E-04** |  | 3.2033 (0.1214, 6.3656) | **0.0290** | 0.0669 |  | 5.0380 (1.6778, 8.6333) | **0.0060** | **0.0095** |  | 1.5746 (0.4809, 3.1590) | **0.0020** | **0.0086** |  | 3.0546 (-0.1140, 6.7653) | 0.0660 | 0.1400 |  | 0.4088 (-0.2194, 1.4606) | 0.2660 | 0.6574 |
| AFF-NFL-ROI-CDRSB |  | 1.2634 (0.7576, 2.1166) | **0.0015** | **0.0023** |  | 0.4689 (-0.2317, 1.4085) | 0.1427 | 0.1945 |  | 0.7891 (0.2976, 1.4409) | **0.0060** | **0.0095** |  | 0.2435 (0.0930, 0.5141) | **0.0040** | **0.0100** |  | 0.4693 (-0.0037, 1.1301) | 0.0840 | 0.1400 |  | 0.0762 (-0.0562, 0.3567) | 0.3180 | 0.6574 |
| AFF-NFL-ROI-MEM |  | -0.6985 (-1.0934, -0.5590) | **7.58E-06** | **5.27E-05** |  | -0.3476 (-0.5788, -0.1195) | **0.0018** | **0.0109** |  | -0.3484 (-0.6222, -0.1323) | **0.0040** | **0.0071** |  | -0.1032 (-0.1940, -0.0346) | **0.0040** | **0.0100** |  | -0.1989 (-0.4822, 0.0007) | 0.0840 | 0.1400 |  | -0.0463 (-0.1297, 0.0006) | 0.0620 | 0.3450 |
| AFF-NFL-ROI-LAN |  | -0.4530 (-0.8242, -0.3501) | **0.0008** | **0.0015** |  | -0.1968 (-0.3734, 0.0044) | 0.0829 | 0.1244 |  | -0.2543 (-0.4859, -0.1013) | **0.0040** | **0.0071** |  | -0.0734 (-0.1559, -0.0277) | **0.0040** | **0.0100** |  | -0.1414 (-0.3288, -0.0084) | 0.0840 | 0.1400 |  | -0.0395 (-0.1242, -0.0005) | 0.0760 | 0.3450 |
| AFF-NFL-ROI-EF |  | -0.4042 (-0.7977, -0.1858) | **0.0073** | **0.0095** |  | -0.1528 (-0.4120, 0.1405) | 0.2380 | 0.2975 |  | -0.2503 (0.5155, -0.0787) | **0.0080** | **0.0114** |  | -0.0940 (-0.2083, -0.0349) | **0.0040** | **0.0100** |  | -0.1811 (-0.4288, -0.0122) | 0.0840 | 0.1400 |  | 0.0248 (-0.0504, 0.1186) | 0.4420 | 0.6574 |
| APA-NFL-ROI-MMSE |  | -2.4693 (-4.1682, -1.3713) | **3.62E-05** | **1.36E-04** |  | -1.4026 (-2.6231, -0.0242) | **0.0010** | **0.0096** |  | -1.0625 (-2.3903, -0.1476) | **0.0120** | **0.0157** |  | -0.5333 (-1.1723, -0.1763) | **0.0020** | **0.0086** |  | -0.4754 (-1.5680, 0.5178) | 0.3360 | 0.4200 |  | -0.0538 (-0.3937, 0.2201) | 0.6960 | 0.8700 |
| APA-NFL-ROI-ADAS13 |  | 8.0121 (4.9166, 15.1235) | **0.0008** | **0.0015** |  | 3.3559 (-0.3310, 7.2440) | **0.0368** | 0.0789 |  | 4.6372 (0.6861, 9.7108) | **0.0100** | **0.0136** |  | 2.2002 (0.7432, 4.5180) | **0.0020** | **0.0086** |  | 1.9728 (-2.0433, 6.8831) | 0.2960 | 0.4200 |  | 0.4643 (-0.3868, 1.8853) | 0.3880 | 0.6574 |
| APA-NFL-ROI-CDRSB |  | 1.4131 (0.5471, 2.2927) | **0.0012** | **0.0020** |  | 0.6943 (-0.6454, 1.8113) | **0.0467** | 0.0933 |  | 0.7158 (0.0933, 1.5617) | **0.0080** | **0.0114** |  | 0.3368 (0.1181, 0.7802) | **0.0020** | **0.0086** |  | 0.3002 (-0.3424, 0.9904) | 0.3360 | 0.4200 |  | 0.0788 (-0.1205, 0.3750) | 0.4600 | 0.6574 |
| APA-NFL-ROI-MEM |  | -0.6957 (-1.1231, -0.5305) | **0.0001** | **1.76E-04** |  | -0.3647 (-0.6537, -0.0894) | **0.0029** | **0.0144** |  | -0.3296 (-0.7103, -0.0719) | **0.0020** | **0.0046** |  | -0.1462 (-0.3249, -0.0547) | **0.0020** | **0.0086** |  | -0.1304 (-0.4380, 0.1624) | 0.3360 | 0.4200 |  | -0.0530 (-0.1452, 0.0095) | 0.0900 | 0.3450 |
| APA-NFL-ROI-LAN |  | -0.4234 (-0.8515, -0.2276) | **0.0048** | **0.0069** |  | -0.1769 (-0.3898, -0.0234) | 0.1560 | 0.2034 |  | -0.2455 (-0.5366, -0.0625) | **0.0040** | **0.0071** |  | -0.1037 (-0.2295, -0.0381) | **0.0020** | **0.0086** |  | -0.0925 (-0.3092, 0.0927) | 0.3360 | 0.4200 |  | -0.0493 (-0.1538, -0.0009) | 0.0920 | 0.3450 |
| APA-NFL-ROI-EF |  | -0.4707 (-0.9577, -0.1874) | **0.0044** | **0.0067** |  | -0.2685 (-0.6123, 0.2473) | 0.0571 | 0.1071 |  | -0.2016 (-0.4996, 0.0278) | 0.0540 | 0.0675 |  | -0.1291 (-0.2979, -0.0484) | **0.0020** | **0.0086** |  | -0.1151 (-0.3835, 0.1196) | 0.3360 | 0.4200 |  | 0.0425 (-0.0535, 0.1726) | 0.3200 | 0.6574 |

Mediation analysis based on structural equation modeling identified three potential mediation pathways: (1) *NPSs → plasma NFL→ AD-signature ROI → cognition*, (2) *NPSs→ AD-signature ROI → cognition*, and (3) *NPSs → plasma NFL → cognition*. **All analyses were adjusted for age, sex, education, and APOE ε4 status, with intracranial volume additionally included as a covariate in models involving brain structure measures.** Multiple testing significance was corrected using FDR. Significant at the level of *P* < 0.05 were shown in bold. NPSs neuropsychiatric symptoms; HYP hyperactivity; PSY psychosis; AFF affective; APA apathy; MMSE Mini-Mental State Examination; ADAS Alzheimer’s disease Assessment Scale; CDRSB Clinical Dementia Rating Sum of Boxes; MEM Memory function; LAN Language; EF Executive function; NFL neurofilament light chain; ROI region of interest; FDR false discovery rate; CI Confidence Interval.

**Table S11. Sequential mediation models linking NPSs to cognition in the ADNI male subsample.**

| Models |  | Total Effect | | |  | Direct Effect | | |  | Total Indirect Effect | | |  | Pathway 1 | | |  | Pathway 2 | | |  | Pathway 3 | | |
| --- | --- | --- | --- | --- | --- | --- | --- | --- | --- | --- | --- | --- | --- | --- | --- | --- | --- | --- | --- | --- | --- | --- | --- | --- |
|  |  | β (95%CI) | P | P_FDR |  | β (95%CI) | P | P_FDR |  | β (95%CI) | P | P_FDR |  | β (95%CI) | P | P_FDR |  | β (95%CI) | P | P_FDR |  | β (95%CI) | P | P_FDR |
| NPSs-NFL-ROI-MMSE |  | -1.0511 (-2.0524, -0.7226) | **0.0005** | **0.0012** |  | -0.4561 (-0.9916, 0.0884) | 0.0918 | 0.1721 |  | -0.5956 (-0.9483, -0.3297) | **<0.001** | **<0.001** |  | -0.0584 (-0.1978, 0.0178) | 0.2440 | 0.4067 |  | -0.5200 (-0.8339, -0.2601) | **<0.001** | **<0.001** |  | -0.0171 (-0.1294, 0.0158) | 0.6160 | 0.8035 |
| NPSs-NFL-ROI-ADAS13 |  | 5.2607 (4.0940, 8.4800) | **2.35E-07** | **3.17E-06** |  | 2.9712 (1.3907, 4.4574) | **0.0003** | **0.0022** |  | 2.2915 (1.0197, 3.6394) | **0.0020** | **<0.001** |  | 0.2022 (-0.0834, 0.6552) | 0.2300 | 0.4067 |  | 1.9031 (0.8747, 3.1529) | **<0.001** | **<0.001** |  | 0.1862 (-0.0664, 0.6265) | 0.2320 | 0.6300 |
| NPSs-NFL-ROI-CDRSB |  | 1.1783 (1.0172, 1.7680) | **1.51E-09** | **4.54E-08** |  | 0.7491 (0.4113, 1.0420) | **3.56E-06** | **1.07E-04** |  | 0.4297 (0.2243, 0.7659) | **<0.001** | **<0.001** |  | 0.0405 (-0.0122, 0.1391) | 0.2440 | 0.4067 |  | 0.3603 (0.1801, 0.6261) | **<0.001** | **<0.001** |  | 0.0289 (-0.0047, 0.1142) | 0.2800 | 0.6462 |
| NPSs-NFL-RO-MEM |  | -0.3965 (-0.6231, -0.3116) | **3.17E-07** | **3.17E-06** |  | -0.2293 (-0.3570, -0.1000) | **0.0004** | **0.0026** |  | -0.1674 (-0.2727, -0.0840) | **<0.001** | **<0.001** |  | -0.0155 (-0.0512, 0.0052) | 0.2440 | 0.4067 |  | -0.1382 (-0.2219, -0.0652) | **<0.001** | **<0.001** |  | -0.0137 (-0.0466, 0.0032) | 0.2520 | 0.6300 |
| NPSs-NFL-ROI-LAN |  | -0.2231 (-0.4186, -0.1151) | **0.0039** | **0.0066** |  | -0.0988 (-0.2334, 0.0380) | 0.1756 | 0.2571 |  | -0.1245 (-0.2188, -0.0610) | **<0.001** | **<0.001** |  | -0.0116 (-0.0424, 0.0029) | 0.2440 | 0.4067 |  | -0.1035 (-0.1833, -0.0471) | **<0.001** | **<0.001** |  | -0.0093 (-0.0431, 0.0027) | 0.3340 | 0.6680 |
| NPSs-NFL-ROI-EF |  | -0.2722 (-0.4947, -0.1889) | **0.0011** | **0.0021** |  | -0.1391 (-0.2906, 0.0021) | 0.0760 | 0.1520 |  | -0.1333 (-0.2230, -0.0663) | **<0.001** | **<0.001** |  | -0.0125 (-0.0422, 0.0035) | 0.2440 | 0.4067 |  | -0.1112 (-0.1807, -0.0528) | **<0.001** | **<0.001** |  | -0.0096 (-0.0464, 0.0023) | 0.3180 | 0.6680 |
| HYP-NFL-ROI-MMSE |  | -0.7134 (-1.5139, -0.4847) | **0.0042** | **0.0066** |  | -0.2959 (-0.7418, 0.1199) | 0.1783 | 0.2571 |  | -0.4189 (-0.6772, -0.1905) | **<0.001** | **<0.001** |  | -0.0283 (-0.1305, 0.0349) | 0.4680 | 0.6104 |  | -0.3824 (-0.5880, -0.1807) | **<0.001** | **<0.001** |  | -0.0082 (-0.1022, 0.0125) | 0.6920 | 0.8359 |
| HYP-NFL-ROIADAS13 |  | 3.2648 (2.2431, 5.8124) | **1.17E-04** | **3.50E-04** |  | 1.6616 (0.3405, 2.9803) | **0.0133** | **0.0333** |  | 1.6093 (0.7310, 2.7277) | **<0.001** | **<0.001** |  | 0.0958 (-0.1536, 0.4932) | 0.5580 | 0.6975 |  | 1.4301 (0.6820, 2.1894) | **<0.001** | **<0.001** |  | 0.0834 (-0.1302, 0.4156) | 0.5580 | 0.7609 |
| HYP-NFL-ROI-CDRSB |  | 0.8140 (0.6308, 1.2867) | **5.28E-07** | **3.39E-06** |  | 0.5113 (0.2297, 0.7866) | **0.0001** | **0.0017** |  | 0.3041 (0.1272, 0.5021) | **<0.001** | **<0.001** |  | 0.0200 (-0.0267, 0.0916) | 0.4680 | 0.6104 |  | 0.2702 (0.1286, 0.4302) | **<0.001** | **<0.001** |  | 0.0139 (-0.0184, 0.0787) | 0.4800 | 0.7609 |
| HYP-NFL-ROI-MEM |  | -0.2662 (-0.4466, -0.2209) | **3.55E-05** | **1.18E-04** |  | -0.1494 (-0.2389, -0.0591) | **0.0050** | **0.0212** |  | -0.1173 (-0.1836, -0.0460) | **<0.001** | **<0.001** |  | -0.0076 (-0.0332, 0.0101) | 0.4680 | 0.6104 |  | -0.1032 (-0.1569, -0.0500) | **<0.001** | **<0.001** |  | -0.0065 (-0.0333, 0.0083) | 0.4720 | 0.7609 |
| HYP-NFL-ROI-LAN |  | -0.1793 (-0.3308, -0.1161) | **0.0048** | **0.0071** |  | -0.0953 (-0.1973, 0.0091) | 0.1074 | 0.1895 |  | -0.0845 (-0.1451, -0.0362) | **<0.001** | **<0.001** |  | -0.0055 (-0.0270, 0.0068) | 0.4680 | 0.6104 |  | -0.0745 (-0.1218, -0.0349) | **<0.001** | **<0.001** |  | -0.0044 (-0.0319, 0.0052) | 0.5080 | 0.7609 |
| HYP-NFL-ROI-EF |  | -0.1825 (-0.3686, -0.1234) | **0.0078** | **0.0106** |  | -0.0899 (-0.2132, 0.0295) | 0.1582 | 0.2571 |  | -0.0930 (-0.1635, -0.0378) | **<0.001** | **<0.001** |  | -0.0061 (-0.0277, 0.0073) | 0.4680 | 0.6104 |  | -0.0824 (-0.1353, -0.0391) | **<0.001** | **<0.001** |  | -0.0045 (-0.0280, 0.0055) | 0.5220 | 0.7609 |
| PSYNFL-ROI-MMSE |  | -0.7391 (-1.9307, -0.1277) | 0.0670 | 0.0718 |  | -0.2166 (-1.0167, 0.3696) | 0.5366 | 0.5551 |  | -0.5226 (-1.0106, -0.0097) | **0.0480** | 0.0576 |  | -0.0248 (-0.2053, 0.1070) | 0.7600 | 0.7862 |  | -0.4912 (-0.9329, -0.1166) | **0.0220** | **0.0287** |  | -0.0066 (-0.1029, 0.0386) | 0.8520 | 0.8520 |
| PSY-NFL-ROI-ADAS13 |  | 3.3523 (0.4928, 7.2048) | **0.0160** | **0.0208** |  | 1.4503 (-0.4233, 3.7403) | 0.1800 | 0.2571 |  | 1.9017 (-0.2477, 3.9007) | 0.1000 | 0.1000 |  | 0.0584 (-0.4895, 0.6600) | 0.8060 | 0.8060 |  | 1.7959 (0.2330, 3.4984) | **0.0340** | **0.0425** |  | 0.0474 (-0.4097, 0.5102) | 0.8040 | 0.8359 |
| PSY-NFL-ROI-CDRSB |  | 0.8175 (0.4736, 1.6583) | **0.0022** | **0.0041** |  | 0.4296 (0.0392, 0.9324) | **0.0441** | 0.0945 |  | 0.3880 (-0.0079, 0.8043) | 0.0640 | 0.0738 |  | 0.0181 (-0.0733, 0.1469) | 0.7600 | 0.7862 |  | 0.3587 (0.0887, 0.7305) | **0.0220** | **0.0287** |  | 0.0112 (-0.0512, 0.0997) | 0.7920 | 0.8359 |
| PSY-NFL-ROI-MEM |  | -0.2431 (-0.5121, -0.0502) | **0.0209** | **0.0261** |  | -0.0948 (-0.2415, 0.0836) | 0.2665 | 0.3454 |  | -0.1483 (-0.2910, 0.0070) | 0.0740 | 0.0793 |  | -0.0069 (-0.0530, 0.0298) | 0.7600 | 0.7862 |  | -0.1362 (-0.2704, -0.0298) | **0.0220** | **0.0287** |  | -0.0053 (-0.0473, 0.0235) | 0.7700 | 0.8359 |
| PSY-NFL-ROI-LAN |  | -0.1888 (-0.3973, 0.0175) | 0.0666 | 0.0718 |  | -0.0833 (-0.2445, 0.0842) | 0.3773 | 0.4192 |  | -0.1055 (-0.2228, 0.0027) | 0.0840 | 0.0869 |  | -0.0049 (-0.0392, 0.0212) | 0.7600 | 0.7862 |  | -0.0970 (-0.2050, -0.0244) | **0.0220** | **0.0287** |  | -0.0036 (-0.0402, 0.0144) | 0.7940 | 0.8359 |
| PSY-NFL-ROI-EF |  | -0.1232 (-0.4350, 0.0589) | 0.2681 | 0.2681 |  | -0.0051 (-0.2282, 0.2209) | 0.9601 | 0.9601 |  | -0.1182 (-0.2373, 0.0028) | 0.0700 | 0.0778 |  | -0.0055 (-0.0432, 0.0239) | 0.7600 | 0.7862 |  | -0.1090 (-0.2099, -0.0203) | **0.0220** | **0.0287** |  | -0.0037 (-0.0412, 0.0154) | 0.8080 | 0.8359 |
| AFF-NFL-ROI-MMSE |  | -0.7964 (-2.0231, -0.5257) | **0.0251** | **0.0301** |  | -0.3536 (-1.0393, 0.1844) | 0.2520 | 0.3436 |  | -0.4433 (-0.8968, -0.0571) | **0.0320** | **0.0464** |  | -0.0970 (-0.2845, 0.0023) | 0.1200 | 0.3273 |  | -0.3231 (-0.7051, 0.0234) | 0.0620 | 0.0641 |  | -0.0233 (-0.1896, 0.0311) | 0.5260 | 0.7609 |
| AFF-NFL-ROI-ADAS13 |  | 4.1815 (3.3910, 7.9332) | **0.0006** | **0.0012** |  | 2.3905 (0.8430, 3.9528) | **0.0114** | **0.0327** |  | 1.7929 (0.2179, 3.6467) | **0.0360** | **0.0470** |  | 0.3462 (-0.0626, 1.0198) | 0.1560 | 0.3900 |  | 1.1764 (-0.1919, 2.5373) | 0.0740 | 0.0740 |  | 0.2703 (-0.0573, 0.7825) | 0.1580 | 0.6300 |
| AFF-NFL-ROI-CDRSB |  | 1.0346 (0.8452, 1.8021) | **8.47E-06** | **3.18E-05** |  | 0.6923 (0.2522, 1.0509) | **0.0002** | **0.0020** |  | 0.3427 (0.0284, 0.6822) | **0.0380** | **0.0475** |  | 0.0702 (-0.0030, 0.2067) | 0.1200 | 0.3273 |  | 0.2339 (-0.0238, 0.5024) | 0.0620 | 0.0641 |  | 0.0387 (-0.0049, 0.1512) | 0.1920 | 0.6300 |
| AFF-NFL-ROI-MEM |  | -0.3275 (-0.5963, -0.2915) | **0.0004** | **9.35E-04** |  | -0.1930 (-0.3104, -0.0684) | **0.0098** | **0.0327** |  | -0.1347 (-0.2584, -0.0150) | **0.0340** | **0.0464** |  | -0.0266 (-0.0778, 0.0010) | 0.1200 | 0.3273 |  | -0.0886 (-0.1865, 0.0092) | 0.0620 | 0.0641 |  | -0.0196 (-0.0662, 0.0007) | 0.1420 | 0.6300 |
| AFF-NFL-ROI-LAN |  | -0.1640 (-0.4023, -0.0670) | 0.0711 | 0.0736 |  | -0.0661 (-0.1892, 0.0868) | 0.4270 | 0.4575 |  | -0.0981 (-0.2044, -0.0117) | **0.0340** | **0.0464** |  | -0.0194 (0.0584, 0.0007) | 0.1200 | 0.3273 |  | -0.0647 (-0.1482, -0.0042) | 0.0620 | 0.0641 |  | -0.0139 (-0.0614, 0.0016) | 0.2400 | 0.6300 |
| AFF-NFL-ROI-EF |  | -0.2007 (-0.5141, -0.1262) | **0.0403** | **0.0466** |  | -0.0951 (-0.2803, 0.0965) | 0.2878 | 0.3454 |  | -0.1057 (-0.2169, -0.0156) | **0.0320** | **0.0464** |  | -0.0211 (-0.0616, 0.0006) | 0.1200 | 0.3273 |  | -0.0705 (-0.1564, 0.0007) | 0.0620 | 0.0641 |  | -0.0141 (-0.0575, 0.0029) | 0.2320 | 0.6300 |
| APA-NFL-ROI-MMSE |  | -1.1211 (-2.3609, -0.7240) | **0.0030** | **0.0052** |  | -0.3063 (-0.9636, 0.4347) | 0.3666 | 0.4192 |  | -0.8151 (-1.3330, -0.3965) | **<0.001** | **<0.001** |  | -0.1037 (-0.3385, -0.0063) | 0.0840 | 0.3273 |  | -0.6827 (-1.1182, -0.3580) | **<0.001** | **<0.001** |  | -0.0287 (-0.1746, 0.0349) | 0.5420 | 0.7609 |
| APA-NFL-ROI-ADAS13 |  | 5.9415 (4.0680, 9.5879) | **2.98E-06** | **1.49E-05** |  | 2.6061 (0.7173, 4.1867) | **0.0120** | **0.0327** |  | 3.3372 (1.4172, 5.5150) | **<0.001** | **<0.001** |  | 0.3850 (0.0354, 1.1326) | 0.0640 | 0.3273 |  | 2.6066 (1.3727, 4.5755) | **<0.001** | **<0.001** |  | 0.3456 (0.0190, 0.9399) | 0.0640 | 0.6300 |
| APA-NFL-ROI-CDRSB |  | 1.2307 (0.8908, 2.1161) | **5.65E-07** | **3.39E-06** |  | 0.6283 (0.1105, 1.1296) | **0.0022** | **0.0110** |  | 0.6027 (0.2779, 1.0507) | **<0.001** | **<0.001** |  | 0.0731 (0.0047, 0.2505) | 0.0840 | 0.3273 |  | 0.4814 (0.2487, 0.8645) | **<0.001** | **<0.001** |  | 0.0482 (-0.0013, 0.1514) | 0.1400 | 0.6300 |
| APA-NFL-ROI-MEM |  | -0.4449 (-0.6802, -0.3656) | **4.58E-06** | **1.96E-05** |  | -0.2126 (-0.3203, -0.0834) | **0.0096** | **0.0327** |  | -0.2324 (-0.3918, -0.1060) | **<0.001** | **<0.001** |  | -0.0276 (-0.0892, -0.0016) | 0.0840 | 0.3273 |  | -0.1815 (-0.3153, -0.0955) | **<0.001** | **<0.001** |  | -0.0234 (-0.0795, -0.0003) | 0.1060 | 0.6300 |
| APA-NFL-ROI-LAN |  | -0.3637 (-0.5912, -0.2364) | **1.39E-04** | **3.80E-04** |  | -0.2026 (-0.3690, -0.0074) | **0.0258** | 0.0596 |  | -0.1612 (-0.2990, -0.0709) | **<0.001** | **<0.001** |  | -0.0192 (-0.0634, -0.0011) | 0.0840 | 0.3273 |  | -0.1262 (-0.2451, -0.0570) | **<0.001** | **<0.001** |  | -0.0159 (-0.0651, 0.0027) | 0.1980 | 0.6300 |
| APA-NFL-ROI-EF |  | -0.2912 (-0.5629, -0.1612) | **0.0050** | **0.0072** |  | -0.1057 (-0.2973, 0.1014) | 0.2822 | 0.3454 |  | -0.1856 (-0.3188, -0.0839) | **<0.001** | **<0.001** |  | -0.0223 (-0.0779, -0.0013) | 0.0840 | 0.3273 |  | -0.1467 (-0.2576, -0.0773) | **<0.001** | **<0.001** |  | -0.0166 (-0.0631, 0.0009) | 0.1800 | 0.6300 |

Mediation analysis based on structural equation modeling identified three potential mediation pathways: (1) *NPSs → plasma NFL→ AD-signature ROI → cognition*, (2) *NPSs→ AD-signature ROI → cognition*, and (3) *NPSs → plasma NFL → cognition*. **All analyses were adjusted for age, sex, education, and APOE ε4 status, with intracranial volume additionally included as a covariate in models involving brain structure measures.** Multiple testing significance was corrected using FDR. Significant at the level of *P* < 0.05 were shown in bold. NPSs neuropsychiatric symptoms; HYP hyperactivity; PSY psychosis; AFF affective; APA apathy; MMSE Mini-Mental State Examination; ADAS Alzheimer’s disease Assessment Scale; CDRSB Clinical Dementia Rating Sum of Boxes; MEM Memory function; LAN Language; EF Executive function; NFL neurofilament light chain; ROI region of interest; FDR false discovery rate; CI Confidence Interval.

**Table S12. Sequential mediation models linking NPSs to the slope of cognition in the overall ADNI cohort.**

| Models |  | Total Effect | | |  | Direct Effect | | |  | Total Indirect Effect | | |  | Pathway 1 | | |  | Pathway 2 | | |  | Pathway 3 | | |  |
| --- | --- | --- | --- | --- | --- | --- | --- | --- | --- | --- | --- | --- | --- | --- | --- | --- | --- | --- | --- | --- | --- | --- | --- | --- | --- |
|  |  | β (95%CI) | P | P_FDR |  | β (95%CI) | P | P_FDR |  | β (95%CI) | P | P_FDR |  | β (95%CI) | P | P_FDR |  | β (95%CI) | P | P_FDR |  | β (95%CI) | P | P_FDR |  |
| NPSs-NFL-ROI-slope of MMSE |  | -0.7144 (-1.1072, -0.5076) | **4.23E-08** | **6.35E-07** |  | -0.3052 (-0.5388, -0.0607) | **0.0039** | **0.0391** |  | -0.4114 (-0.6068, -0.2472) | **<0.001** | **<0.001** |  | -0.0601 (-0.1273, -0.0092) | **0.0340** | 0.0975 |  | -0.3278 (-0.4955, -0.1817) | **<0.001** | **<0.001** |  | -0.0235 (-0.0789, -0.0001) | 0.1160 | 0.2440 |  |
| NPSs-NFL-ROI-slope of ADAS13 |  | 1.2075 (0.8349, 1.9367) | **8.08E-07** | **6.06E-06** |  | 0.4178 (-0.0805, 0.8351) | **0.0325** | 0.1408 |  | 0.7935 (0.5111, 1.2046) | **<0.001** | **<0.001** |  | 0.1168 (0.0208, 0.2622) | **0.0400** | 0.0975 |  | 0.6452 (0.3536, 0.9609) | **<0.001** | **<0.001** |  | 0.0315 (-0.0160, 0.1117) | 0.2260 | 0.2904 |  |
| NPSs-NFL-ROI-slope of CDRSB |  | 0.4729 (0.3553, 0.7194) | **2.06E-08** | **6.17E-07** |  | 0.2007 (0.0575, 0.3462) | **0.0026** | **0.0383** |  | 0.2738 (0.1707, 0.3923) | **<0.001** | **<0.001** |  | 0.0389 (0.0074, 0.0820) | **0.0300** | 0.0975 |  | 0.2146 (0.1220, 0.3164) | **<0.001** | **<0.001** |  | 0.0203 (0.0030, 0.0604) | **0.0480** | 0.1600 |  |
| NPSs-NFL-ROI-slope of MEM |  | 6.95E-16(-1.25E-15,3.08E-15) | 0.5680 | 0.7712 |  | 9.42E-19(-2.15E-15,2.56E-15) | 0.9994 | 0.9994 |  | 6.92E-16(3.20E-17,1.66E-15) | 0.0600 | 0.1385 |  | 1.45E-16(2.31E-17,5.12E-16) | 0.0500 | 0.0975 |  | 8.39E-16(2.55E-16,1.85E-15) | **0.0080** | **0.0240** |  | -2.91E-16(-9.18E-16, -3.16E-17) | 0.0640 | 0.1600 |  |
| NPSs-NFL-ROI-slope of LAN |  | 1.12E-04 (-3.00E-04,4.42E-04) | 0.5701 | 0.7712 |  | 3.94E-05 (-0.0003, 0.0004) | 0.8460 | 0.9994 |  | 7.17E-05 (-4.51E-05,2.34E-04) | 0.2680 | 0.4020 |  | 1.88E-05 (1.48E-06, 0.0001) | 0.0820 | 0.1096 |  | 0.0001 (1.33E-05, 0.0003) | **0.0440** | 0.0695 |  | -0.0001 (-0.0002, -6.88E-06) | **0.0480** | 0.1600 |  |
| NPSs-NFL-ROI-slope of EF |  | -4.09E-05(-5.97E-04,6.86E-04) | 0.8996 | 0.9307 |  | -0.0002 (0.0008, 0.0006) | 0.5642 | 0.9708 |  | 1.53E-04 (-3.18E-05,4.24E-04) | 0.1240 | 0.2100 |  | 3.07E-05 (2.40E-06, 0.0001) | 0.0880 | 0.1100 |  | 0.0002 (2.16E-05, 0.0004) | 0.0500 | 0.0750 |  | -0.0001 (-0.0002, 9.90E-06) | 0.2120 | 0.2904 |  |
| HYP-NFL-ROI-slope of MMSE |  | -0.4484 (-0.7362, -0.3012) | **6.21E-05** | **2.44E-04** |  | -1.94E-04 (-8.44E-04,5.64E-04) | 0.1597 | 0.4355 |  | -0.3245 (-0.4681, -0.1956) | **<0.001** | **<0.001** |  | -0.0500 (-0.1078, 0.0004) | 0.0620 | 0.1080 |  | 1.09E-04(1.33E-05,2.69E-04) | **<0.001** | **<0.001** |  | -5.61E-05(-1.59E-04, -6.88E-06) | 0.1400 | 0.2625 |  |
| HYP-NFL-ROI-slope of ADAS13 |  | 0.7590 (0.4430, 1.2542) | **2.95E-04** | **8.05E-04** |  | -0.1258 (-0.3445, 0.0533) | 0.3870 | 0.7255 |  | 0.6193 (0.3623,0.8899) | **<0.001** | **<0.001** |  | 0.0959 (0.0076, 0.2251) | 0.0520 | 0.0975 |  | 1.78E-04(2.16E-05,4.41E-04) | **<0.001** | **<0.001** |  | -5.57E-05 (-2.14E-04, 9.90E-06) | 0.2400 | 0.2904 |  |
| HYP-NFL-ROI-slope of CDRSB |  | 0.3591 (0.2556, 0.5513) | **6.15E-07** | **6.06E-06** |  | 0.1426 (-0.2173, 0.4674) | **0.0081** | 0.0605 |  | 0.2118 (0.1362, 0.3075) | **<0.001** | **<0.001** |  | 0.0318 (0.0020, 0.0723) | 0.0440 | 0.0975 |  | -0.2562 (-0.3894, -0.1453) | **<0.001** | **<0.001** |  | -0.0183 (-0.0643, 0.0008) | 0.0640 | 0.1600 |  |
| HYP-NFL-ROI-slope of MEM |  | 8.46E-16 (-7.04E-16,2.83E-15) | 0.4096 | 0.7228 |  | 0.1485 (0.0382, 0.2807) | 0.7264 | 0.9994 |  | 4.76E-16 (-7.94E-17,1.19E-15) | 0.1120 | 0.2100 |  | 1.27E-16(1.29E-17,3.76E-16) | 0.0440 | 0.0975 |  | 0.4990 (0.2916, 0.7586) | **0.0120** | **0.0300** |  | 0.0245 (-0.011, 0.1006) | 0.0580 | 0.1600 |  |
| HYP-NFL-ROI-slope of LAN |  | 6.09E-06 (-3.46E-04,2.68E-04) | 0.9707 | 0.9707 |  | 3.68E-16 (-1.28E-15,2.41E-15) | 0.7748 | 0.9994 |  | 5.43E-05 (-4.40E-05,1.76E-04) | 0.2960 | 0.4064 |  | 1.80E-05(1.54E-06,5.84E-05) | 0.0700 | 0.1080 |  | 0.1641 (0.0941, 0.2453) | **0.0340** | **0.0600** |  | 0.0160 (0.0014, 0.0511) | 0.0400 | 0.1600 |  |
| HYP-NFL-ROI-slope of EF |  | -1.47E-04 (-6.51E-04,4.27E-04) | 0.5912 | 0.7712 |  | -4.86E-05 (-3.94E-04,2.76E-04) | 0.3475 | 0.7255 |  | 1.18E-04 (-2.83E-05,3.18E-04) | 0.1400 | 0.2211 |  | 2.87E-05(1.96E-06,9.55E-05) | 0.0760 | 0.1086 |  | 6.13E-16(1.61E-16,1.39E-15) | **0.0400** | 0.0667 |  | -2.64E-16(-8.28E-16, -2.46E-17) | 0.2100 | 0.2904 |  |
| PSY-NFL-ROI-slope of MMSE |  | -0.2514 (-0.8448, 0.0396) | 0.1512 | 0.3025 |  | -2.65E-04 (-8.33E-04,3.06E-04) | 0.9096 | 0.9994 |  | -0.2369 (-0.4906, 0.0612) | 0.1060 | 0.2100 |  | -0.0795 (-0.2089, 0.0147) | 0.1580 | 0.1823 |  | 8.69E-05(1.53E-05,2.26E-04) | 0.1900 | 0.2192 |  | -5.06E-05(-1.45E-04, -8.04E-06) | 0.2340 | 0.2904 |  |
| PSY-NFL-ROI-slope of ADAS13 |  | 0.4436 (-0.0809, 1.4257) | 0.1801 | 0.3376 |  | -0.0153 (-0.3562, 0.3091) | 0.9098 | 0.9994 |  | 0.4164 (-0.0798, 0.9330) | 0.1260 | 0.2100 |  | 0.1515 (-0.0234, 0.3956) | 0.1760 | 0.1956 |  | 1.39E-04(2.31E-05,3.59E-04) | 0.2560 | 0.2648 |  | -4.98E-05 (-1.93E-04, 9.80E-06) | 0.3680 | 0.3807 |  |
| PSY-NFL-ROI-slope of CDRSB |  | 0.2522 (0.0209, 0.5644) | 0.0260 | 0.0599 |  | 0.0284 (-0.6189, 0.5630) | 0.2701 | 0.6753 |  | 0.1593 (-0.0288, 0.3325) | 0.0880 | 0.1886 |  | 0.0515 (-0.0083,0.1342) | 0.1580 | 0.1823 |  | -0.1304 (-0.3219, 0.0837) | 0.1780 | 0.2136 |  | -0.0270 (-0.1239, 0.0014) | 0.1800 | 0.2904 |  |
| PSY-NFL-ROI-slope of MEM |  | 3.25E-16 (-2.82E-15,3.47E-15) | 0.8418 | 0.9307 |  | 0.0935 (-0.1415, 0.2566) | 0.9470 | 0.9994 |  | 2.17E-16 (-3.87E-16,1.04E-15) | 0.4620 | 0.5544 |  | 1.17E-16(-5.41E-17,5.44E-16) | 0.3280 | 0.3514 |  | 0.2288 (-0.1824, 0.6068) | 0.2280 | 0.2533 |  | 0.0362 (-0.0149, 0.1610) | 0.3440 | 0.3686 |  |
| PSY-NFL-ROI-slope of LAN |  | 7.36E-05 (-5.15E-04,4.94E-04) | 0.7792 | 0.8991 |  | 1.07E-16 (-3.19E-15,3.41E-15) | 0.8230 | 0.9994 |  | 1.51E-05 (-7.39E-05,1.33E-04) | 0.7620 | 0.7883 |  | 1.55E-05(-4.50E-06,8.41E-05) | 0.3460 | 0.3579 |  | 0.0848 (-0.0382, 0.2254) | 0.2500 | 0.2648 |  | 0.0230 (-0.0023, 0.0762) | 0.3300 | 0.3667 |  |
| PSY-NFL-ROI-slope of EF |  | 8.75E-04 (1.52E-04, 0.0018) | **0.0428** | 0.0918 |  | 5.84E-05 (-4.89E-04,5.43E-04) | 0.0524 | 0.1965 |  | 3.59E-05 (-7.52E-05,2.31E-04) | 0.6200 | 0.6889 |  | 2.13E-05(-8.72E-06,1.15E-04) | 0.3720 | 0.3720 |  | 3.24E-16(-1.29E-16,1.29E-15) | 0.2880 | 0.2880 |  | -2.24E-16 (-1.09E-15, 1.03E-16) | 0.3920 | 0.3920 |  |
| AFF-NFL-ROI-slope of MMSE |  | -0.5782 (-1.0177, -0.3415) | **6.50E-05** | **2.44E-04** |  | -0.1960 (-0.4788, 0.0946) | 0.0885 | 0.2654 |  | -0.3782 (-0.6035, -0.1773) | **<0.001** | **<0.001** |  | -0.1022 (-0.1908, -0.0479) | **0.0020** | **0.0150** |  | -0.2419 (-0.4411, -0.0673) | **0.0080** | **0.0240** |  | -0.0342 (-0.1146, 0.0010) | 0.1220 | 0.2440 |  |
| AFF-NFL-ROI-slope of ADAS13 |  | 0.9364 (0.4825, 1.7542) | **5.86E-04** | **0.0015** |  | 0.2223 (-0.3050, 0.7199) | 0.2962 | 0.6836 |  | 0.7070 (0.3115, 1.1371) | **<0.001** | **<0.001** |  | 0.1974 (0.0821, 0.3690) | **0.0020** | **0.0150** |  | 0.4629 (0.1164, 0.8241) | **0.0160** | **0.0343** |  | 0.0467 (-0.0320, 0.1578) | 0.2620 | 0.3023 |  |
| AFF-NFL-ROI-slope of CDRSB |  | 0.3865 (0.2277, 0.6402) | **3.73E-05** | **1.87E-04** |  | 0.1275 (-0.0697, 0.2903) | 0.0784 | 0.2612 |  | 0.2563 (0.1315, 0.3984) | **<0.001** | **<0.001** |  | 0.0670 (0.0305, 0.1282) | **<0.001** | **<0.001** |  | 0.1585 (0.0449, 0.2721) | **0.0080** | **0.0240** |  | 0.0308 (0.0053, 0.0883) | **0.0280** | 0.1600 |  |
| AFF-NFL-ROI-slope of MEM |  | -8.35E-16(-2.59E-15,1.86E-15) | 0.5341 | 0.7712 |  | -1.19E-15(-3.58E-15,1.50E-15) | 0.3860 | 0.7255 |  | 3.64E-16(-3.17E-16,1.36E-15) | 0.3500 | 0.4565 |  | 2.85E-16(9.01E-17,7.83E-16) | **0.0020** | **0.0150** |  | 6.00E-16(1.37E-16,1.52E-15) | **0.0200** | **0.0400** |  | -5.21E-16(-1.30E-15, -1.03E-16) | **0.0420** | 0.1600 |  |
| AFF-NFL-ROI-slope of LAN |  | 1.17E-04 (-2.81E-04,5.14E-04) | 0.5880 | 0.7712 |  | 0.0001 (-0.0003, 0.0005) | 0.5825 | 0.9708 |  | -1.28E-06 (-1.10E-04,1.30E-04) | 1.0080 | 1.0000 |  | 3.46E-05(5.96E-06,0.0001) | **0.0460** | 0.0975 |  | 0.0001 (6.74E-06,0.0002) | 0.0600 | 0.0818 |  | -0.0001 (-0.0002, -3.51E-05) | **0.0080** | 0.1600 |  |
| AFF-NFL-ROI-slope of EF |  | 5.68E-05 (-4.74E-04,7.89E-04) | 0.8739 | 0.9307 |  | -1.51E-06 (-0.0007, 0.0007) | 0.9967 | 0.9994 |  | 6.05E-05 (-1.15E-04,2.63E-04) | 0.5380 | 0.6208 |  | 0.0001 (4.86E-06,0.0002) | 0.0720 | 0.1080 |  | 0.0001 (4.79E-06,0.0003) | 0.0900 | 0.1125 |  | -0.0001 (-0.0003, 2.75E-05) | 0.1860 | 0.2904 |  |
| APA-NFL-ROI-slope of MMSE |  | -0.6376 (-1.1458, -0.3216) | **1.30E-04** | **3.89E-04** |  | -0.2808 (-0.6020, 0.0481) | **0.0313** | 0.1408 |  | -0.3580 (-0.6400, -0.0981) | **<0.001** | **<0.001** |  | -0.0829 (-0.1831, -0.0201) | **0.0120** | 0.0514 |  | -0.2470 (-0.4803, -0.0272) | **0.0100** | **0.0273** |  | -0.0281 (-0.0904, -0.0001) | 0.1160 | 0.2440 |  |
| APA-NFL-ROI-slope of ADAS13 |  | 1.2604 (0.5864, 2.3130) | **8.04E-05** | **2.68E-04** |  | 0.5284 (0.0435, 1.1034) | **0.0328** | 0.1408 |  | 0.7359 (0.2584, 1.3913) | **<0.001** | **<0.001** |  | 0.1795 (0.0487, 0.3548) | **0.0100** | 0.0500 |  | 0.5147 (0.0918,1.0705) | **0.0140** | **0.0323** |  | 0.0417 (-0.0305, 0.1467) | 0.2420 | 0.2904 |  |
| APA-NFL-ROI-slope of CDRSB |  | 0.4987 (0.2887, 0.8289) | **3.38E-06** | **2.03E-05** |  | 0.2613 (0.0713, 0.4568) | **0.0014** | **0.0383** |  | 0.2383 (0.0795, 0.4086) | **<0.001** | **<0.001** |  | 0.0536 (0.0138, 0.1085) | **0.0080** | **0.0480** |  | 0.1601 (0.0181,0.2956) | **0.0080** | **0.0240** |  | 0.0245 (0.0041, 0.0729) | **0.0320** | 0.1600 |  |
| APA-NFL-ROI-slope of MEM |  | 6.79E-16 (-1.41E-15,3.28E-15) | 0.6528 | 0.7833 |  | 2.46E-16 (-1.88E-15,2.56E-15) | 0.8719 | 0.9994 |  | 4.30E-16(-2.62E-16,1.42E-15) | 0.2980 | 0.4064 |  | 2.14E-16(4.27E-17,5.88E-16) | **0.0340** | 0.0975 |  | 6.46E-16(1.03E-16, 1.67E-15) | **0.0280** | 0.0525 |  | -4.29E-16(-1.12E-15, -5.64E-17) | **0.0460** | 0.1600 |  |
| APA-NFL-ROI-slope of LAN |  | 0.0001 (-0.0003, 0.0005) | 0.6233 | 0.7791 |  | 0.0001 (-0.0003, 0.0005) | 0.7165 | 0.9994 |  | 2.94E-05 (-8.33E-05,2.04E-04) | 0.6620 | 0.7093 |  | 2.80E-05 (2.88E-06, 0.0001) | 0.0680 | 0.1080 |  | 0.0001 (6.43E-06, 0.0003) | 0.0580 | 0.0818 |  | -0.0001 (-0.0002, -1.48E-05) | **0.0280** | 0.1600 |  |
| APA-NFL-ROI-slope of EF |  | 0.0002 (-0.0006, 0.0012) | 0.5465 | 0.7712 |  | 0.0002 (-0.0007, 0.0011) | 0.6962 | 0.9994 |  | 8.21E-05 (-9.76E-05,3.38E-04) | 0.4080 | 0.5100 |  | 4.10E-05 (8.98E-07, 0.0001) | 0.0840 | 0.1096 |  | 0.0001 (6.55E-06, 0.0004) | 0.0740 | 0.0965 |  | -0.0001 (-0.0003, 1.75E-05) | 0.1900 | 0.2904 |  |

Mediation analysis based on structural equation modeling identified three potential mediation pathways: (1) *NPSs → plasma NFL→ AD-signature ROI → cognition*, (2) *NPSs→ AD-signature ROI → cognition*, and (3) *NPSs → plasma NFL → cognition*. **All analyses were adjusted for age, sex, education, and APOE ε4 status, with intracranial volume additionally included as a covariate in models involving brain structure measures.** Multiple testing significance was corrected using FDR. Significant at the level of *P* < 0.05 were shown in bold. NPSs neuropsychiatric symptoms; HYP hyperactivity; PSY psychosis; AFF affective; APA apathy; MMSE Mini-Mental State Examination; ADAS Alzheimer’s disease Assessment Scale; CDRSB Clinical Dementia Rating Sum of Boxes; MEM Memory function; LAN Language; EF Executive function; NFL neurofilament light chain; ROI region of interest; FDR false discovery rate; CI Confidence Interval.

**Table S13. Sequential mediation models linking NPSs to the slope of cognition in the ADNI female subsample.**

| Models |  | Total Effect | | |  | Direct Effect | | |  | Total Indirect Effect | | |  | Pathway 1 | | |  | Pathway 2 | | |  | Pathway 3 | | |  |
| --- | --- | --- | --- | --- | --- | --- | --- | --- | --- | --- | --- | --- | --- | --- | --- | --- | --- | --- | --- | --- | --- | --- | --- | --- | --- |
|  |  | β (95%CI) | P | P_FDR |  | β (95%CI) | P | P_FDR |  | β (95%CI) | P | P_FDR |  | β (95%CI) | P | P_FDR |  | β (95%CI) | P | P_FDR |  | β (95%CI) | P | P_FDR |  |
| NPSs-NFL-ROI-slope of MMSE |  | -0.9442 (-1.6021, -0.5093) | **3.88E-04** | **0.0108** |  | -0.2925 (-0.7802, 0.1979) | 0.1690 | 0.7769 |  | -0.6534 (-1.1131, -0.3232) | **<0.001** | **<0.001** |  | -0.1665 (-0.3897, -0.0351) | **0.0280** | 0.0560 |  | -0.4824 (-0.9952, -0.1844) | **0.0040** | **0.0300** |  | -0.0045 (-0.1184, 0.1574) | 0.8820 | 0.9240 |  |
| NPSs-NFL-ROI-slope of ADAS13 |  | 1.4373 (0.7152, 2.6258) | **0.0030** | **0.0149** |  | 0.1304 (-0.7007, 0.8479) | 0.7173 | 0.8479 |  | 1.3104 (0.6635, 2.1434) | **<0.001** | **<0.001** |  | 0.3332 (0.0650, 0.7435) | **0.0280** | 0.0560 |  | 0.9657 (0.3562, 1.7503) | **0.0040** | **0.0300** |  | 0.0115 (-0.2256, 0.2027) | 0.8600 | 0.9240 |  |
| NPSs-NFL-ROI-slope of CDRSB |  | 0.5507 (0.2849, 0.9339) | **0.0011** | **0.0108** |  | 0.1119 (-0.1500, 0.3991) | 0.3889 | 0.8479 |  | 0.4402 (0.2181, 0.7009) | **<0.001** | **<0.001** |  | 0.1062 (0.0216, 0.2492) | **0.0280** | 0.0560 |  | 0.3078 (0.1125, 0.5767) | **0.0040** | **0.0300** |  | 0.0261 (-0.0323, 0.1295) | 0.4300 | 0.8250 |  |
| NPSs-NFL-ROI-slope of MEM |  | 3.27E-15 (-2.00E-15, 7.25E-15) | 0.1830 | 0.3922 |  | 1.51E-15 (-3.64E-15, 6.51E-15) | 0.5535 | 0.8479 |  | 1.76E-15 (-6.58E-17, 4.42E-15) | 0.0760 | 0.1754 |  | 5.70E-16 (3.58E-17, 1.76E-15) | 0.0800 | 0.1025 |  | 2.04E-15 (5.13E-16, 4.85E-15) | **0.0300** | 0.0529 |  | -8.47E-16 (-3.21E-15, -3.44E-17) | 0.1100 | 0.3050 |  |
| NPSs-NFL-ROI-slope of LAN |  | 6.89E-04 (-1.59E-04, 0.0013) | 0.0704 | 0.1761 |  | 4.38E-04 (-2.92E-04, 0.0013) | 0.2651 | 0.8479 |  | 2.51E-04 (-6.87E-05, 7.19E-04) | 0.1440 | 0.2467 |  | 8.93E-05 (1.12E-05, 3.43E-04) | 0.0680 | 0.1025 |  | 3.19E-04 (5.77E-05, 7.88E-04) | **0.0220** | **0.0480** |  | -1.58E-04 (-4.88E-04, -9.16E-06) | 0.0780 | 0.3050 |  |
| NPSs-NFL-ROI-slope of EF |  | -4.03E-05 (-0.0014, 0.0011) | 0.9475 | 0.9475 |  | -3.77E-04 (-0.0019, 0.0011) | 0.5570 | 0.8479 |  | 3.36E-04 (-9.42E-05, 9.32E-04) | 0.1480 | 0.2467 |  | 1.21E-04 (7.72E-06, 4.51E-04) | 0.0720 | 0.1025 |  | 4.34E-04 (8.36E-05, 1.04E-03) | **0.0220** | **0.0480** |  | -2.19E-04 (-7.93E-04, 4.52E-06) | 0.1200 | 0.3050 |  |
| HYP-NFL-ROI-slope of MMSE |  | -0.5232 (-1.0998, -0.2975) | **0.0284** | 0.0888 |  | -0.0911 (-0.5107, 0.2297) | 0.6161 | 0.8479 |  | -0.4309 (-0.7665, -0.1714) | **<0.001** | **<0.001** |  | -0.1210 (-0.3156, -0.0050) | 0.0820 | 0.1025 |  | -0.3059 (-0.6760, -0.0828) | **0.0060** | **0.0300** |  | -0.0039 (-0.1067, 0.0736) | 0.8420 | 0.9240 |  |
| HYP-NFL-ROI-slope of ADAS13 |  | 0.9505 (0.5933, 1.8897) | **0.0272** | 0.0888 |  | 0.1171 (-0.5256, 0.6737) | 0.7022 | 0.8479 |  | 0.8311 (0.3246, 1.4395) | **<0.001** | **<0.001** |  | 0.2335 (-0.0021, 0.5684) | 0.0820 | 0.1025 |  | 0.5901 (0.1579, 1.2092) | **0.0060** | **0.0300** |  | 0.0076 (-0.1103, 0.1771) | 0.8300 | 0.9240 |  |
| HYP-NFL-ROI-slope of CDRSB |  | 0.4608 (0.3216, 0.7658) | **0.0020** | **0.0120** |  | 0.1844 (-0.0096, 0.4155) | 0.0936 | 0.7769 |  | 0.2756 (0.1100, 0.4854) | **<0.001** | **<0.001** |  | 0.0734 (0.0046, 0.1993) | 0.0820 | 0.1025 |  | 0.1855 (0.0504, 0.3886) | **0.0060** | **0.0300** |  | 0.0167 (-0.0150, 0.1027) | 0.4200 | 0.8250 |  |
| HYP-NFL-ROI-slope of MEM |  | 2.23E-15 (-1.54E-15, 5.58E-15) | 0.2978 | 0.5584 |  | 1.16E-15 (-3.13E-15, 5.54E-15) | 0.5919 | 0.8479 |  | 1.07E-15 (-2.10E-16, 3.26E-15) | 0.1340 | 0.2467 |  | 5.03E-16 (3.26E-17, 1.53E-15) | 0.0740 | 0.1025 |  | 1.28E-15 (2.53E-16, 3.46E-15) | **0.0220** | **0.0480** |  | -7.10E-16 (-2.29E-15, 3.74E-17) | 0.1200 | 0.3050 |  |
| HYP-NFL-ROI-slope of LAN |  | 2.04E-04 (-4.57E-04, 6.48E-04) | 0.5417 | 0.7655 |  | 2.84E-05 (-6.83E-04, 6.53E-04) | 0.9323 | 0.9645 |  | 1.76E-04 (-4.92E-05, 5.14E-04) | 0.1380 | 0.2467 |  | 8.62E-05 (8.57E-06, 2.62E-04) | 0.0660 | 0.1025 |  | 2.19E-04 (3.61E-05, 5.85E-04) | **0.0180** | **0.0480** |  | -1.30E-04 (-3.94E-04, -9.38E-07) | 0.0880 | 0.3050 |  |
| HYP-NFL-ROI-slope of EF |  | -4.39E-05 (-9.67E-04, 8.20E-04) | 0.9343 | 0.9475 |  | -2.17E-04 (-0.0014, 6.79E-04) | 0.6899 | 0.8479 |  | 1.74E-04 (-1.40E-04, 6.29E-04) | 0.3080 | 0.4617 |  | 9.99E-05 (5.22E-06, 3.60E-04) | 0.0760 | 0.1025 |  | 2.54E-04 (3.99E-05, 7.25E-04) | **0.0240** | **0.0480** |  | -1.80E-04 (-6.08E-044.80E-06) | 0.1220 | 0.3050 |  |
| PSY-NFL-ROI-slope of MMSE |  | 0.2177 (-0.2791, 0.6041) | 0.5620 | 0.7655 |  | 0.4654 (-0.0257, 1.1195) | 0.0901 | 0.7769 |  | -0.2523 (-0.7846, 0.3027) | 0.3540 | 0.4617 |  | -0.2131 (-0.6147, 0.0694) | 0.2400 | 0.2667 |  | -0.0200 (-0.3604, 0.4152) | 0.8840 | 0.9822 |  | -0.0192 (-0.2346, 0.0891) | 0.7400 | 0.9240 |  |
| PSY-NFL-ROI-slope of ADAS13 |  | -0.6565 (-1.3704, 0.5082) | 0.3312 | 0.5845 |  | -1.1380 (-2.3187, -0.1319) | **0.0132** | 0.3956 |  | 0.4904 (-0.5314, 1.5133) | 0.3480 | 0.4617 |  | 0.4094 (-0.1423, 1.1277) | 0.2400 | 0.2667 |  | 0.0383 (-0.7994, 0.6796) | 0.8840 | 0.9822 |  | 0.0427 (-0.0949, 0.4558) | 0.6720 | 0.9240 |  |
| PSY-NFL-ROI-slope of CDRSB |  | -0.0697 (-0.4020, 0.3239) | 0.7691 | 0.8874 |  | -0.2474 (-0.6996, 0.0505) | 0.1397 | 0.7769 |  | 0.1810 (-0.1810, 0.5444) | 0.3360 | 0.4617 |  | 0.1331 (-0.0457, 0.3766) | 0.2400 | 0.2667 |  | 0.0125 (-0.2543, 0.2177) | 0.8840 | 0.9822 |  | 0.0355 (-0.0227, 0.2158) | 0.4400 | 0.8250 |  |
| PSY-NFL-ROI-slope of MEM |  | -1.41E-15 (-9.80E-15, 6.57E-15) | 0.7107 | 0.8874 |  | -1.32E-15 (-9.48E-15, 7.44E-15) | 0.7199 | 0.8479 |  | -9.32E-17 (-2.74E-15, 1.55E-15) | 0.9700 | 0.9700 |  | 2.05E-16 (-7.87E-16, 1.82E-15) | 0.6720 | 0.6740 |  | -4.55E-17 (-2.35E-15, 1.56E-15) | 0.9960 | 1.0000 |  | -2.53E-16 (-2.58E-15, 8.94E-16) | 0.6840 | 0.9240 |  |
| PSY-NFL-ROI-slope of LAN |  | -9.36E-05 (-0.0011, 0.0010) | 0.8740 | 0.9365 |  | -7.38E-05 (-0.0011, 0.0011) | 0.8966 | 0.9606 |  | -2.07E-05 (-4.37E-04, 2.66E-04) | 0.9560 | 0.9700 |  | 3.39E-05 (-1.37E-04, 2.90E-04) | 0.6720 | 0.6740 |  | -7.53E-06 (-3.95E-04, 2.58E-04) | 1.0000 | 1.0000 |  | -4.71E-05 (-3.95E-041.83E-04) | 0.6840 | 0.9240 |  |
| PSY-NFL-ROI-slope of EF |  | 5.40E-04 (-0.0013, 0.0021) | 0.5658 | 0.7655 |  | 5.76E-04 (-0.0010, 0.0022) | 0.5338 | 0.8479 |  | -3.79E-05 (-7.08E-04, 2.83E-04) | 0.8940 | 0.9579 |  | 3.76E-05 (-1.28E-04, 4.18E-04) | 0.6740 | 0.6740 |  | -8.34E-06 (-4.79E-04, 2.77E-04) | 0.9860 | 1.0000 |  | -6.71E-05 (-7.13E-042.60E-04) | 0.6940 | 0.9240 |  |
| AFF-NFL-ROI-slope of MMSE |  | -0.9306 (-1.6808, -0.5750) | **0.0013** | **0.0108** |  | -0.3033 (-0.8282, 0.3236) | 0.1813 | 0.7769 |  | -0.6178 (-1.1132, -0.2455) | **<0.001** | **<0.001** |  | -0.1965 (-0.4089, -0.0660) | **0.0040** | **0.0200** |  | -0.4202 (-0.8577, -0.0661) | **0.0240** | **0.0480** |  | -0.0011 (-0.1358, 0.1404) | 0.9180 | 0.9240 |  |
| AFF-NFL-ROI-slope of ADAS13 |  | 1.6595 (0.9427, 3.0861) | **0.0014** | **0.0108** |  | 0.4400 (-0.5182, 1.3862) | 0.2505 | 0.8479 |  | 1.2009 (0.4638, 2.0210) | **<0.001** | **<0.001** |  | 0.3811 (0.1115, 0.7245) | **0.0040** | **0.0200** |  | 0.8151 (0.1490, 1.5975) | **0.0240** | **0.0480** |  | 0.0047 (-0.2060, 0.2467) | 0.9060 | 0.9240 |  |
| AFF-NFL-ROI-slope of CDRSB |  | 0.5319 (0.3031, 0.9528) | **0.0037** | **0.0160** |  | 0.1033 (-0.2109, 0.4272) | 0.4563 | 0.8479 |  | 0.4212 (0.1833, 0.7020) | **<0.001** | **<0.001** |  | 0.1250 (0.0371, 0.2517) | **0.0040** | **0.0200** |  | 0.2674 (0.0446, 0.5300) | **0.0240** | **0.0480** |  | 0.0288 (-0.0356, 0.1499) | 0.4380 | 0.8250 |  |
| AFF-NFL-ROI-slope of MEM |  | -1.86E-15 (-5.00E-15, 5.55E-15) | 0.4805 | 0.7588 |  | -3.87E-15 (-9.93E-15, 2.91E-15) | 0.1540 | 0.7769 |  | 2.01E-15 (-6.78E-17, 5.31E-15) | 0.1100 | 0.2357 |  | 9.99E-16 (2.91E-16, 2.45E-15) | **0.0060** | **0.0257** |  | 2.14E-15 (4.69E-16, 5.28E-15) | **0.0240** | **0.0480** |  | -1.13E-15 (-3.30E-15, 1.12E-16) | 0.0760 | 0.3050 |  |
| AFF-NFL-ROI-slope of LAN |  | 4.45E-04 (-2.31E-04, 0.0013) | 0.2768 | 0.5536 |  | 2.48E-04 (-7.13E-04, 0.0011) | 0.5550 | 0.8479 |  | 1.99E-04 (-1.25E-04, 7.01E-04) | 0.3260 | 0.4617 |  | 1.40E-04 (3.89E-05, 3.86E-04) | **0.0120** | **0.0360** |  | 3.00E-04 (5.51E-05, 7.99E-04) | **0.0300** | 0.0529 |  | -2.41E-04 (-5.99E-04, -3.02E-05) | **0.0240** | 0.3050 |  |
| AFF-NFL-ROI-slope of EF |  | 1.61E-04 (-6.35E-04, 0.0019) | 0.8056 | 0.8951 |  | -2.07E-05 (-0.0016, 0.0013) | 0.9760 | 0.9760 |  | 1.86E-04 (-2.88E-04, 8.73E-04) | 0.5580 | 0.6696 |  | 1.63E-04 (3.44E-05, 4.50E-04) | **0.0180** | **0.0450** |  | 3.50E-04 (3.91E-05, 0.0010) | **0.0360** | 0.0600 |  | -3.27E-04 (-9.01E-04, 9.12E-06) | 0.0720 | 0.3050 |  |
| APA-NFL-ROI-slope of MMSE |  | -0.6092 (-1.6260, 0.0031) | 0.0561 | 0.1530 |  | -0.1656 (-1.0320, 0.4500) | 0.4904 | 0.8479 |  | -0.4451 (-1.0707, 0.0424) | **0.0320** | 0.0800 |  | -0.2140 (-0.4780, -0.0623) | **<0.001** | **<0.001** |  | -0.2280 (-0.8029, 0.2826) | 0.2820 | 0.3850 |  | -0.0031 (-0.1284, 0.1573) | 0.9240 | 0.9240 |  |
| APA-NFL-ROI-slope of ADAS13 |  | 0.9960 (-0.1375, 2.5649) | 0.0836 | 0.1930 |  | 0.1348 (-0.7890, 1.2150) | 0.7393 | 0.8479 |  | 0.8642 (-0.0682, 2.0011) | **0.0260** | 0.0709 |  | 0.4133 (0.1270, 0.8542) | **<0.001** | **<0.001** |  | 0.4404 (-0.5220, 1.5720) | 0.2820 | 0.3850 |  | 0.0106 (-0.2219, 0.2332) | 0.8780 | 0.9240 |  |
| APA-NFL-ROI-slope of CDRSB |  | 0.4372 (0.0231, 0.9051) | **0.0296** | 0.0888 |  | 0.1360 (-0.1989, 0.5856) | 0.3518 | 0.8479 |  | 0.3023 (0.0135, 0.6924) | **0.0200** | 0.0600 |  | 0.1333 (0.0445, 0.2965) | **<0.001** | **<0.001** |  | 0.1420 (-0.1838, 0.4969) | 0.2820 | 0.3850 |  | 0.0271 (-0.0381, 0.1333) | 0.4720 | 0.8329 |  |
| APA-NFL-ROI-slope of MEM |  | 1.55E-15 (-3.30E-15, 6.03E-15) | 0.5869 | 0.7655 |  | 8.62E-16 (-3.95E-15, 4.79E-15) | 0.7631 | 0.8479 |  | 6.91E-16 (-1.96E-15, 3.52E-15) | 0.5180 | 0.6475 |  | 8.93E-16 (2.32E-16, 2.46E-15) | **0.0120** | **0.0360** |  | 1.01E-15 (-1.05E-15, 3.73E-15) | 0.3080 | 0.3850 |  | -1.21E-15 (-3.75E-15, -1.21E-16) | 0.0660 | 0.3050 |  |
| APA-NFL-ROI-slope of LAN |  | 3.89E-04 (-5.17E-04, 0.0012) | 0.3813 | 0.6355 |  | 3.08E-04 (-5.42E-04, 0.0011) | 0.4843 | 0.8479 |  | 8.03E-05 (-3.75E-04, 5.47E-04) | 0.6340 | 0.7315 |  | 1.45E-04 (4.24E-05, 4.15E-04) | **0.0120** | **0.0360** |  | 1.64E-04 (-1.67E-04, 6.49E-04) | 0.3080 | 0.3850 |  | -2.29E-04 (-6.60E-04, -5.06E-05) | **0.0240** | 0.3050 |  |
| APA-NFL-ROI-slope of EF |  | -2.21E-04 (-0.0018, 0.0013) | 0.7554 | 0.8874 |  | -2.83E-04 (-0.0021, 0.0014) | 0.6949 | 0.8479 |  | 6.11E-05 (-5.79E-04, 7.01E-04) | 0.7820 | 0.8689 |  | 1.69E-04 (2.62E-05, 5.48E-04) | **0.0160** | **0.0436** |  | 1.91E-04 (-1.93E-04, 8.65E-04) | 0.3080 | 0.3850 |  | -2.99E-04 (-9.59E-04, -3.68E-07) | 0.0660 | 0.3050 |  |

Mediation analysis based on structural equation modeling identified three potential mediation pathways: (1) *NPSs → plasma NFL→ AD-signature ROI → cognition*, (2) *NPSs→ AD-signature ROI → cognition*, and (3) *NPSs → plasma NFL → cognition*. **All analyses were adjusted for age, sex, education, and APOE ε4 status, with intracranial volume additionally included as a covariate in models involving brain structure measures.** Multiple testing significance was corrected using FDR. Significant at the level of *P* < 0.05 were shown in bold. NPSs neuropsychiatric symptoms; HYP hyperactivity; PSY psychosis; AFF affective; APA apathy; MMSE Mini-Mental State Examination; ADAS Alzheimer’s disease Assessment Scale; CDRSB Clinical Dementia Rating Sum of Boxes; MEM Memory function; LAN Language; EF Executive function; NFL neurofilament light chain; ROI region of interest; FDR false discovery rate; CI Confidence Interval.

**Table S14. Sequential mediation models linking NPSs to the slope of cognition in the ADNI male subsample.**

| Models |  | Total Effect | | |  | Direct Effect | | |  | Total Indirect Effect | | |  | Pathway 1 | | |  | Pathway 2 | | |  | Pathway 3 | | |  |
| --- | --- | --- | --- | --- | --- | --- | --- | --- | --- | --- | --- | --- | --- | --- | --- | --- | --- | --- | --- | --- | --- | --- | --- | --- | --- |
|  |  | β (95%CI) | P | P_FDR |  | β (95%CI) | P | P_FDR |  | β (95%CI) | P | P_FDR |  | β (95%CI) | P | P_FDR |  | β (95%CI) | P | P_FDR |  | β (95%CI) | P | P_FDR |  |
| NPSs-NFL-ROI-slope of MMSE |  | -0.6248 (-1.0610, -0.3907) | **1.56E-05** | **1.56E-04** |  | -0.3264 (-0.5751, -0.0391) | **0.0072** | 0.0635 |  | -0.3000 (-0.4969, -0.1328) | **<0.001** | **<0.001** |  | -0.0261 (-0.0799, 0.0178) | 0.2500 | 0.7091 |  | -0.2535 (-0.4219, -0.1109) | **<0.001** | **<0.001** |  | -0.0204 (-0.0845, 0.0088) | 0.2580 | 0.5368 |  |
| NPSs-NFL-ROI-slope of ADAS13 |  | 1.1335 (0.6957, 1.9829) | **4.23E-05** | **3.17E-04** |  | 0.5686 (0.0175, 1.1334) | **0.0153** | 0.0712 |  | 0.5673 (0.2672, 0.9670) | **<0.001** | **<0.001** |  | 0.0487 (-0.0315, 0.1746) | 0.2960 | 0.7400 |  | 0.4944 (0.2377, 0.8575) | **0.0020** | **0.0100** |  | 0.0241 (-0.0148, 0.1346) | 0.3960 | 0.5557 |  |
| NPSs-NFL-ROI-slope of CDRSB |  | 0.4478 (0.2970, 0.7361) | **2.35E-06** | **7.05E-05** |  | 0.2482 (0.0597, 0.4079) | **0.0014** | **0.0214** |  | 0.2008 (0.0849, 0.3243) | **<0.001** | **<0.001** |  | 0.0171 (-0.0118, 0.0561) | 0.2600 | 0.7091 |  | 0.1699 (0.0755, 0.2771) | **0.0020** | **0.0100** |  | 0.0139 (-0.0090, 0.0541) | 0.2700 | 0.5368 |  |
| NPSs-NFL-ROI-slope of MEM |  | -2.08E-16 (-2.37E-15, 2.51E-15) | 0.8773 | 0.9677 |  | -2.71E-16 (-2.83E-15, 2.37E-15) | 0.8457 | 0.9564 |  | 6.70E-17 (-7.08E-16,1.01E-15) | 0.8820 | 0.9760 |  | 2.31E-17 (-4.17E-17, 2.30E-16) | 0.6720 | 0.9764 |  | 2.12E-16 (-4.04E-16, 1.16E-15) | 0.5920 | 0.9245 |  | -1.68E-16 (-7.73E-16, 7.45E-17) | 0.3400 | 0.5368 |  |
| NPSs-NFL-ROI-slope of LAN |  | -1.12E-04 (-5.33E-04, 3.08E-04) | 0.6147 | 0.8938 |  | -7.73E-05 (-5.25E-04, 3.61E-04) | 0.7363 | 0.9564 |  | -3.40E-05 (-1.75E-04,9.75E-05) | 0.6220 | 0.8886 |  | -9.13E-08 (-1.86E-05, 1.93E-05) | 0.9980 | 0.9980 |  | -8.40E-07 (-1.04E-04, 1.23E-04) | 1.0020 | 1.0000 |  | -3.31E-05 (-1.38E-04, 1.50E-05) | 0.3140 | 0.5368 |  |
| NPSs-NFL-ROI-slope of EF |  | 1.53E-05 (-6.54E-04, 8.38E-04) | 0.9677 | 0.9677 |  | -2.06E-05 (-8.46E-04, 8.33E-04) | 0.9583 | 0.9948 |  | 3.63E-05 (-1.74E-04,3.16E-04) | 0.7700 | 0.9760 |  | 5.56E-06 (-1.42E-05, 7.46E-05) | 0.7540 | 0.9835 |  | 5.12E-05 (-1.49E-04, 3.23E-04) | 0.6540 | 0.9245 |  | -2.04E-05 (-2.06E-04, 3.50E-05) | 0.6020 | 0.6248 |  |
| HYP-NFL-ROI-slope of MMSE |  | -0.4112 (-0.7844, -0.2618) | **7.70E-04** | **0.0029** |  | -0.1504 (-0.3590, 0.0810) | 0.1417 | 0.3269 |  | -0.2647 (-0.4247, -0.1234) | **0.0020** | **0.0100** |  | -0.0279 (-0.0879, 0.0146) | 0.1940 | 0.7091 |  | -0.2161 (-0.3483, -0.1169) | **<0.001** | **<0.001** |  | -0.0206 (-0.0823, 0.0071) | 0.2060 | 0.5368 |  |
| HYP-NFL-ROI-slope of ADAS13 |  | 0.6681 (0.3525, 1.3255) | **0.0044** | **0.0119** |  | 0.1709 (-0.2842, 0.5804) | 0.3865 | 0.7247 |  | 0.5029 (0.2658, 0.8241) | **<0.001** | **<0.001** |  | 0.0528 (-0.0245, 0.1731) | 0.2240 | 0.7091 |  | 0.4259 (0.2225, 0.6870) | **<0.001** | **<0.001** |  | 0.0242 (-0.0117, 0.1284) | 0.3380 | 0.5368 |  |
| HYP-NFL-ROI-slope of CDRSB |  | 0.3138 (0.2010, 0.5522) | **9.08E-05** | **5.45E-04** |  | 0.1392 (-0.0044, 0.2792) | **0.0341** | 0.1278 |  | 0.1772 (0.0894, 0.2895) | **<0.001** | **<0.001** |  | 0.0185 (-0.0049, 0.0610) | 0.1560 | 0.6686 |  | 0.1444 (0.0746, 0.2307) | **<0.001** | **<0.001** |  | 0.0143 (-0.0023, 0.0606) | 0.1600 | 0.5368 |  |
| HYP-NFL-ROI-slope of MEM |  | 3.02E-16 (-1.74E-15, 2.48E-15) | 0.7870 | 0.9080 |  | 3.06E-16 (-1.90E-15, 2.68E-15) | 0.7913 | 0.9564 |  | -1.07E-17 (-7.24E-16, 6.82E-16) | 0.9760 | 0.9760 |  | 1.93E-17 (-4.52E-17, 1.92E-16) | 0.7080 | 0.9764 |  | 1.43E-16 (-4.01E-16, 8.42E-16) | 0.6420 | 0.9245 |  | -1.73E-16 (-7.80E-16, 5.19E-17) | 0.2980 | 0.5368 |  |
| HYP-NFL-ROI-slope of LAN |  | -6.87E-05 (-4.66E-04, 3.05E-04) | 0.7103 | 0.8938 |  | -3.34E-05 (-4.61E-04, 3.45E-04) | 0.8607 | 0.9564 |  | -3.69E-05 (-1.80E-04, 6.93E-05) | 0.5560 | 0.8340 |  | -3.44E-07 (-1.97E-05, 1.47E-05) | 0.9820 | 0.9980 |  | -2.55E-06 (-9.28E-05, 9.46E-05) | 0.9560 | 0.9890 |  | -3.40E-05 (-1.33E-04, 1.10E-05) | 0.2600 | 0.5368 |  |
| HYP-NFL-ROI-slope of EF |  | -1.84E-04 (-8.18E-04, 5.77E-04) | 0.5578 | 0.8938 |  | -2.26E-04 (-8.79E-04, 6.22E-04) | 0.4899 | 0.8645 |  | 4.07E-05 (-1.51E-04, 2.56E-04) | 0.6880 | 0.9382 |  | 7.34E-06 (-1.22E-05, 6.72E-05) | 0.6760 | 0.9764 |  | 5.43E-05 (-1.13E-04, 2.63E-04) | 0.5620 | 0.9245 |  | -2.09E-05 (-1.83E-04, 3.10E-05) | 0.5820 | 0.6248 |  |
| PSY-NFL-ROI-slope of MMSE |  | -0.4484 (-1.1951, -0.0889) | **0.0192** | **0.0412** |  | -0.2272 (-0.6853, 0.0981) | 0.1416 | 0.3269 |  | -0.2179 (-0.5222, 0.1024) | 0.1620 | 0.3471 |  | -0.0324 (-0.1321, 0.0377) | 0.3780 | 0.8723 |  | -0.1634 (-0.4115, 0.0675) | 0.1600 | 0.3429 |  | -0.0220 (-0.1255, 0.0164) | 0.3920 | 0.5557 |  |
| PSY-NFL-ROI-slope of ADAS13 |  | 0.9396 (0.2007, 1.9882) | **0.0113** | **0.0283** |  | 0.5612 (-0.0089, 1.2143) | 0.0629 | 0.1886 |  | 0.3734 (-0.2121, 0.9130) | 0.1980 | 0.3713 |  | 0.0581 (-0.0707, 0.2732) | 0.4820 | 0.9640 |  | 0.2909 (-0.1821, 0.7346) | 0.2340 | 0.4680 |  | 0.0245 (-0.0210, 0.1672) | 0.6040 | 0.6248 |  |
| PSY-NFL-ROI-slope of CDRSB |  | 0.3937 (0.0837, 0.7316) | **0.0017** | **0.0057** |  | 0.2461 (0.0510, 0.4368) | **0.0130** | 0.0712 |  | 0.1456 (-0.0776, 0.3236) | 0.1460 | 0.3369 |  | 0.0215 (-0.0233, 0.0852) | 0.4100 | 0.8786 |  | 0.1091 (-0.0545, 0.2550) | 0.1580 | 0.3429 |  | 0.0150 (-0.0130, 0.0849) | 0.4260 | 0.5557 |  |
| PSY-NFL-ROI-slope of MEM |  | 8.38E-16 (-2.67E-15, 4.36E-15) | 0.6254 | 0.8938 |  | 9.13E-16 (-2.99E-15, 4.55E-15) | 0.5983 | 0.9447 |  | -6.44E-17 (-8.25E-16, 6.75E-16) | 0.8620 | 0.9760 |  | 2.30E-17 (-5.69E-17, 3.07E-16) | 0.7160 | 0.9764 |  | 1.14E-16 (-2.85E-16, 9.72E-16) | 0.6600 | 0.9245 |  | -2.01E-16 (-1.20E-15, 1.47E-16) | 0.4520 | 0.5650 |  |
| PSY-NFL-ROI-slope of LAN |  | 1.07E-04 (-5.42E-04, 6.44E-04) | 0.7069 | 0.8938 |  | 1.56E-04 (-5.24E-04, 6.68E-04) | 0.5846 | 0.9447 |  | -4.68E-05 (-2.09E-04, 6.33E-05) | 0.5020 | 0.8340 |  | -1.23E-06 (-3.08E-05, 1.71E-05) | 0.9380 | 0.9980 |  | -6.10E-06 (-1.18E-04, 7.91E-05) | 0.8940 | 0.9643 |  | -3.95E-05 (-2.03E-04, 3.15E-05) | 0.4180 | 0.5557 |  |
| PSY-NFL-ROI-slope of EF |  | 9.41E-04 (1.47E-04, 0.0022) | 0.0503 | 0.1006 |  | 9.47E-04 (1.22E-04, 0.0020) | 0.0522 | 0.1740 |  | -4.92E-06 (-2.06E-04, 2.05E-04) | 0.9540 | 0.9760 |  | 3.56E-06 (-2.67E-05, 6.59E-05) | 0.8400 | 0.9980 |  | 1.76E-05 (-1.02E-04, 2.68E-04) | 0.8040 | 0.9643 |  | -2.61E-05 (-2.99E-04, 4.01E-05) | 0.6780 | 0.6780 |  |
| AFF-NFL-ROI-slope of MMSE |  | -0.4000 (-0.9330, -0.1356) | **0.0141** | **0.0324** |  | -0.1417 (-0.4369, 0.1899) | 0.2876 | 0.5752 |  | -0.2572 (-0.4977, -0.0190) | **0.0380** | 0.1036 |  | -0.0615 (-0.1706, -0.0073) | 0.0620 | 0.6200 |  | -0.1543 (-0.3358, 0.0434) | 0.1200 | 0.3273 |  | -0.0415 (-0.1367, -1.71E-04) | 0.0920 | 0.5368 |  |
| AFF-NFL-ROI-slope of ADAS13 |  | 0.5502 (-0.0049, 1.4715) | 0.0794 | 0.1490 |  | 0.0933 (-0.6020, 0.6848) | 0.7175 | 0.9564 |  | 0.4550 (0.0076, 0.9132) | 0.0520 | 0.1300 |  | 0.1167 (0.0214, 0.3197) | **0.0460** | 0.6200 |  | 0.2880 (-0.1260, 0.6380) | 0.1400 | 0.3429 |  | 0.0502 (-0.0124, 0.2083) | 0.2660 | 0.5368 |  |
| AFF-NFL-ROI-slope of CDRSB |  | 0.3071 (0.1145, 0.6463) | **0.0041** | **0.0119** |  | 0.1324 (-0.1023, 0.3475) | 0.1221 | 0.3269 |  | 0.1741 (0.0239, 0.3685) | 0.0320 | 0.0960 |  | 0.0418 (0.0052, 0.1140) | 0.0580 | 0.6200 |  | 0.1038 (-0.0305, 0.2353) | 0.1180 | 0.3273 |  | 0.0284 (5.61E-05, 0.0950) | 0.0860 | 0.5368 |  |
| AFF-NFL-ROI-slope of MEM |  | -5.75E-16 (-2.87E-15, 2.07E-15) | 0.7025 | 0.8938 |  | -3.42E-16 (-3.08E-15, 2.74E-15) | 0.8235 | 0.9564 |  | -2.17E-16 (-9.23E-16, 4.44E-16) | 0.5320 | 0.8340 |  | 4.93E-17 (-7.73E-17, 3.49E-16) | 0.6100 | 0.9764 |  | 9.93E-17 (-1.65E-16, 8.33E-16) | 0.6780 | 0.9245 |  | -3.65E-16 (-1.21E-15, 2.42E-17) | 0.1880 | 0.5368 |  |
| AFF-NFL-ROI-slope of LAN |  | -8.25E-05 (-5.42E-04, 3.70E-04) | 0.7406 | 0.8938 |  | -1.66E-06 (-4.62E-04, 5.02E-04) | 0.9948 | 0.9948 |  | -7.70E-05 (-2.26E-04, 1.51E-05) | 0.1780 | 0.3560 |  | -1.32E-06 (-3.06E-05, 3.29E-05) | 0.9040 | 0.9980 |  | -2.66E-06 (-8.46E-05, 5.99E-05) | 0.9000 | 0.9643 |  | -7.30E-05 (-2.24E-04, -1.51E-06) | 0.1080 | 0.5368 |  |
| AFF-NFL-ROI-slope of EF |  | -2.51E-05 (-6.92E-04, 7.55E-04) | 0.9528 | 0.9677 |  | -1.53E-05 (-7.68E-04, 8.22E-04) | 0.9719 | 0.9948 |  | -8.08E-06 (-1.94E-04, 1.80E-04) | 0.9280 | 0.9760 |  | 1.22E-05 (-2.89E-05, 1.16E-04) | 0.6680 | 0.9764 |  | 2.45E-05 (-5.62E-05, 2.56E-04) | 0.7640 | 0.9643 |  | -4.48E-05 (-3.09E-04, 7.40E-05) | 0.5660 | 0.6248 |  |
| APA-NFL-ROI-slope of MMSE |  | -0.6795 (-1.3132, -0.2494) | **3.89E-04** | **0.0017** |  | -0.3758 (-0.7874, 0.0240) | **0.0166** | 0.0712 |  | -0.3027 (-0.6071, -0.0272) | **0.0140** | 0.0525 |  | -0.0383 (-0.1224, 0.0159) | 0.1560 | 0.6686 |  | -0.2370 (-0.4953, -0.0349) | **0.0160** | 0.0600 |  | -0.0273 (-0.1072, 0.0088) | 0.1720 | 0.5368 |  |
| APA-NFL-ROI-slope of ADAS13 |  | 1.4664 (0.5181, 2.8023) | **1.12E-04** | **5.59E-04** |  | 0.8309 (0.0938, 1.6160) | **0.0085** | 0.0635 |  | 0.6377 (0.1357,1.2859) | **0.0060** | **0.0257** |  | 0.0912 (-0.0230, 0.2844) | 0.1180 | 0.6686 |  | 0.5062 (0.0838, 1.0340) | **0.0080** | **0.0343** |  | 0.0403 (-0.0160, 0.2036) | 0.3140 | 0.5368 |  |
| APA-NFL-ROI-slope of CDRSB |  | 0.5571 (0.2679, 0.9425) | **8.48E-06** | **1.27E-04** |  | 0.3540 (0.1071, 0.6050) | **0.0004** | **0.0123** |  | 0.2025 (0.0381,0.4143) | **0.0180** | 0.0600 |  | 0.0256 (-0.0096, 0.0843) | 0.1500 | 0.6686 |  | 0.1580 (0.0214, 0.3174) | **0.0220** | 0.0733 |  | 0.0189 (-0.0064, 0.0689) | 0.1640 | 0.5368 |  |
| APA-NFL-ROI-slope of MEM |  | 5.64E-16 (-2.21E-15, 4.04E-15) | 0.7449 | 0.8938 |  | 6.16E-16 (-2.41E-15, 4.16E-15) | 0.7281 | 0.9564 |  | -4.89E-17 (-1.02E-15, 8.20E-16) | 0.9020 | 0.9760 |  | 2.81E-17 (-7.22E-17, 2.72E-16) | 0.7080 | 0.9764 |  | 1.72E-16 (-5.30E-16, 1.11E-15) | 0.6560 | 0.9245 |  | -2.49E-16 (-1.10E-15, 7.82E-17) | 0.2520 | 0.5368 |  |
| APA-NFL-ROI-slope of LAN |  | 1.73E-05 (-5.53E-04, 5.53E-04) | 0.9518 | 0.9677 |  | 7.62E-05 (-4.56E-04, 5.85E-04) | 0.7940 | 0.9564 |  | -5.83E-05 (-2.51E-04, 6.98E-05) | 0.3980 | 0.7024 |  | -1.33E-06 (-2.96E-05, 2.09E-05) | 0.9160 | 0.9980 |  | -8.14E-06 (-1.39E-04, 1.23E-04) | 0.8560 | 0.9643 |  | -4.88E-05 (-1.90E-04, 1.37E-05) | 0.1980 | 0.5368 |  |
| APA-NFL-ROI-slope of EF |  | 6.19E-04 (-4.98E-04, 0.0019) | 0.2036 | 0.3592 |  | 6.19E-04 (-4.50E-04, 0.0019) | 0.2159 | 0.4626 |  | 8.38E-07 (-2.56E-04, 3.17E-04) | 0.9520 | 0.9760 |  | 4.47E-06 (-3.19E-05, 8.20E-05) | 0.8760 | 0.9980 |  | 2.74E-05 (-1.76E-04, 3.52E-04) | 0.8240 | 0.9643 |  | -3.10E-05 (-2.81E-04, 5.68E-05) | 0.5980 | 0.6248 |  |

Mediation analysis based on structural equation modeling identified three potential mediation pathways: (1) *NPSs → plasma NFL→ AD-signature ROI → cognition*, (2) *NPSs→ AD-signature ROI → cognition*, and (3) *NPSs → plasma NFL → cognition*. **All analyses were adjusted for age, sex, education, and APOE ε4 status, with intracranial volume additionally included as a covariate in models involving brain structure measures.** Multiple testing significance was corrected using FDR. Significant at the level of *P* < 0.05 were shown in bold. NPSs neuropsychiatric symptoms; HYP hyperactivity; PSY psychosis; AFF affective; APA apathy; MMSE Mini-Mental State Examination; ADAS Alzheimer’s disease Assessment Scale; CDRSB Clinical Dementia Rating Sum of Boxes; MEM Memory function; LAN Language; EF Executive function; NFL neurofilament light chain; ROI region of interest; FDR false discovery rate; CI, Confidence Interval.

**Table S15. Discriminative performance of models for concurrent cognitive status.**

| Models |  | Ten-fold CV | | | | | | | | |  | Apparent | | | | | | | | | | | |
| --- | --- | --- | --- | --- | --- | --- | --- | --- | --- | --- | --- | --- | --- | --- | --- | --- | --- | --- | --- | --- | --- | --- | --- |
|  |  | AUC (95%CI) |  | Sensitivity |  | Specificity |  | T value |  | P Value |  | AUC (95%CI) |  | Sensitivity |  | Specificity |  | Z statistic |  | P Value |  | Optimism |  |
| REF (age, sex, education, *APOE* ε4) |  | 0.6754 (0.6389, 0.7120) |  | 0.5855 |  | 0.7516 |  | 9.4188 |  | **< 0.001** |  | 0.6796 (0.6528, 0.7065) |  | 0.5829 |  | 0.71510 |  | 13.1130 |  | **< 0.001** |  | 0.0042 |  |
| REF + Hyperactivity |  | 0.7377 (0.7175, 0.7578) |  | 0.6661 |  | 0.7446 |  | 23.1129 |  | **< 0.001** |  | 0.7383 (0.7137, 0.7629) |  | 0.6852 |  | 0.6913 |  | 18.9824 |  | **< 0.001** |  | 0.0006 |  |
| REF + Psychosis |  | 0.6913 (0.6729, 0.7096) |  | 0.6365 |  | 0.7223 |  | 20.4437 |  | **< 0.001** |  | 0.6915 (0.6650, 0.7179) |  | 0.6285 |  | 0.6866 |  | 14.1972 |  | **< 0.001** |  | 0.0002 |  |
| REF + Affective |  | 0.7278 (0.7058, 0.7498) |  | 0.6561 |  | 0.7444 |  | 20.3164 |  | **< 0.001** |  | 0.7281 (0.7030, 0.7532) |  | 0.5907 |  | 0.7780 |  | 17.8242 |  | **< 0.001** |  | 0.0003 |  |
| REF + Apathy |  | 0.7136 (0.6761, 0.7512) |  | 0.6237 |  | 0.7503 |  | 11.1589 |  | **< 0.001** |  | 0.7182 (0.6929, 0.7435) |  | 0.6407 |  | 0.7102 |  | 16.8978 |  | **< 0.001** |  | 0.0046 |  |
| REF + NPI-Q Score |  | 0.7580 (0.7397, 0.7763) |  | 0.6620 |  | 0.7944 |  | 27.6345 |  | **< 0.001** |  | 0.7576 (0.7337, 0.7815) |  | 0.6850 |  | 0.7354 |  | 13.1130 |  | **< 0.001** |  | -0.0004 |  |
| REF + Plasma NFL |  | 0.7648 (0.7339, 0.7957) |  | 0.7367 |  | 0.7140 |  | 16.8076 |  | **< 0.001** |  | 0.7657 (0.7419, 0.7894) |  | 0.7453 |  | 0.6693 |  | 21.9210 |  | **< 0.001** |  | 0.0008 |  |
| REF + NPI-Q Score + Plasma NFL |  | 0.8210 (0.8025, 0.8395) |  | 0.7624 |  | 0.7609 |  | 33.9980 |  | **< 0.001** |  | 0.8228 (0.8023, 0.8433) |  | 0.7575 |  | 0.7354 |  | 30.7998 |  | **< 0.001** |  | 0.0018 |  |

REF reference; AUC, area under the curve; CI Confidence Interval; CV, cross‑validated; *APOE* ε4 apolipoprotein ε4; NPI-Q Neuropsychiatric Inventory Questionnaire. Optimism = apparent AUC − CV-AUC. One‑sample t‑test compared mean CV‑AUC against 0.5. DeLong’s test compared apparent AUC against 0.5. Youden index determined optimal sensitivity and specificity

**Table S16. Time-dependent Cox proportional hazards model for NPSs subsyndrome onset.**

| **Variable** |  | **HR (95%CI)** |  | **P value** |  |
| --- | --- | --- | --- | --- | --- |
| **Subsyndrome (REF: Affective)** |  |  |  |  |  |
| Hyperactivity |  | 0.99 (0.83-1.19) |  | 0.935 |  |
| Apathy |  | 0.65 (0.53-0.79) |  | **< 0.001** |  |
| Psychosis |  | 0.66 (0.54-0.80) |  | **< 0.001** |  |
| **Fixed effects** |  |  |  |  |  |
| Age (per year) |  | 1.03 (1.00-1.05) |  | **0.018** |  |
| Sex (male vs. female) |  | 0.67 (0.49-0.90) |  | **0.008** |  |
| Education (per year) |  | 0.93 (0.88-0.98) |  | **0.007** |  |
| *APOE* ε4 (carrier vs. non‑carrier) |  | 1.60 (1.20-2.14) |  | **0.002** |  |
| **Time‑varying effects** |  |  |  |  |  |
| tt(Sex) |  | 1.19 (0.96-1.47) |  | 0.116 |  |
| tt(*APOE* ε4) |  | 0.96 (0.78-1.18) |  | 0.679 |  |
| tt(Age) |  | 1.00 (0.98-1.02) |  | 0.982 |  |
| tt(Education) |  | 1.02 (0.99-1.06) |  | 0.202 |  |

Time-varying effects were modeled as covariate × log(t + 1). Non-significant time-varying effects (all P > 0.05) indicate that the proportional hazards assumption held for all covariates. NPSs neuropsychiatric symptoms; HR, hazard ratio; CI, confidence interval; REF reference category; *APOE* ε4 apolipoprotein ε4.

**Table S17. Sequential mediation models for the reverse direction (atrophy → NPSs → NFL → cognition) in the ADNI cohort.**

| Models | Total Effect | | |  | Direct Effect | | |  | Total Indirect Effect | | |  | Pathway 1 | | |  | Pathway 2 | | |  | Pathway 3 | | |  |
| --- | --- | --- | --- | --- | --- | --- | --- | --- | --- | --- | --- | --- | --- | --- | --- | --- | --- | --- | --- | --- | --- | --- | --- | --- |
|  | β | P | P_FDR |  | β | P | P_FDR |  | β | P | P_FDR |  | β | P | P_FDR |  | β | P | P_FDR |  | β | P | P_FDR |  |
| ROI-NPSs-NFL-MMSE | -0.5484 | **2.7781E-26** | **4.8951E-26** |  | -0.6383 | **2.5185E-26** | **4.1967E-26** |  | -0.0704 | **0.010** | **0.015** |  | -2.8006E-04 | 0.8500 | 0.9999 |  | -0.0234 | 0.2600 | 0.3391 |  | -0.0468 | **0.0040** | **0.0092** |  |
| ROI-NPSs-NFL-ADAS13 | 0.6114 | **1.4277E-33** | **8.5660E-33** |  | 0.6809 | **8.1482E-34** | **4.8889E-33** |  | 0.1183 | **<0.001** | **<0.001** |  | 6.9789E-04 | 0.7600 | 0.9999 |  | 0.0569 | **<0.001** | **<0.001** |  | 0.0607 | **<0.001** | **<0.001** |  |
| ROI-NPSs-NFL-CDRSB | 0.5657 | **3.2929E-28** | **9.8787E-28** |  | 0.6195 | **2.7156E-25** | **4.0734E-25** |  | 0.1163 | **<0.001** | **<0.001** |  | 5.4477E-04 | 0.7980 | 0.9999 |  | 0.0455 | **0.0280** | **0.0467** |  | 0.0702 | **<0.001** | **<0.001** |  |
| ROI-NPSs-NFL-MEM | -0.5477 | **3.2634E-26** | **4.8951E-26** |  | -0.6082 | **5.8484E-28** | **1.3496E-27** |  | -0.1287 | **<0.001** | **<0.001** |  | -7.3737E-04 | 0.7900 | 0.9999 |  | -0.0616 | **<0.001** | **<0.001** |  | -0.0664 | **<0.001** | **<0.001** |  |
| ROI-NPSs-NFL-LAN | -0.4833 | **1.3699E-18** | **1.3699E-18** |  | -0.5217 | **7.6337E-17** | **8.4819E-17** |  | -0.0872 | **0.048** | 0.0576 |  | -5.9909E-04 | 0.8100 | 0.9999 |  | -0.0500 | 0.5720 | 0.6129 |  | -0.0366 | **0.0120** | **0.0257** |  |
| ROI-NPSs-NFL-EF | -0.4672 | **5.8588E-20** | **7.0305E-20** |  | -0.5465 | **1.3631E-15** | **1.3631E-15** |  | -0.0551 | **0.002** | **0.0035** |  | -1.5440E-04 | 0.7920 | 0.9999 |  | -0.0129 | **0.0180** | **0.0415** |  | -0.0421 | **0.0320** | 0.0533 |  |
| ROI-HYP-NFL-MMSE | -0.5484 | **2.7781E-26** | **4.8951E-26** |  | -0.6574 | **1.5571E-27** | **2.9195E-27** |  | -0.0513 | **0.042** | 0.0548 |  | 1.6386E-05 | 0.9999 | 0.9999 |  | -0.0248 | 0.2320 | 0.3314 |  | -0.0265 | **0.0380** | 0.0600 |  |
| ROI-HYP-NFL-ADAS13 | 0.6114 | **1.4277E-33** | **8.5660E-33** |  | 0.6998 | **5.7909E-35** | **4.3432E-34** |  | 0.0993 | **<0.001** | **<0.001** |  | -5.2800E-05 | 0.9999 | 0.9999 |  | 0.0592 | **<0.001** | **<0.001** |  | 0.0402 | **<0.001** | **<0.001** |  |
| ROI-HYP-NFL-CDRSB | 0.5657 | **3.2929E-28** | **9.8787E-28** |  | 0.6355 | **2.6579E-26** | **4.1967E-26** |  | 0.1003 | **<0.001** | **<0.001** |  | -3.1554E-05 | 0.9999 | 0.9999 |  | 0.0478 | **0.0200** | **0.0429** |  | 0.0525 | **0.0020** | **0.0055** |  |
| ROI-HYP-NFL-MEM | -0.5477 | **3.2634E-26** | **4.8951E-26** |  | -0.6278 | **4.2465E-29** | **1.1581E-28** |  | -0.1092 | **<0.001** | **<0.001** |  | 4.2235E-05 | 0.9999 | 0.9999 |  | -0.0640 | **<0.001** | **<0.001** |  | -0.0452 | **<0.001** | **<0.001** |  |
| ROI-HYP-NFL-LAN | -0.4833 | **1.3699E-18** | **1.3699E-18** |  | -0.5303 | **6.5948E-18** | **7.9137E-18** |  | -0.0786 | 0.192 | 0.2079 |  | 3.4034E-05 | 0.9220 | 0.9999 |  | -0.0516 | 0.5480 | 0.6129 |  | -0.0271 | 0.1160 | 0.1513 |  |
| ROI-HYP-NFL-EF | -0.4672 | **5.8588E-20** | **7.0305E-20** |  | -0.5667 | **3.5860E-16** | **3.8422E-16** |  | -0.0349 | **<0.001** | **<0.001** |  | 9.2932E-06 | 0.9999 | 0.9999 |  | -0.0141 | **0.0160** | **0.0415** |  | -0.0208 | 0.0520 | 0.0780 |  |
| ROI-PSY-NFL-MMSE | -0.5484 | **2.7781E-26** | **4.8951E-26** |  | -0.6764 | **1.0798E-29** | **4.0493E-29** |  | -0.0323 | 0.148 | 0.1708 |  | 8.5830E-05 | 0.8580 | 0.9999 |  | -0.0253 | 0.2500 | 0.3391 |  | -0.0071 | 0.2540 | 0.2931 |  |
| ROI-PSY-NFL-ADAS13 | 0.6114 | **1.4277E-33** | **8.5660E-33** |  | 0.7341 | **6.2642E-38** | **1.8792E-36** |  | 0.0650 | **<0.001** | **<0.001** |  | -2.0702E-04 | 0.8320 | 0.9999 |  | 0.0596 | **<0.001** | **<0.001** |  | 0.0056 | 0.3060 | 0.3279 |  |
| ROI-PSY-NFL-CDRSB | 0.5657 | **3.2929E-28** | **9.8787E-28** |  | 0.6746 | **3.3243E-29** | **9.9730E-29** |  | 0.0612 | **0.006** | **0.010** |  | -1.6509E-04 | 0.8300 | 0.9999 |  | 0.0486 | **0.0180** | **0.0415** |  | 0.0127 | 0.1420 | 0.1704 |  |
| ROI-PSY-NFL-MEM | -0.5477 | **3.2634E-26** | **4.8951E-26** |  | -0.6672 | **4.2513E-32** | **2.1256E-31** |  | -0.0697 | **<0.001** | **<0.001** |  | 2.1871E-04 | 0.8280 | 0.9999 |  | -0.0644 | **<0.001** | **<0.001** |  | -0.0055 | 0.2860 | 0.3178 |  |
| ROI-PSY-NFL-LAN | -0.4833 | **1.3699E-18** | **1.3699E-18** |  | -0.5549 | **1.1643E-19** | **1.6633E-19** |  | -0.054 | 0.574 | 0.574 |  | 1.7586E-04 | 0.8820 | 0.9999 |  | -0.0518 | 0.5540 | 0.6129 |  | -0.0024 | 0.9180 | 0.918 |  |
| ROI-PSY-NFL-EF | -0.4672 | **5.8588E-20** | **7.0305E-20** |  | -0.5883 | **6.1585E-18** | **7.6981E-18** |  | -0.0133 | **0.012** | **0.0171** |  | 4.7667E-05 | 0.8100 | 0.9999 |  | -0.0140 | **0.0220** | **0.0440** |  | 6.5540E-04 | 0.6380 | 0.6600 |  |
| ROI-AFF-NFL-MMSE | -0.5484 | **2.7781E-26** | **4.8951E-26** |  | -0.6577 | **2.1118E-28** | **5.2795E-28** |  | -0.0510 | **0.038** | 0.0518 |  | -7.9937E-04 | 0.4640 | 0.9999 |  | -0.0194 | 0.3580 | 0.4296 |  | -0.0307 | **0.0040** | **0.0092** |  |
| ROI-AFF-NFL-ADAS13 | 0.6114 | **1.4277E-33** | **8.5660E-33** |  | 0.7111 | **1.5645E-36** | **2.3468E-35** |  | 0.0881 | **<0.001** | **<0.001** |  | 0.0021 | 0.1600 | 0.9086 |  | 0.0519 | **<0.001** | **<0.001** |  | 0.0341 | **<0.001** | **<0.001** |  |
| ROI-AFF-NFL-CDRSB | 0.5657 | **3.2929E-28** | **9.8787E-28** |  | 0.6530 | **7.4830E-28** | **1.6035E-27** |  | 0.0828 | **<0.001** | **<0.001** |  | 0.0016 | 0.2040 | 0.9086 |  | 0.0399 | **0.0420** | 0.0663 |  | 0.0412 | **<0.001** | **<0.001** |  |
| ROI-AFF-NFL-MEM | -0.5477 | **3.2634E-26** | **4.8951E-26** |  | -0.6376 | **1.2741E-30** | **5.4605E-30** |  | -0.0993 | **<0.001** | **<0.001** |  | -0.0023 | 0.1700 | 0.9086 |  | -0.0554 | **<0.001** | **<0.001** |  | -0.0416 | **<0.001** | **<0.001** |  |
| ROI-AFF-NFL-LAN | -0.4833 | **1.3699E-18** | **1.3699E-18** |  | -0.5386 | **1.5642E-18** | **2.1330E-18** |  | -0.0703 | 0.216 | 0.2234 |  | -0.0019 | 0.6880 | 0.9999 |  | -0.0463 | 0.6060 | 0.6269 |  | -0.0221 | 0.0940 | 0.1282 |  |
| ROI-AFF-NFL-EF | -0.4672 | **5.8588E-20** | **7.0305E-20** |  | -0.5706 | **6.1143E-17** | **7.0549E-17** |  | -0.0310 | **0.010** | **0.015** |  | -4.3830E-04 | 0.1960 | 0.9086 |  | -0.0106 | **0.0260** | **0.0459** |  | -0.0199 | **0.0160** | **0.0300** |  |
| ROI-APA-NFL-MMSE | -0.5484 | **2.7781E-26** | **4.8951E-26** |  | -0.6550 | **1.1916E-27** | **2.3832E-27** |  | -0.0537 | **0.046** | 0.0575 |  | -0.001 | 0.4980 | 0.9990 |  | -0.0199 | 0.3460 | 0.4296 |  | -0.0327 | 0.0700 | 0.1000 |  |
| ROI-APA-NFL-ADAS13 | 0.6114 | **1.4277E-33** | **8.5660E-33** |  | 0.7057 | **2.8840E-35** | **2.8840E-34** |  | 0.0935 | **<0.001** | **<0.001** |  | 0.0027 | 0.1620 | 0.9086 |  | 0.0518 | **<0.001** | **<0.001** |  | 0.0389 | **<0.001** | **<0.001** |  |
| ROI-APA-NFL-CDRSB | 0.5657 | **3.2929E-28** | **9.8787E-28** |  | 0.6458 | **7.5923E-27** | **1.3398E-26** |  | 0.0900 | **0.002** | **0.0035** |  | 0.0020 | 0.2460 | 0.9225 |  | 0.0400 | 0.0540 | 0.0810 |  | 0.0479 | **0.0220** | **0.0388** |  |
| ROI-APA-NFL-MEM | -0.5477 | **3.2634E-26** | **4.8951E-26** |  | -0.6309 | **1.7012E-29** | **5.6708E-29** |  | -0.1061 | **<0.001** | **<0.001** |  | -0.0028 | 0.1920 | 0.9086 |  | -0.0555 | **<0.001** | **<0.001** |  | -0.0478 | **<0.001** | **<0.001** |  |
| ROI-APA-NFL-LAN | -0.4833 | **1.3699E-18** | **1.3699E-18** |  | -0.5245 | **6.1247E-18** | **7.6981E-18** |  | -0.0844 | 0.1940 | 0.2079 |  | -0.0023 | 0.6660 | 0.9999 |  | -0.0448 | 0.6380 | 0.6380 |  | -0.0373 | 0.1360 | 0.1700 |  |
| ROI-APA-NFL-EF | -0.4672 | **5.8588E-20** | **7.0305E-20** |  | -0.5644 | **4.3499E-16** | **4.4999E-16** |  | -0.0372 | **<0.001** | **<0.001** |  | -5.2857E-04 | 0.2120 | 0.9086 |  | -0.0104 | **0.0240** | **0.0450** |  | -0.0262 | **0.0140** | **0.0280** |  |

Mediation analysis based on structural equation modeling identified three potential mediation pathways: (1) *AD-signature ROI → NPSs → plasma NFL→ cognition*, (2) *AD-signature ROI →plasma NFL→ cognition*, and (3) *AD-signature ROI → NPSs → cognition*. **All analyses were adjusted for age, sex, education, and APOE ε4 status, with intracranial volume additionally included as a covariate in models involving brain structure measures.** Multiple testing significance was corrected using FDR. Significant at the level of *P* < 0.05 were shown in bold. NPSs neuropsychiatric symptoms; HYP hyperactivity; PSY psychosis; AFF affective; APA apathy; MMSE Mini-Mental State Examination; ADAS Alzheimer’s disease Assessment Scale; CDRSB Clinical Dementia Rating Sum of Boxes; MEM Memory function; LAN Language; EF Executive function; NFL neurofilament light chain; ROI region of interest; FDR false discovery rate; CI Confidence Interval.

**Table S18. Sequential mediation models for the reverse direction (*ROI atrophy → NPSs → NFL → slope of cognition*) in the ADNI cohort.**

| Models | Total Effect | | |  | Direct Effect | | |  | Total Indirect Effect | | |  | Pathway 1 | | |  | Pathway 2 | | |  | Pathway 3 | | |  |
| --- | --- | --- | --- | --- | --- | --- | --- | --- | --- | --- | --- | --- | --- | --- | --- | --- | --- | --- | --- | --- | --- | --- | --- | --- |
|  | β | P | P_FDR |  | β | P | P_FDR |  | β | P | P_FDR |  | β | P | P_FDR |  | β | P | P_FDR |  | β | P | P_FDR |  |
| ROI-NPSs-NFL-slope of MMSE | -0.5918 | **1.2308E-29** | **2.4617E-29** |  | -0.6651 | **4.4735E-25** | **8.9470E-25** |  | -0.0878 | 0.008 | 0.0525 |  | 2.3559E-04 | 0.8540 | 0.9956 |  | -0.0420 | 0.0820 | 0.1500 |  | -0.0460 | **0.010** | 0.1000 |  |
| ROI-NPSs-NFL-slope of ADAS13 | 0.5991 | **2.5312E-30** | **7.5937E-30** |  | 0.6962 | **9.4707E-28** | **3.0234E-27** |  | 0.0629 | 0.046 | 0.0812 |  | -1.4799E-04 | 0.8700 | 0.9956 |  | 0.0299 | 0.1940 | 0.2143 |  | 0.0332 | 0.0700 | 0.3500 |  |
| ROI-NPSs-NFL-slope of CDRSB | 0.6022 | **5.4196E-31** | **3.2517E-30** |  | 0.6691 | **7.5379E-27** | **1.7395E-26** |  | 0.1026 | **0.002** | **0.0400** |  | -4.0132E-04 | 0.8120 | 0.9956 |  | 0.0569 | **0.0180** | 0.0600 |  | 0.0461 | **0.0040** | 0.0900 |  |
| ROI-NPSs-NFL-slope of MEM | 0.1405 | **0.0157** | **0.0236** |  | 0.2072 | **0.0110** | **0.0173** |  | -0.0638 | 0.064 | 0.0960 |  | 2.6719E-04 | 0.8840 | 0.9956 |  | -0.0641 | **0.0220** | 0.0600 |  | 1.5707E-05 | 0.9240 | 0.9999 |  |
| ROI-NPSs-NFL-slope of LAN | 0.0987 | 0.0907 | 0.0983 |  | 0.1660 | **0.0403** | **0.0465** |  | -0.0717 | **0.032** | 0.0713 |  | 3.1735E-04 | 0.8960 | 0.9956 |  | -0.0761 | **0.0100** | 0.0600 |  | 0.0041 | 0.8500 | 0.9999 |  |
| ROI-NPSs-NFL-slope of EF | 0.0964 | 0.0983 | 0.0983 |  | 0.1648 | **0.0437** | **0.0485** |  | -0.0579 | 0.158 | 0.1823 |  | 1.9146E-04 | 0.9360 | 0.9999 |  | -0.0459 | 0.1780 | 0.2143 |  | -0.0121 | 0.5560 | 0.9267 |  |
| ROI-HYP-NFL-slope of MMSE | -0.5918 | **1.2308E-29** | **2.4617E-29** |  | -0.6916 | **1.4767E-26** | **3.1643E-26** |  | -0.0613 | **0.038** | 0.0713 |  | 6.54857E-05 | 0.9990 | 0.9999 |  | -0.0414 | 0.0900 | 0.1500 |  | -0.0200 | 0.1880 | 0.5127 |  |
| ROI-HYP-NFL-slope of ADAS13 | 0.5991 | **2.5312E-30** | **7.5937E-30** |  | 0.7178 | **3.6048E-29** | **1.5449E-28** |  | 0.0413 | 0.12 | 0.1565 |  | -3.54253E-05 | 0.9990 | 0.9999 |  | 0.0294 | 0.2000 | 0.2143 |  | 0.0119 | 0.3620 | 0.7688 |  |
| ROI-HYP-NFL-slope of CDRSB | 0.6022 | **5.4196E-31** | **3.2517E-30** |  | 0.6797 | **1.0078E-27** | **3.0234E-27** |  | 0.092 | **0.004** | **0.0400** |  | -1.3753E-04 | 0.8840 | 0.9956 |  | 0.0561 | **0.0200** | 0.0600 |  | 0.0360 | **0.0140** | 0.1050 |  |
| ROI-HYP-NFL-slope of MEM | 0.1405 | **0.0157** | **0.0236** |  | 0.2007 | **0.0130** | **0.0196** |  | -0.0572 | 0.080 | 0.1143 |  | -4.9207E-04 | 0.8680 | 0.9956 |  | -0.0634 | **0.0220** | 0.0600 |  | 0.0067 | 0.7620 | 0.9999 |  |
| ROI-HYP-NFL-slope of LAN | 0.0987 | 0.0907 | 0.0983 |  | 0.1754 | **0.0291** | **0.0416** |  | -0.0812 | **0.014** | 0.0525 |  | -5.8259E-04 | 0.8580 | 0.9956 |  | -0.0751 | **0.0080** | 0.0600 |  | -0.0055 | 0.7760 | 0.9999 |  |
| ROI-HYP-NFL-slope of EF | 0.0964 | 0.0983 | 0.0983 |  | 0.1702 | **0.0357** | **0.0460** |  | -0.0633 | 0.112 | 0.1527 |  | -3.4803E-04 | 0.8520 | 0.9956 |  | -0.0449 | 0.1820 | 0.2143 |  | -0.0181 | 0.4100 | 0.7688 |  |
| ROI-PSY-NFL-slope of MMSE | -0.5918 | **1.2308E-29** | **2.4617E-29** |  | -0.7112 | **9.0181E-29** | **3.3818E-28** |  | -0.0417 | 0.132 | 0.1584 |  | -6.6280E-04 | 0.3880 | 0.9814 |  | -0.0404 | 0.0880 | 0.1500 |  | -6.7850E-04 | 0.9640 | 0.9999 |  |
| ROI-PSY-NFL-slope of ADAS13 | 0.5991 | **2.5312E-30** | **7.5937E-30** |  | 0.7293 | **4.2250E-31** | **1.2675E-29** |  | 0.0298 | 0.216 | 0.2379 |  | 4.5260E-04 | 0.5240 | 0.9956 |  | 0.0287 | 0.2000 | 0.2143 |  | 6.1128E-04 | 0.9200 | 0.9999 |  |
| ROI-PSY-NFL-slope of CDRSB | 0.6022 | **5.4196E-31** | **3.2517E-30** |  | 0.7112 | **1.6185E-30** | **2.4278E-29** |  | 0.0604 | **0.020** | 0.0600 |  | 8.5072E-04 | 0.3980 | 0.9814 |  | 0.0533 | **0.0220** | 0.0600 |  | 0.0063 | 0.3960 | 0.7688 |  |
| ROI-PSY-NFL-slope of MEM | 0.1405 | **0.0157** | **0.0236** |  | 0.2068 | **0.0087** | **0.0154** |  | -0.0634 | **0.034** | 0.0713 |  | -3.7135E-04 | 0.7320 | 0.9956 |  | -0.0635 | **0.0240** | 0.0600 |  | 4.5203E-04 | 0.9999 | 0.9999 |  |
| ROI-PSY-NFL-slope of LAN | 0.0987 | 0.0907 | 0.0983 |  | 0.1688 | **0.0313** | **0.0427** |  | -0.0745 | **0.016** | 0.0533 |  | -4.4201E-04 | 0.7220 | 0.9956 |  | -0.0756 | **0.0100** | 0.0600 |  | 0.0015 | 0.8480 | 0.9999 |  |
| ROI-PSY-NFL-slope of EF | 0.0964 | 0.0983 | 0.0983 |  | 0.1409 | **0.0727** | **0.0727** |  | -0.034 | 0.362 | 0.3620 |  | -2.7485E-04 | 0.7480 | 0.9956 |  | -0.047 | 0.1720 | 0.2143 |  | 0.0132 | 0.1840 | 0.5127 |  |
| ROI-AFF-NFL-slope of MMSE | -0.5918 | **1.2308E-29** | **2.4617E-29** |  | -0.6945 | **2.0273E-27** | **5.0682E-27** |  | -0.0584 | 0.050 | 0.0833 |  | -0.0019 | 0.1960 | 0.9814 |  | -0.0354 | 0.1240 | 0.1860 |  | -0.0211 | 0.1520 | 0.5067 |  |
| ROI-AFF-NFL-slope of ADAS13 | 0.5991 | **2.5312E-30** | **7.5937E-30** |  | 0.7197 | **5.2399E-30** | **5.2399E-29** |  | 0.0394 | 0.126 | 0.1575 |  | 0.0014 | 0.2960 | 0.9814 |  | 0.0256 | 0.2640 | 0.2640 |  | 0.0124 | 0.3400 | 0.7688 |  |
| ROI-AFF-NFL-slope of CDRSB | 0.6022 | **5.4196E-31** | **3.2517E-30** |  | 0.6988 | **2.6959E-29** | **1.3637E-28** |  | 0.0728 | **0.010** | 0.0525 |  | 0.0026 | 0.1320 | 0.9814 |  | 0.0493 | **0.0280** | 0.0600 |  | 0.0210 | 0.1340 | 0.5025 |  |
| ROI-AFF-NFL-slope of MEM | 0.1405 | **0.0157** | **0.0236** |  | 0.2181 | **0.0061** | **0.0115** |  | -0.0747 | **0.012** | 0.0525 |  | -0.0037 | 0.0840 | 0.9814 |  | -0.0568 | **0.0420** | 0.0840 |  | -0.0142 | 0.3140 | 0.7688 |  |
| ROI-AFF-NFL-slope of LAN | 0.0987 | 0.0907 | 0.0983 |  | 0.1632 | **0.0391** | **0.0465** |  | -0.0689 | **0.038** | 0.0713 |  | -0.0047 | 0.0500 | 0.9814 |  | -0.0731 | **0.0080** | 0.0600 |  | 8.9513E-03 | 0.5200 | 0.9176 |  |
| ROI-AFF-NFL-slope of EF | 0.0964 | 0.0983 | 0.0983 |  | 0.1525 | **0.0562** | **0.0602** |  | -0.0456 | 0.222 | 0.2379 |  | -0.0028 | 0.2160 | 0.9814 |  | -0.0428 | 0.1860 | 0.2143 |  | -6.7656E-05 | 0.9900 | 0.9999 |  |
| ROI-APA-NFL-slope of MMSE | -0.5918 | **1.2308E-29** | **2.4617E-29** |  | -0.6922 | **1.5588E-27** | **4.2512E-27** |  | -0.0607 | **0.028** | 0.0713 |  | -7.4784E-04 | 0.4580 | 0.9814 |  | -0.0382 | 0.1040 | 0.1642 |  | -0.0218 | 0.0960 | 0.4114 |  |
| ROI-APA-NFL-slope of ADAS13 | 0.5991 | **2.5312E-30** | **7.5937E-30** |  | 0.7101 | **1.0276E-29** | **7.7066E-29** |  | 0.049 | 0.056 | 0.0884 |  | 6.5788E-04 | 0.4500 | 0.9814 |  | 0.0261 | 0.2400 | 0.2483 |  | 0.0223 | 0.0560 | 0.3360 |  |
| ROI-APA-NFL-slope of CDRSB | 0.6022 | **5.4196E-31** | **3.2517E-30** |  | 0.6882 | **2.7275E-29** | **1.3637E-28** |  | 0.0835 | **0.004** | **0.0400** |  | 9.8197E-04 | 0.4020 | 0.9814 |  | 0.0516 | **0.0240** | 0.0600 |  | 0.0309 | **0.0060** | 0.0900 |  |
| ROI-APA-NFL-slope of MEM | 0.1405 | **0.0157** | **0.0236** |  | 0.2051 | **0.0101** | **0.0168** |  | -0.0617 | **0.036** | 0.0713 |  | -0.0016 | 0.2940 | 0.9814 |  | -0.0625 | **0.0260** | 0.0600 |  | 0.0024 | 0.8640 | 0.9999 |  |
| ROI-APA-NFL-slope of LAN | 0.0987 | 0.0907 | 0.0983 |  | 0.1654 | **0.0368** | **0.0460** |  | -0.0711 | **0.014** | 0.0525 |  | -0.0018 | 0.2800 | 0.9814 |  | -0.0746 | **0.0080** | 0.0600 |  | 0.0053 | 0.6920 | 0.9999 |  |
| ROI-APA-NFL-slope of EF | 0.0964 | 0.0983 | 0.0983 |  | 0.1474 | 0.0650 | 0.0673 |  | -0.0405 | 0.292 | 0.3021 |  | -0.0011 | 0.4020 | 0.9814 |  | -0.0451 | 0.1740 | 0.21430 |  | 0.0057 | 0.7220 | 0.9999 |  |

Mediation analysis based on structural equation modeling identified three potential mediation pathways: (1) *AD-signature ROI atrophy → NPSs → plasma NFL→ slope of cognition*, (2) *AD-signature ROI atrophy →plasma NFL→ slope of cognition*, and (3) *AD-signature ROI atrophy → NPSs → slope of cognition*. **All analyses were adjusted for age, sex, education, and APOE ε4 status, with intracranial volume additionally included as a covariate in models involving brain structure measures.** Multiple testing significance was corrected using FDR. Significant at the level of *P* < 0.05 were shown in bold. NPSs neuropsychiatric symptoms; HYP hyperactivity; PSY psychosis; AFF affective; APA apathy; MMSE Mini-Mental State Examination; ADAS Alzheimer’s disease Assessment Scale; CDRSB Clinical Dementia Rating Sum of Boxes; MEM Memory function; LAN Language; EF Executive function; NFL neurofilament light chain; ROI region of interest; FDR false discovery rate; CI Confidence Interval.


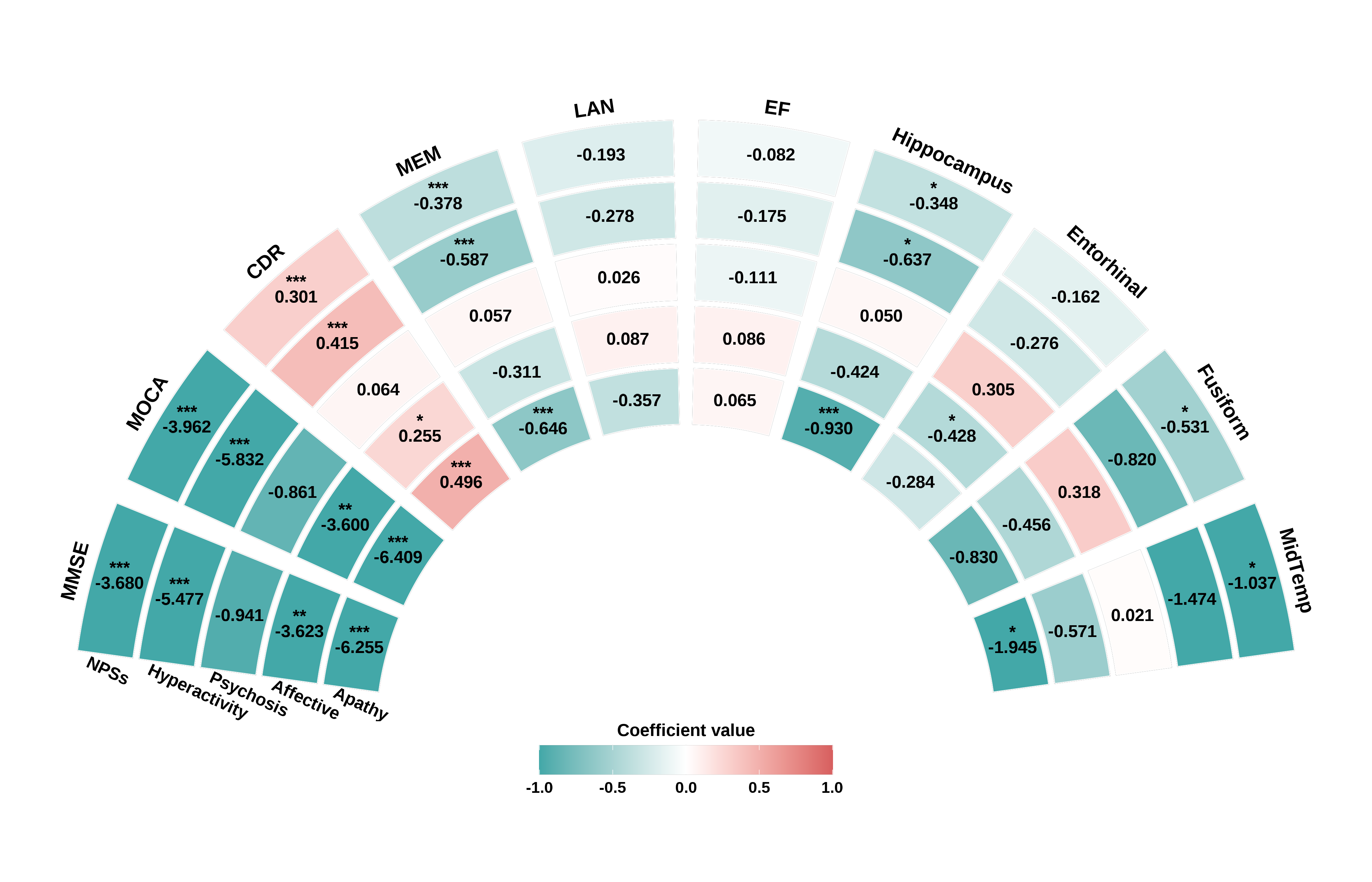


**Figure S1. The cross-sectional associations between NPSs and cognition and brain structures in the discovery cohort.** Associations were analyzed using multiple linear regression, with adjustments for age, sex, education, and APOE ε4 status. Intracranial volume was additionally included as a covariate when brain structure measures were the dependent variable. Multiple testing significance was corrected using false discovery rate. *P < 0.05, **P < 0.01, ***P < 0.001. NPSs neuropsychiatric symptoms; MMSE Mini-Mental State Examination; MoCA Montreal Cognitive Assessment; CDR Clinical Dementia Rating; MEM Memory function; LAN Language; EF Executive function; *APOE* ε4 apolipoprotein ε4.


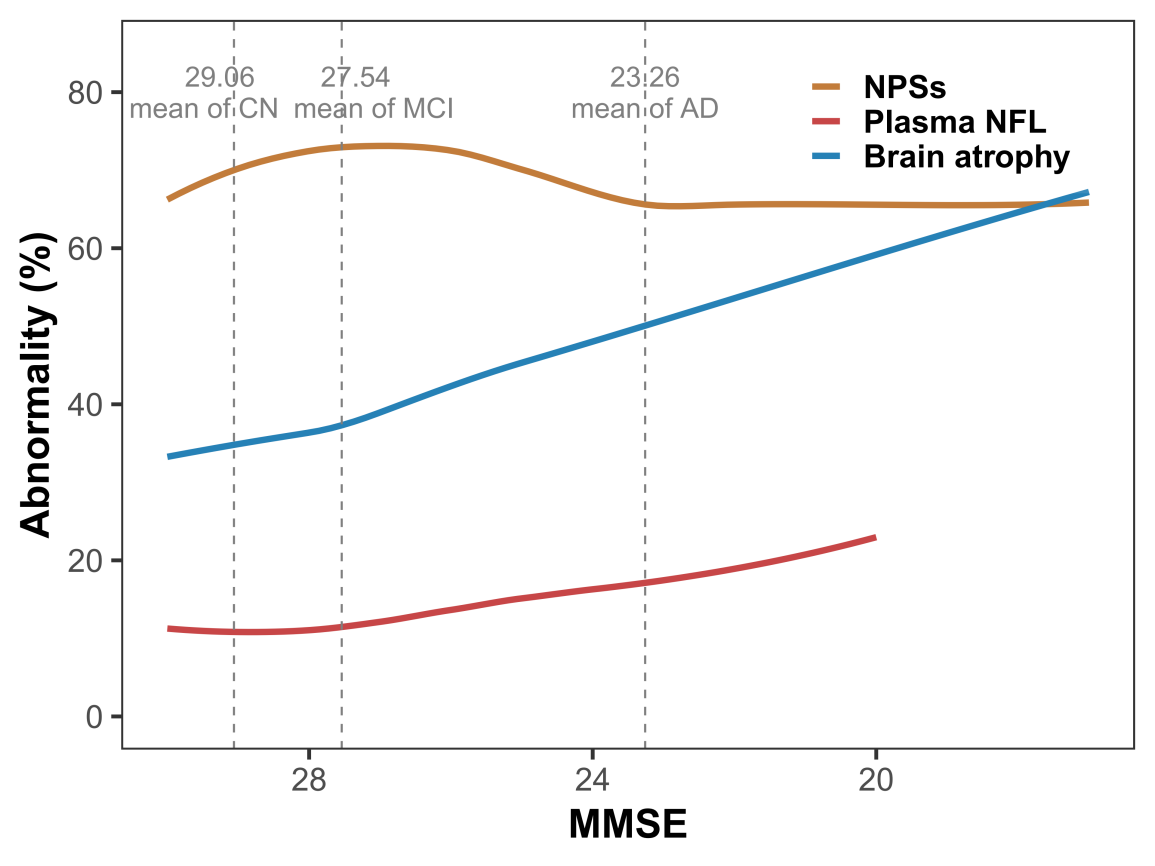


**Figure S2.** **LOESS trajectories of NPSs, plasma NFL, and brain atrophy across AD clinical stages**. Marker values were normalized to a 0–100% abnormality scale using min‑max scaling. LOESS regression was applied to model the dynamic trajectories of each marker as a function of disease severity. MMSE was used as the anchor, with lower MMSE scores indicating more advanced disease (CN mean = 29.06, MCI mean = 27.54, AD mean = 23.26). NPSs showed early elevation when MMSE was approximately 28–29, corresponding to preclinical or early MCI stages, whereas plasma NFL and brain atrophy increased more gradually and became prominent at later stages. These findings are hypothesis‑generating and suggest that NPSs precedes neuroaxonal damage and structural changes along the disease continuum, consistent with a potential temporal cascade, but LOESS alone does not establish causation. LOESS locally estimated scatterplot smoothing; NPSs neuropsychiatric symptoms; NFL neurofilament light chain; MMSE Mini-Mental State Examination; CN cognitively normal; MCI mild cognitive impairment; AD Alzheimer’s Disease.

**References**

Gmitrowicz, A., & Kucharska, A. (1994). [Developmental disorders in the fourth edition of the American classification: diagnostic and statistical manual of mental disorders (DSM IV -- optional book)]. *Psychiatria Polska,* **28**(5), 509-521.

Lu, J., Li, D., Li, F., Zhou, A., Wang, F., Zuo, X., . . . Jia, J. (2011). Montreal cognitive assessment in detecting cognitive impairment in Chinese elderly individuals: a population-based study. *Journal of Geriatric Psychiatry and Neurology,* **24**(4), 184-190. <https://doi:10.1177/0891988711422528>
